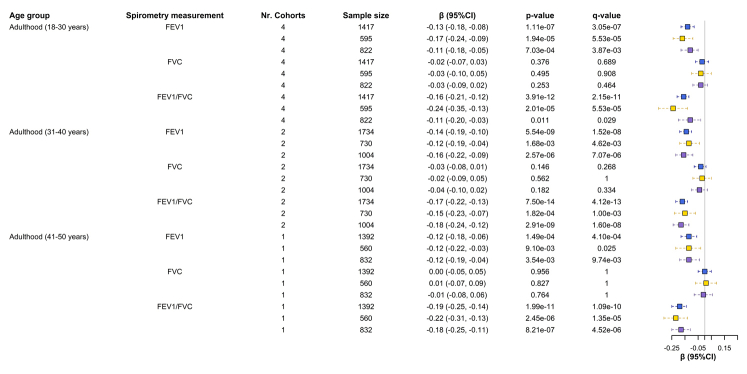# Exploring the genetics of airflow limitation in lung function across the lifespan – a polygenic risk score study

**DOI:** 10.1016/j.eclinm.2024.102731

**Published:** 2024-08-12

**Authors:** Natalia Hernandez-Pacheco, Anna Kilanowski, Ashish Kumar, John A. Curtin, Núria Olvera, Sara Kress, Xander Bertels, Lies Lahousse, Laxmi Bhatta, Raquel Granell, Sergi Marí, Jose Ramon Bilbao, Yidan Sun, Casper-Emil Tingskov Pedersen, Tarik Karramass, Elisabeth Thiering, Christina Dardani, Simon Kebede Merid, Gang Wang, Jenny Hallberg, Sarah Koch, Judith Garcia-Aymerich, Ana Esplugues, Maties Torrent, Jesus Ibarluzea, Lesley Lowe, Angela Simpson, Ulrike Gehring, Roel C.H. Vermeulen, Graham Roberts, Anna Bergström, Judith M. Vonk, Janine F. Felix, Liesbeth Duijts, Klaus Bønnelykke, Nic Timpson, Guy Brusselle, Ben M. Brumpton, Arnulf Langhammer, Stephen Turner, John W. Holloway, Syed Hasan Arshad, Anhar Ullah, Adnan Custovic, Paul Cullinan, Clare S. Murray, Maarten van den Berge, Inger Kull, Tamara Schikowski, Jadwiga A. Wedzicha, Gerard Koppelman, Rosa Faner, Àlvar Agustí, Marie Standl, Erik Melén, CADSET Clinical Research Collaboration of the European Respiratory Society

**Affiliations:** a Department of Clinical Science and Education, Södersjukhuset, Karolinska Institutet, Sjukhusbacken 10, 118 83, Stockholm, Sweden; b Institute of Epidemiology, Helmholtz Zentrum München – German Research Center for Environmental Health, Campus Neuherberg, Ingolstädter Landstraße 1, 85764, Neuherberg, Germany; c Division of Metabolic and Nutritional Medicine, Dr. von Hauner Children's Hospital, University of Munich Medical Center, Lindwurmstraße 4, 80337, Munich, Germany; d Division of Infection, Immunity and Respiratory Medicine, School of Biological Sciences, The University of Manchester, Manchester Academic Health Science Centre, and Manchester University NHS Foundation Trust, Cobbett House Manchester Royal Infirmary, Oxford Rd, Manchester, M13 9WL, United Kingdom; e CIBER de Enfermedades Respiratorias (CIBERES), Spain; f Universitat de Barcelona, Departament de Biomedicina, Institut D'investigacions Biomediques August Pi I Sunyer (IDIBAPS), Calle Rosselló 149, 08036, Barcelona, Spain; g IUF – Leibniz Research Institute for Environmental Medicine, Auf'm Hennekamp 50, 40225, Düsseldorf, Germany; h Department of Bioanalysis, Faculty of Pharmaceutical Sciences, Ghent University, Ottergemsesteenweg 460, 9000, Ghent, Belgium; i Department of Epidemiology, Erasmus MC, University Medical Center Rotterdam, PO Box 2040, Rotterdam, 3000, CA, the Netherlands; j K.G. Jebsen Center for Genetic Epidemiology, Department of Public Health and Nursing, NTNU, Norwegian University of Science and Technology, Håkon Jarls gt.11, 7491, Trondheim, Norway; k HUNT Research Centre, Department of Public Health and Nursing, Faculty of Medicine and Health Sciences, NTNU, S.P. Andersens veg 11, 7031, Trondheim, Norway; l Division of Mental Health Care, St. Olavs Hospital, Trondheim University Hospital, Olav Kyrres gate 9, 7030, Trondheim, Norway; m Medical Research Council Integrative Epidemiology Unit (MRC-IEU), Population Health Sciences, Bristol Medical School, Faculty of Health Sciences, University of Bristol, 5 Tyndall Ave, Bristol, BS8 1UD, United Kingdom; n Biobizkaia Health Research Institute, University of the Basque Country (UPV/EHU), Leioa, 48940, Bizkaia, Spain; o CIBER Diabetes y Enfermedades Metabólicas asociadas (CIBEDEM), Spain; p University of Groningen, University Medical Center Groningen, Beatrix Children's Hospital, Department of Pediatric Pulmonology and Pediatric Allergology, Hanzeplein 1, 9713 GZ, Groningen, the Netherlands; q COPSAC, Copenhagen Prospective Studies on Asthma in Childhood, Herlev and Gentofte Hospital, Ledreborg alle 34, 2820, Gentofte, Denmark; r The Generation R Study Group, Erasmus MC, University Medical Center, Dr. Molewaterplein 40, 3015 GD, Rotterdam, the Netherlands; s Department of Pediatrics, Division of Respiratory Medicine and Allergology, Erasmus MC, University Medical Center, Dr. Molewaterplein 40, 3015 GD, Rotterdam, the Netherlands; t Institute of Integrated Traditional Chinese and Western Medicine, West China Hospital, Sichuan University, 17 Renmin South Rd Section 3, 小天竺 Wuhou District, Chengdu, Sichuan, 610041, China; u Sachs' Children and Youth Hospital, Södersjukhuset, Hjalmar Cederströms gata 14, 118 61 Stockholm, Sweden; v ISGlobal, Barcelona, Spain; w Universitat Pompeu Fabra, Barcelona, Spain; x CIBER Epidemiología y Salud Pública (CIBERESP), Spain; y Department of Nursing, University of Valencia, Avenida de Menéndez y Pelayo, 19, 46010 Valencia, Spain; z FISABIO-Universitat Jaume I-Universitat de València Joint Research Unit of Epidemiology and Environmental Health, Av. de Catalunya, 21, 46020, Valencia, Spain; aa Ib-Salut, Area de Salut de Menorca, Palma, Spain; ab Biodonostia Health Research Institute, Group of Environmental Epidemiology and Child Development, Paseo Doctor Begiristain S/n, 20014, San Sebastian, Spain; ac Department of Health of the Basque Government, Subdirectorate of Public Health of Gipuzkoa, Avenida Navarra 4, 20013, San Sebastian, Spain; ad Faculty of Psychology, University of the Basque Country (UPV/EHU), 20008, San Sebastian, Spain; ae Institute for Risk Assessment Sciences, Utrecht University, Utrecht, the Netherlands; af David Hide Asthma and Allergy Research Centre, St Marys Hospital Nhs Trust, Newport, PO30 5TG, United Kingdom; ag NIHR Southampton Biomedical Research Centre, University Hospitals Southampton NHS Foundation Trust, Tremona Road, Southampton, SO16 6YD, United Kingdom; ah Human Development and Health, Faculty of Medicine, University of Southampton, 12 University Rd, Southampton, SO17 1BJ, United Kingdom; ai Institute of Environmental Medicine, Karolinska Institutet, Nobels väg 13, 171 65, Solna, Stockholm, Sweden; aj Centre for Occupational and Environmental Medicine, Region Stockholm, Torsplan, Solnavägen 4, 113 65, Stockholm, Sweden; ak Department of Epidemiology, University Medical Center Groningen, University of Groningen, Hanzeplein 1, 9713 GZ, Groningen, the Netherlands; al Groningen Research Institute for Asthma and COPD (GRIAC), University of Groningne, University Medical Center Groningen, Hanzeplein 1, 9713 GZ, Groningen, the Netherlands; am Department of Pediatrics, Erasmus MC, University Medical Center, Dr. Molewaterplein 40, 3015 GD, Rotterdam, the Netherlands; an Department of Respiratory Medicine, Ghent University Hospital, Corneel Heymanslaan 10, 9000, Ghent, Belgium; ao Departments of Epidemiology and Respiratory Medicine, Erasmus MC, University Medical Center Rotterdam, PO Box 2040, Rotterdam, 3000, CA, the Netherlands; ap Department of Levanger Hospital, Nord-Trøndelag Hospital Trust, Helse Nord-Trøndelag, 7601, Levanger, Norway; aq Royal Aberdeen Children's Hospital NHS Grampian, Westburn Rd, Aberdeen, AB25 2ZG, United Kingdom; ar Clinical and Experimental Sciences, Faculty of Medicine, University of Southampton, 12 University Rd, Southampton, SO17 1BJ, United Kingdom; as National Heart and Lung Institute, Imperial College London, St Mary's Campus Medical School, Norfolk Place, London W2 1PG, United Kingdom; at Cátedra de Salud Respiratoria, University of Barcelona, Calle Casanovas, 143, 08036, Barcelona, Spain; au Pulmonary Service, Respiratory Institute, Hospital Clinic, Calle Villarroel, 170, 08036, Barcelona, Spain; av German Center for Lung Research (DZL), Aulweg 130, 35392, Gießen, Munich, Germany

**Keywords:** Polygenic risk score, Genetics, Chronic obstructive pulmonary disease, Lung function

## Abstract

**Background:**

Chronic obstructive pulmonary disease (COPD) is caused by interactions between many factors across the life course, including genetics. A proportion of COPD may be due to reduced lung growth in childhood. We hypothesized that a polygenic risk score (PRS) for COPD is associated with lower lung function already in childhood and up to adulthood.

**Methods:**

A weighted PRS was calculated based on the 82 association signals (*p* ≤ 5 × 10^−8^) revealed by the largest GWAS of airflow limitation (defined as COPD) to date. This PRS was tested in association with lung function measures (FEV_1_, FVC, and FEV_1_/FVC) in subjects aged 4–50 years from 16 independent cohorts participating in the Chronic Airway Diseases Early Stratification (CADSET) Clinical Research Collaboration. Age-stratified meta-analyses were conducted combining the results from each cohort (n = 45,406). These findings were validated in subjects >50 years old.

**Findings:**

We found significant associations between the PRS for airflow limitation and: *(1)* lower pre-bronchodilator FEV_1_/FVC from school age (7–10 years; β: −0.13 z-scores per one PRS z-score increase [–0.15, −0.11], *q*-value = 7.04 × 10^−53^) to adulthood (41–50 years; β: −0.16 [–0.19, −0.13], *q*-value = 1.31 × 10^−24^); and *(2)* lower FEV_1_ (from school age: 7–10 years; β: −0.07 [–0.09, −0.05], *q*-value = 1.65 × 10^−9^, to adulthood: 41–50 years; β: −0.17 [–0.20, −0.13], *q*-value = 4.48 x 10^−20^). No effect modification by smoking, sex, or a diagnosis of asthma was observed.

**Interpretation:**

We provide evidence that a higher genetic risk for COPD is linked to lower lung function from childhood onwards.

**Funding:**

This study was supported by CADSET, a Clinical Research Collaboration of the 10.13039/100008593European Respiratory Society.

**Research in context**

**Evidence before this study**

We performed a search on PubMed using the terms (“PRS” or “polygenic risk score”) AND (“chronic obstructive pulmonary disease” or “COPD”) AND (“lung function” or “spirometry”) in the title and abstract up to April 23, 2024. Several publications have investigated the implication of genetic variants combined into a polygenic risk score (PRS) in the development of chronic obstructive pulmonary disease (COPD). This has been linked to lower lung function levels mainly in adults. Our understanding of the influence of genetic susceptibility for COPD on lung function across the life course is still incomplete.

**Added value of this study**

This study reports the association of a PRS for airflow limitation with lower FEV_1_/FVC and FEV_1_ across age groups in several large cohorts, primarily based on longitudinal data. The association effect was not observed to be modified by tobacco smoking habits, sex, or asthma diagnosis. These findings provide firm evidence of the role of genetic factors for COPD susceptibility in lung function not only in adults but also in childhood and adolescence.

**Implications of all the available evidence**

A higher genetic risk of developing COPD in combination with other factors is linked to lower lung function from childhood onwards. These results strongly support previous suggestions of considering the mechanisms underlying COPD pathophysiology operating across the entire life course. This has important implications for preventing the development of COPD as early in life as possible.

## Introduction

In healthy people, lung development starts *in utero* and continues after birth until lung function reaches its peak in early adulthood. In normal physiological aging, lung function starts to decline from approximately 25 years of age. Thus, lower lung function during early life resulting in a lower peak lung function may persist in adult life.^1^ This leads to a sub-optimal lung function trajectory during the life course^2^^,^^3^ that has been associated with obstructive airway diseases such as asthma and chronic obstructive pulmonary disease (COPD).^4^^,^^5^ COPD is the most common non-communicable respiratory disease in adults. Although with wide variations in prevalence, it has been estimated as 10.3% of the global population^6^ and causing up to three million global fatalities annually.^6^ It is characterized by chronic respiratory symptoms caused by airway and/or alveolar abnormalities that lead to persistent, non-fully reversible airflow limitation^7^ and is usually diagnosed in late adulthood.^8^ COPD can arise from a mixture of several lifetime exposures, including tobacco smoking as well as social and host factors that damage the lungs and alter the normal developmental or physiological aging processes.^9^

Genetic variation significantly influences the clinical presentation of COPD, including disease susceptibility, phenotypic heterogeneity, severity of airflow limitation, and frequency of acute exacerbations.^10^^,^^11^ Heritability estimates have been reported at 40% in independent individuals and up to 60% in twin studies.^11^ The most recent and largest genome-wide association study (GWAS) of susceptibility for COPD in adults (n = 257,811) identified a total of 82 independent single nucleotide polymorphisms (SNPs) associated at the genome-wide significance level (*p* ≤ 5 × 10^−8^).^12^ The authors defined cases and controls following the spirometry-based criteria for moderate-to-severe airflow limitation.^6^

The early origins of health and disease hypothesis^13^ suggest that the mechanisms leading to COPD can originate in pre-conception stages, gestation, and the first years of life,^14^^,^^15^ albeit usually, it does not show clinical manifestations until adulthood.^6^ Furthermore, individual genetic liability is established since conception, suggesting that the genetic susceptibility for COPD also begins in early life.^11^ Polygenic risk scores (PRSs) for COPD are used to precisely estimate individual genetic susceptibility to this disease in a more powerful approach as it combines separate relatively small effect sizes into a genome-wide quantitative estimate of relative individual genetic risk of COPD.^16^, ^17^, ^18^ Additionally, a previous PRS for COPD had been associated with an earlier age of diagnosis of COPD.^19^

Considering this, the present study sought to evaluate the contribution of genetic factors of COPD to lung function across the life course, by assessing whether a weighted PRS for airflow limitation is associated with lower lung function in different age groups. To do this, we first calculated and internally validated a PRS in independent studies based on genetic variants previously associated with COPD in adults, defined as airflow limitation, by the largest GWAS published to date.^12^ Secondly, we evaluated the association of this PRS with spirometry indices from preschool age to adulthood.

## Methods

### Study design and ethics

Sixteen independent cohorts, part of the Chronic Airway Diseases Early Stratification (CADSET) Clinical Research Collaboration^20^ from the European Respiratory Society (ERS), participated in this study (Table S1). Subjects aged 4–50 years of cross-sectional or longitudinal studies were included. Additional validation of the association with lung function was conducted in older individuals (>50 years from two of these independent cohorts). Written informed consent was obtained from all participants or their legal guardians in each cohort in accordance with the Declaration of Helsinki for all the participating studies.

A detailed description of each participating cohort and the methodology used for the analyses described below is provided in the Supplementary Methods.

### Genome-wide genotyping, assessment of genetic ancestry, and genotype imputation

Samples from each cohort had been genome-wide genotyped using different platforms for previous investigations (Table S1). Standard quality control (QC) procedures for GWAS analyses were applied, and genetic ancestry was assessed independently in each cohort through a Principal Component (PC) analysis using PLINK,^21^ or the *SeqArray* and *SNPRelate* R packages.^22^ Imputation of genetic variants was conducted by different standardized approaches (Table S1).

### Calculation of a polygenic risk score for airflow limitation

**Fig. 1:**
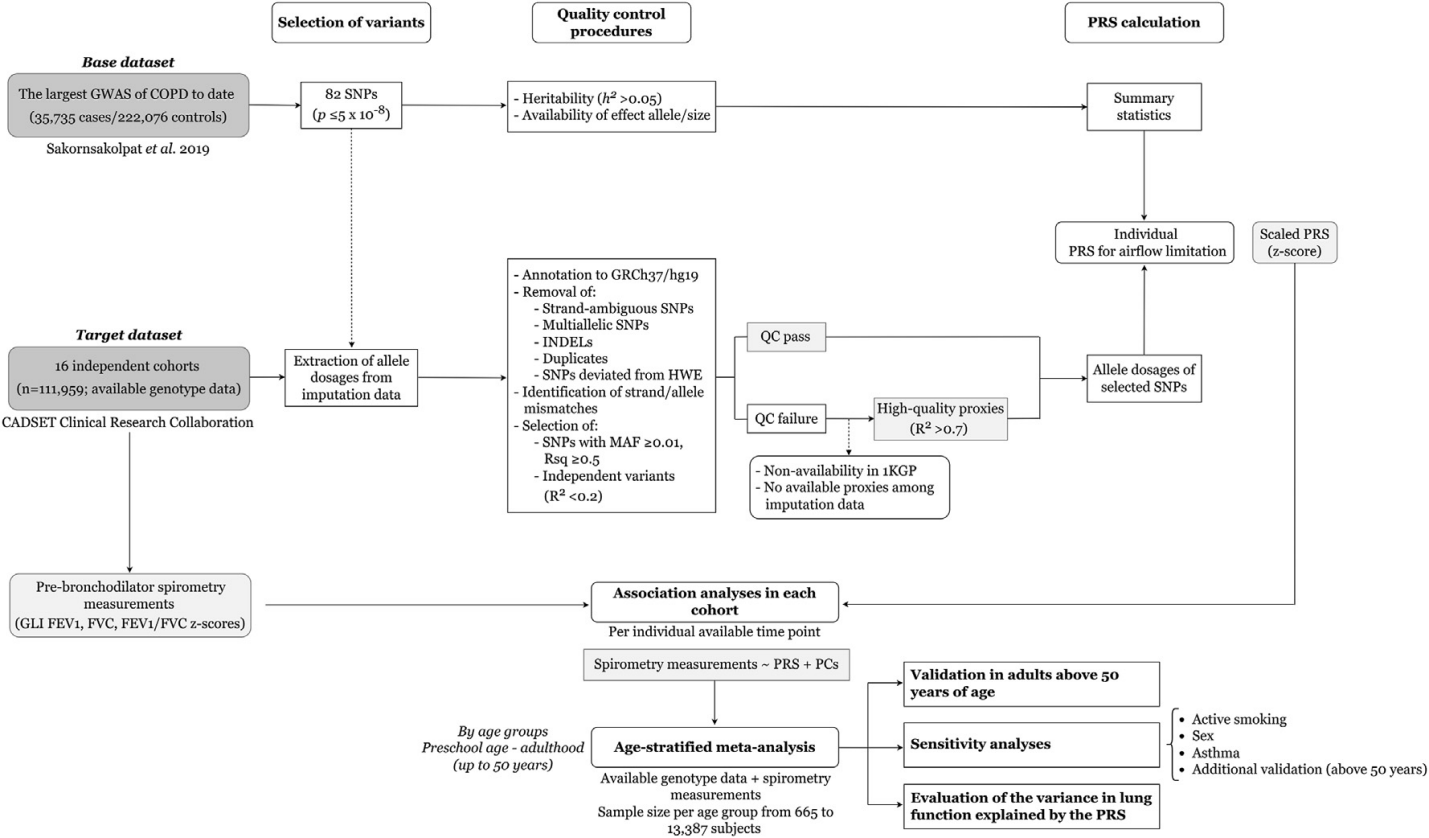
**Flowchart of the methodology used for the calculation of the PRS for airflow limitation and its evaluation with spirometry measurements across the lifespan.** A total of 82 SNPs associated at the genome-wide significance level (*p-*value *≤*5 × 10^−8^) by the largest GWAS of COPD susceptibility published to date^12^ were initially selected for the PRS estimation in the current study. Several QC procedures were conducted in the base dataset and each of the cohorts that were part of the target dataset. The association between transformed PRS estimates (z-scores) and GLI z-scores of pre-bronchodilator spirometry measurements was assessed through linear regressions at the different available time points per cohort. The association results from each cohort were combined in an age-stratified meta-analysis by age groups from preschool age to adulthood (up to 50 years of age). Validation of these results was conducted in subjects older than 50 years. Moreover, the potential effect of active smoking, sex, and asthma was evaluated through sensitivity analyses. The proportion of lung function variance explained by the PRS was estimated in large sample-sized cohorts across different age groups. 1KGP: 1000 Genomes Project reference panel; CADSET: Chronic Airway Diseases Early Stratification; COPD: chronic obstructive pulmonary disease; GLI: Global Lung Function Initiative; FEV_1_: forced respiratory volume in 1 s; FVC: forced vital capacity; GWAS: genome-wide association study; HWE: Hardy–Weinberg equilibrium; INDELs: insertions/deletions; MAF: minor allele frequency; PC: Principal Component of genetic ancestry; PRS: polygenic risk score; QC: quality control; Rsq: imputation quality score; SNP: single nucleotide polymorphism.

Eighty-two SNPs independently associated with susceptibility for adult COPD, defined as airflow limitation (pre-bronchodilator FEV_1_ less than 80% of predicted value and the ratio between FEV_1_ and FVC less than 0.7), that reached the standard genome-wide significance level (*p-*value ≤5 × 10^−8^) in the large-scale GWAS (35,735 cases, 222,076 controls) performed by Sakornsakolpat et al.^12^ were initially selected for the calculation of a PRS for airflow limitation. These SNPs and their summary statistics were considered as the base dataset for the current study (Table S2). For each participant, weighted PRS estimates were independently calculated in each of the 16 CADSET cohorts representing the target dataset. First, several QC procedures were carried out in the base dataset, and each CADSET cohort^23^ (Fig. 1). Second, PRS estimates were calculated by summing the allele dosage of the genetic variants that passed the QC criteria out of the 82 initially selected or available proxies in each study, weighted by the effect size of the association between each SNP and COPD susceptibility in the base dataset. Finally, the PRS for airflow limitation obtained was scaled by its transformation into z-scores for interpretation in each cohort. All participants with available imputed genome-wide genotyping data regardless of corresponding clinical information were included in the PRS calculation to maximize predictability. These analyses were carried out following standard guidelines for PRS calculation,^23^ using R version ≥3.6.0.^22^

### Lung function measurements

Forced spirometry was determined following the guidelines established by the American Thoracic Society (ATS) and ERS.^24^ The values of the forced expiratory volume in 1 s (FEV_1_), forced vital capacity (FVC), and the ratio FEV_1_/FVC before bronchodilation were converted into z-scores using the equations from the Global Lung Function Initiative (GLI).^25^

### Evaluation of the association between the PRS for airflow limitation and spirometry measurements

The contribution of the PRS for airflow limitation on the spirometry measurements obtained across the lifespan was independently investigated in each CADSET cohort by exploring the association of the PRS with FEV_1_, FVC, and FEV_1_/FVC z-scores (Fig. 1). This was carried out for every time point with available spirometry data per cohort. A linear regression model adjusted for PCs of genetic ancestry and any cohort-specific variables was defined as the basic association model. Participants with PRS estimates and spirometry measures available at the time point evaluated were included in these analyses.

Association results with lung function data from each cohort were combined in an inverse-variance meta-analysis by groups of similar age ranging from preschool age to adulthood based on data availability (Table S3, Supplementary Methods). The evidence of significant association was considered after a false discovery rate (FDR) adjustment of 5% (*q*-value≤0.05) across spirometry measurements per age group. Validation analyses of the association of the PRS for airflow limitation with spirometry measurements were carried out in adults older than 50 years, which is an age group when COPD is more prevalent and a greater detriment of the lung function caused by normal physiological aging is expected. This was conducted in two independent cohorts, including one that had participated in the GWAS of COPD from the base dataset.^12^ Evidence of replication was considered accounting for the direction of effect between the PRS for airflow limitation and spirometry measurements. The proportion of variance in lung function explained by the PRS was estimated through cross-validation utilizing the *caret* R package.^22^^,^^26^ This was performed in studies with the largest sample size across age groups as a representation of the whole set of participating cohorts (Fig. 1).

Sensitivity analyses were carried out to assess the potential effect of smoking in adult participants, sex, and asthma on the association between the PRS for airflow limitation and lung function using different approaches (Fig. 1).

### Role of the funding source

The study sponsors had no role in the study design, data collection, data analysis, data interpretation, or report writing. The co-authors in charge of the analyses of this study and cohort representatives had access to their own cohort dataset and all co-authors had final responsibility for publication decisions.

## Results

### Characteristics of the study populations

The PRS for airflow limitation was calculated in a total of 111,959 individuals participating in 16 independent CADSET studies. Most participants were of European descent, except for one of the cohorts which included 7% of individuals from non-European ancestry. The association between the PRS and spirometry measurements was explored in subjects aged up to 50 years by age groups with a sample size ranging from 665 (preschool age) to 13,387 individuals (school age) (Table S3). Additionally, validation of the association of the PRS with lung function was conducted in subjects >50 years of age from two cohorts. A total of 9,027 participants were included from one of them, and between 741 and 5,722 individuals whose spirometry was measured at three different time points from the other cohort. Table 1 describes a summary of the clinical and demographic characteristics of participants from each cohort.

**Table 1:** Clinical and demographic characteristics of each cohort and time point included in this study.

Cohort	n_PRS_^a^	Median PRS z-score (IQR)^b^	Time point^c^	n_Association_^d^	Sex (% male)	Smoking (%)^e^	Asthma (%)^f^	Mean age ± SD (years)	Mean FEV_1_ z-score ± SD^g^	Mean FVC z-score ± SD^g^	Mean FEV_1_/FVC z-score ± SD^g^
ALSPAC	8943	0.00 (−0.66 to 0.66)	8 years	4871	50.4	NA	14.7	8.7 ± 0.3	−0.1 ± 1.0	−0.1 ± 1.0	0.0 ± 1.1
			15 years	3332	48.1	NA	13.9	15.5 ± 0.3	−0.7 ± 1.3	−0.9 ± 1.2	0.4 ± 1.2
			24 years	2590	39.7	19.6	17.5	24.5 ± 0.8	−0.4 ± 1.0	−0.2 ± 1.0	−0.4 ± 0.9
Ashford	348	−0.03 (−0.69 to 0.68)	15 years	322	50.6	NA	20.8	15.0 ± 0.0	−0.7 ± 1.1	−0.7 ± 1.1	0.0 ± 1.1
BAMSE^h^	463^i^	−0.01 (−0.64 to 0.65)	8 years	335	54.6	NA	31.9	8.3 ± 0.5	0.4 ± 0.9	0.6 ± 0.9	−0.5 ± 0.9
			16 years	305	52.8	NA	31.8	16.7 ± 0.4	−0.2 ± 0.9	0.1 ± 0.9	−0.5 ± 1.0
			24 years	282	50.7	17.5	22.7	22.5 ± 0.5	−0.3 ± 0.8	0.0 ± 0.8	−0.5 ± 0.9
	2173^j^	0.02 (−0.69 to 0.66)	8 years	1230	48.5	NA	7.6	8.4 ± 0.5	0.5 ± 0.9	0.6 ± 0.9	−0.3 ± 0.9
			16 years	1185	44.6	NA	13.4	16.7 ± 0.4	0.0 ± 1.0	0.2 ± 0.9	−0.3 ± 0.9
			24 years	1044	43.3	18.4	10.2	22.6 ± 0.6	−0.2 ± 0.9	0.0 ± 0.9	−0.3 ± 0.9
COPSAC_2000_	358	0.00 (−0.67 to 0.66)	7 years	292	50.3	NA	13.4	7.1 ± 0.4	0.0 ± 1.0	0.0 ± 1.0	0.0 ± 1.0
			12 years	293	48.5	NA	17.9	12.8 ± 0.6	0.0 ± 1.0	0.0 ± 1.0	0.0 ± 1.0
			18 years	317	47.9	17.3	29.7	17.7 ± 0.6	0.0 ± 1.0	0.0 ± 1.0	0.0 ± 1.0
COPSAC_2010_	618	−0.03 (−0.67 to 0.69)	10 years	530	52.3	NA	10.6	10.3 ± 0.4	0.0 ± 1.0	0.0 ± 1.0	0.0 ± 1.0
Generation R	5756	0.00 (−0.68 to 0.67)	9 years	2147	48.9	NA	3.5	9.6 ± 0.3	0.1 ± 1.0	0.2 ± 0.9	−0.2 ± 0.9
			13 years	1897	48.1	NA	5.3	13.6 ± 0.3	−0.3 ± 1.0	−0.2 ± 0.9	−0.2 ± 0.9
GINIplus/LISA North	792	−0.03 (−0.69 to 0.70)	10 years	374	52.9	NA	7.5	10.3 ± 0.2	0.1 ± 0.8	−0.4 ± 0.8	0.9 ± 1.0
			15 years	496	48.6	NA	5.1	15.2 ± 0.3	−0.7 ± 0.9	−0.6 ± 0.9	−0.2 ± 0.9
GINIplus/LISA South	1511	0.00 (−0.71 to 0.67)	6 years	106	48.1	NA	5.3	6.1 ± 0.1	−0.1 ± 0.9	−0.6 ± 0.9	1.3 ± 0.8
			15 years	843	49.5	NA	7.5	15.3 ± 0.3	−0.4 ± 0.9	−0.5 ± 0.9	0.0 ± 1.0
HUNT	69,717	0.00 (−0.67 to 0.68)	20–30 years^k^	2848	43.3	31.9	37.2	26.0 ± 3.1	−0.2 ± 1.0	0.2 ± 0.9	−0.5 ± 0.9
			31–40 years^k^	3107	45.1	33.7	40.8	36.4 ± 2.9	−0.2 ± 1.1	0.1 ± 1.0	−0.5 ± 1.0
			41–50 years^k^	4142	46	35.4	38.1	46.0 ± 2.9	−0.2 ± 1.2	0.0 ± 1.0	−0.4 ± 1.0
			>50 years^k^	9027	47.4	27.2	36.2	63.4 ± 8.9	−0.6 ± 1.4	−0.3 ± 1.1	−0.6 ± 1.2
INMA	2034	−0.04 (−0.67 to 0.66)	4 years	559	51.7	NA	NA	4.5 ± 0.1	−0.6 ± 1.2	−0.6 ± 1.3	0.0 ± 1.0
			7 years	925	50.4	NA	NA	7.4 ± 0.6	0.2 ± 1.0	0.4 ± 0.9	−0.4 ± 1.0
			10 years	65	58.5	NA	NA	10.6 ± 0.2	−0.2 ± 1.0	0.1 ± 1.0	−0.5 ± 0.9
			11 years	792	51	NA	NA	11.2 ± 0.6	−0.2 ± 1.0	0.0 ± 1.0	−0.3 ± 1.0
			14 years	188	51.6	NA	NA	14.6 ± 0.2	0.1 ± 1.0	0.0 ± 1.0	0.1 ± 0.9
			18 years	87	35.6	20.7	NA	17.7 ± 0.3	−0.4 ± 1.0	−0.2 ± 1.0	−0.3 ± 1.0
IoWBC	956	−0.01 (−0.72 to 0.70)	10 years	754	49.3	NA	23.3	10.0 ± 0.2	0.3 ± 1.0	0.2 ± 0.9	0.3 ± 1.0
			18 years	669	46.2	25.2	20.2	17.7 ± 0.5	0.1 ± 1.0	0.1 ± 0.9	0.0 ± 1.1
			26 years	432	44.2	NA	NA	26.4 ± 0.4	0.0 ± 1.0	0.4 ± 1.0	−0.7 ± 0.9
Lifelines	859	0.01 (−0.71 to 0.66)	18–30 years^k^	859	44.1	35	12.2	27.1 ± 2.6	−0.4 ± 0.9	−0.2 ± 0.9	−0.4 ± 1.0
	3005	−0.01 (−0.66 to 0.65)	31–40 years^k^	3005	40.9	26.7	9.1	36.4 ± 2.8	−0.4 ± 0.9	−0.2 ± 0.9	−0.4 ± 1.0
MAAS	852	0.01 (−0.67 to 0.70)	8 years	640	53	NA	12.2	8.0 ± 0.2	0.1 ± 1.0	0.3 ± 1.0	−0.4 ± 1.0
			16 years	502	51	NA	14.3	16.1 ± 0.6	−0.3 ± 1.0	−0.4 ± 1.0	0.2 ± 1.1
			19 years	436	48	13.3	7.3	19.4 ± 0.8	−0.3 ± 1.0	−0.3 ± 0.9	0.0 ± 1.1
PIAMA	1526	−0.03 (−0.68 to 0.68)	8 years	907	50.4	NA	10	8.1 ± 0.3	0.5 ± 0.9	0.3 ± 0.9	0.3 ± 1.1
			12 years	1018	48.3	NA	9.2	12.6 ± 0.4	−0.6 ± 0.9	−0.4 ± 0.9	−0.4 ± 0.9
			16 years	653	47.9	NA	8.3	16.4 ± 0.2	−0.3 ± 0.9	0.0 ± 0.8	−0.5 ± 1.0
Rotterdam Study	11,496	0.00 (−0.68 to 0.69)	PFT 1 (50–98 years)^l^	5722	44.1	14.8	6	67.5 ± 9.3	−0.1 ± 1.2	−0.1 ± 1.1	−0.1 ± 1.0
			PFT 2 (51–96 years)^l^	3317	44.3	10.4	7.2	70.7 ± 9.1	−0.1 ± 1.1	0.0 ± 0.9	−0.2 ± 0.9
			PFT 3 (70–100 years)^l^	741	44.9	6.2	9.2	78.9 ± 5.2	−0.1 ± 1.1	0.1 ± 0.9	−0.6 ± 0.9
SEATON	552	0.05 (−0.68 to 0.71)	10 years	382	47.1	NA	13.4	10.3 ± 0.2	−0.2 ± 1.1	−0.3 ± 1.0	0.1 ± 1.0
			15 years	330	43	NA	NA	15.1 ± 0.3	−0.4 ± 1.0	−0.8 ± 1.0	0.8 ± 1.1

FEV_1_: forced expiratory volume in 1 s; FVC: forced vital capacity; IQR: interquartile range; NA: not available; PFT: pulmonary function test visit; PRS: polygenic risk score; SD: standard deviation.

a Number of individuals with available genome-wide genotype data included in the estimation of the PRS for airflow limitation.

b Median, quartile 1 and 3 of normalized PRS estimates into z-scores.

c Average age of the individuals at the data collection follow-ups included in this study.

d Number of individuals with available genotype data and spirometry measurements included in the association analyses.

e Percentage of individuals with reported active smoking habits. Only shown for time points with participants aged 18 years and older with available data related to smoking habits.

f Number of subjects with asthma at the time of data collection defined as doctor's diagnosis of asthma and any symptoms with breathing difficulties or occasional or regular use of asthma medications in the last 12 months, or self-reports.

g Obtained from applying the Global Lung Function Initiative equations on pre-bronchodilator spirometry measurements.

h The PRS was separately estimated in each of the genotyping Waves of the BAMSE cohort (Wave 1, n = 463; Wave 2, n = 2173).

i Genotyping Wave 1 (n = 463).

j Genotyping Wave 2 (n = 2173).

k Subjects were classified into groups of similar age for this study.

l Participants have been grouped based on the time point when the pulmonary function test was conducted.

### Association of the PRS for airflow limitation with spirometry measurements across the lifespan

**Fig. 2:**
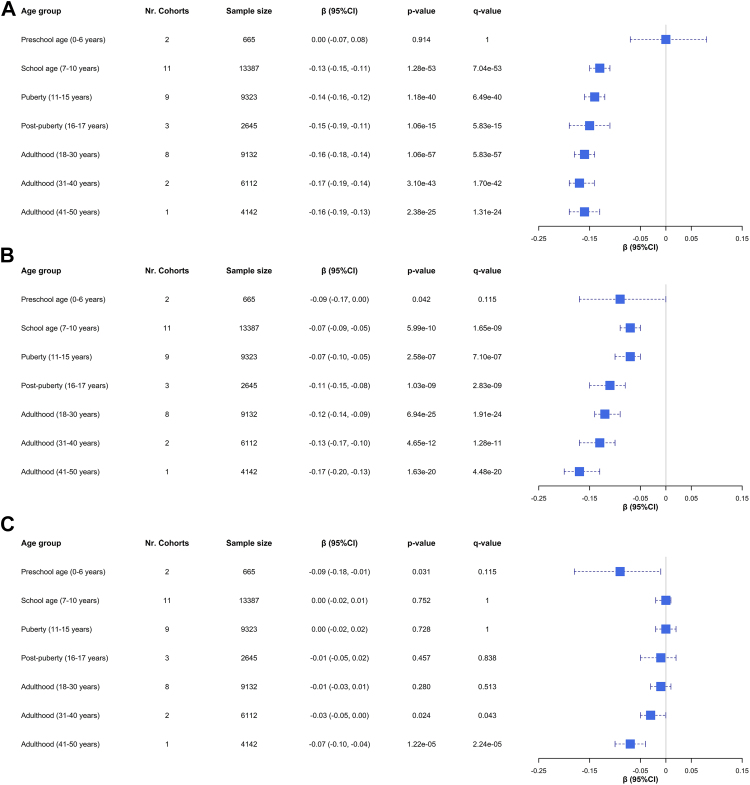
**Forest plot of the effect size of the association between the PRS for airflow limitation and spirometry measurements from preschool age to 50-year-old adulthood.** Blue boxes show the association effects in terms of β estimates after meta-analyzing the results from the cohorts included in each age group. The corresponding 95% Confidence Intervals (95% CI) are represented by blue dash lines. The number of cohorts, sample size, effect size, and *p*-value of the association are also indicated per age group. The *q*-value represents the adjusted *p*-value accounting for the false discovery rate. The results shown for the adulthood group including subjects aged between 41 and 50 years correspond to the association results obtained only in HUNT given the absence of more cohorts with available spirometry data within that age range. Results for the age-stratified meta-analysis (random-effects model) are independently shown for each spirometry measurement in terms of z-scores: A) FEV_1_/FVC; B) FEV_1_; C) FVC. FEV_1_: forced expiratory volume in 1 s; FVC: forced vital capacity; PRS: polygenic risk score.

**Supplementary Figure S1:**
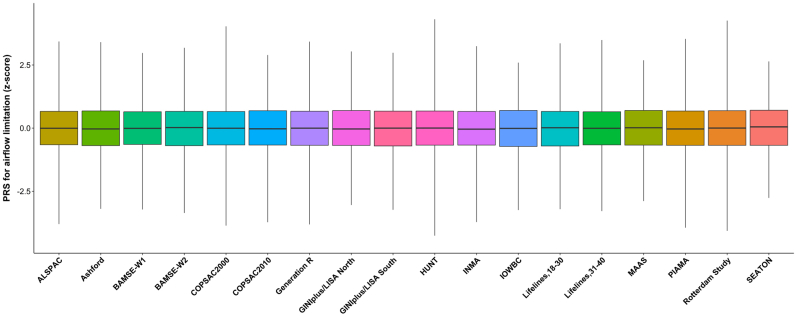


**Supplementary Figure S3:**
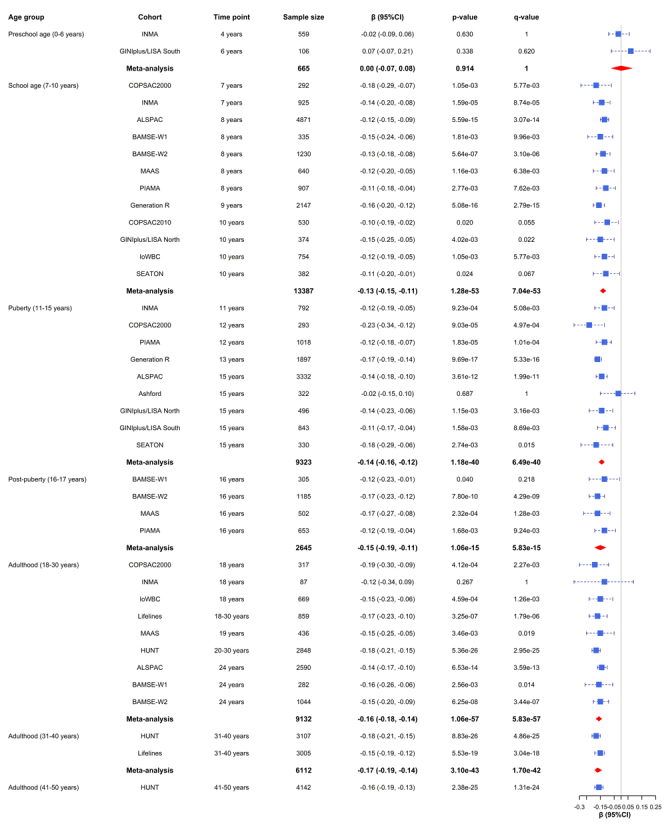


Among the initially selected 82 SNPs associated with COPD (*p*-value ≤5 × 10^−8^),^12^ 77–80 SNPs were included in the PRS calculation across participating cohorts after QC procedures, except for PIAMA (69 SNPs) (Tables S4–S7, Figures S1–S2, Supplementary Results). The PRS for airflow limitation was tested in association with spirometry measurements in each cohort and combined in an age-stratified meta-analysis. A total of 45,406 subjects were included considering the totality of age groups explored and the fact that spirometry was assessed more than once in most studies; thus, some individuals were counted several times across age groups (Table S3). Fig. 2 and Table 2 show the association results with FEV_1_/FVC, FEV_1,_ and FVC z-scores (see Table S8 for cohort-specific results). Overall, stronger associations with FEV_1_/FVC than with other spirometry measurements were observed (Fig. 2A). Evidence of association was detected from school age (β: −0.13 z-scores per one z-score increase in the PRS, 95% Confidence Interval (CI): −0.15, −0.11, *q*-value = 7.04 × 10^−53^) to the oldest adulthood group (41–50 years) (β: −0.16, 95% CI: −0.19, −0.13, *q*-value = 1.31 × 10^−24^) (Table 2, Fig. 2A, Figure S3). Even though the association effect became slightly stronger in the negative direction with age (from school age to adulthood, 41–50 years), no significant relationship between the effect size of the association with FEV_1_/FVC and the age group was observed (*p* = 0.176). Subjects at the top deciles of the distribution of PRS for airflow limitation showed between approximately five and eight times lower mean lung function levels compared to individuals with a lower genetic risk for COPD. This is exemplified in one pediatric cohort, where a mean FEV_1_/FVC z-score of −0.62 in adults aged 18–30 years from the top decile was observed to be significantly different from the mean of −0.11 in subjects from the bottom decile of the PRS (β: −0.52, 95% CI: −0.75, −0.28, *p*-value = 2.35 × 10^−5^, *q*-value = 6.88 × 10^−4^) (Figure S4A, Figure S5C). This change was also found in individuals from the same age group participating in an adult cohort with even larger effects (β: −0.67, 95% CI: −0.82, −0.52, *p*-value = 3.59 × 10^−19^, *q*-value = 1.05 × 10^−17^) (Figure S4B, Figure S5D).

**Table 2:** Results of the meta-analysis of association estimates of the PRS for airflow limitation with spirometry measurements by age group (up to 50 years).

Age group	Sample size^a^	Nr. Cohorts^b^	Spirometry measurement^c^	β (95% CI)^d^	*p*-value^e^	*q*-value^f^	*I*^*2*^^g^	*p*-value_Q_^h^
Preschool age (0–6 years)	665	2	FEV_1_	−0.09 (−0.17, 0.00)	0.042	0.115	0	0.606
			FVC	−0.09 (−0.18, −0.01)	0.031	0.115	0	0.436
			FEV_1_/FVC	0.00 (−0.07, 0.08)	0.914	1	13.63	0.282
School age (7–10 years)	13,387	11	FEV_1_	−0.07 (−0.09, −0.05)	5.99 x 10^−10^	**1.65 x 10**^**−**^**^9^**	51.79	0.007
			FVC	0.00 (−0.02, 0.01)	0.752	1	0.80	0.220
			FEV_1_/FVC	−0.13 (−0.15, −0.11)	1.28 x 10^−53^	**7.04 x 10**^**−**^**^53^**	0	0.932
Puberty (11–15 years)	9323	9	FEV_1_	−0.07 (−0.10, −0.05)	2.58 x 10^−7^	**7.10 x 10**^**−**^**^7^**	36.29	0.092
			FVC	0.00 (−0.02, 0.02)	0.728	1	0	0.435
			FEV_1_/FVC	−0.14 (−0.16, −0.12)	1.18 x 10^−40^	**6.49 x 10**^**−**^**^40^**	1.81	0.280
Post-puberty (16–17 years)	2645	3	FEV_1_	−0.11 (−0.15, −0.08)	1.03 x 10^−9^	**2.83 x 10**^**−**^**^9^**	0	0.575
			FVC	−0.01 (−0.05, 0.02)	0.457	0.838	0	0.857
			FEV_1_/FVC	−0.15 (−0.19, −0.11)	1.06 x 10^−15^	**5.83 x 10**^**−**^**^15^**	0	0.550
Adulthood (18–30 years)	9132	8	FEV_1_	−0.12 (−0.14, −0.09)	6.94 x 10^−25^	**1.91 x 10**^**−**^**^24^**	13	0.520
			FVC	−0.01 (−0.03, 0.01)	0.280	0.513	0	0.715
			FEV_1_/FVC	−0.16 (−0.18, −0.14)	1.06 x 10^−57^	**5.83 x 10**^**−**^**^57^**	2	0.908
Adulthood (31–40 years)	6112	2	FEV_1_	−0.13 (−0.17, −0.10)	4.65 x 10^−12^	**1.28 x 10**^**−**^**^11^**	54.48	0.138
			FVC	−0.03 (−0.05, 0.00)	0.024	**0.043**	0	0.622
			FEV_1_/FVC	−0.17 (−0.19, −0.14)	3.10 x 10^−43^	**1.71 x 10**^**−**^**^42^**	0.75	0.315
Adulthood (41–50 years)^i^	4142	1	FEV_1_	−0.17 (−0.20, −0.13)	1.63 x 10^−20^	**4.48 x 10**^**−**^**^20^**	NA	NA
			FVC	−0.07 (−0.10, −0.04)	1.22 x 10^−5^	**2.24 x 10**^**−**^**^5^**	NA	NA
			FEV_1_/FVC	−0.16 (−0.19, −0.13)	2.38 x 10^−25^	**1.31 x 10**^**−**^**^24^**	NA	NA

CI: confidence interval; FEV_1_: forced expiratory volume in 1 s; FVC: forced vital capacity.

Results are shown for the basic association model, including Principal Components of genetic ancestry and any cohort-specific variables as covariates.

Significant results (*q*-value ≤0.05) are highlighted in bold font.

a Number of individuals with available genotype data and spirometry measurements included in the association analyses.

b Number of cohorts included in the meta-analysis per age group.

c Z-score of pre-bronchodilator spirometry measurements obtained from applying the Global Lung Function Initiative equations.

d Effect size as the change in z-score of lung function per one z-score increase in the PRS.

e A random-effects model was applied to account for the heterogeneity across studies regardless of the significance of the Cochran Q-test and *I*^*2*^ estimate.

f Adjusted *p*-value accounting for the false discovery rate. The Benjamini & Yekutieli method was applied across spirometry measurements per age group.

g Percentage of variation across cohorts due to heterogeneity.

h P-value of the Cochran Q-test of heterogeneity.

i Results shown correspond only to the association testing with spirometry measurements in the HUNT cohort given the absence of more cohorts with available spirometry data within that age range.

**Supplementary Figure S6:**
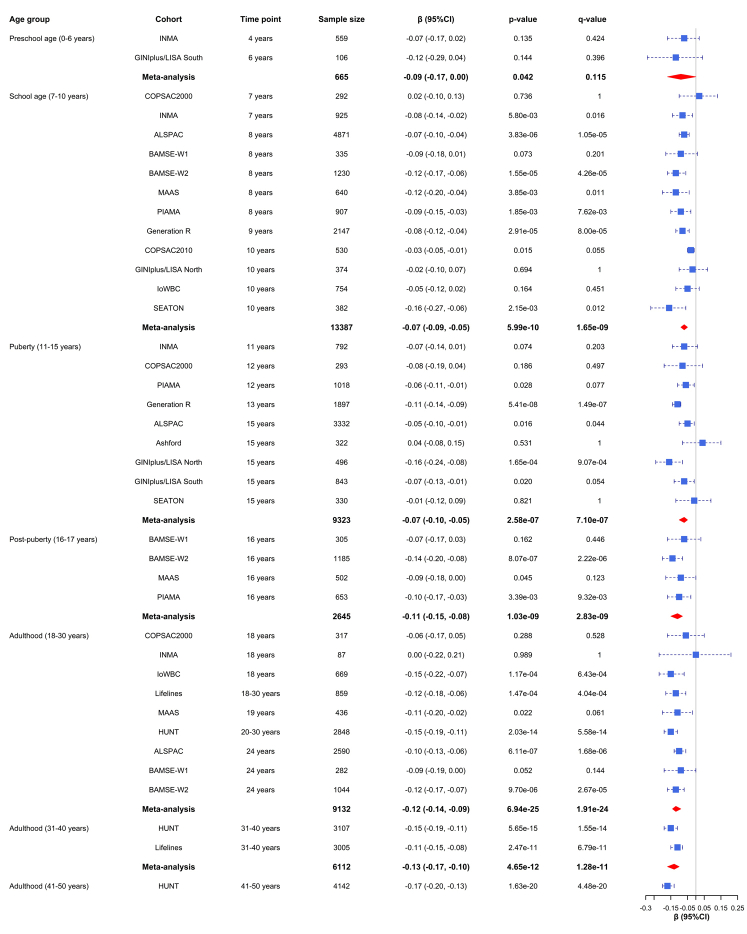


**Supplementary Figure S7:**
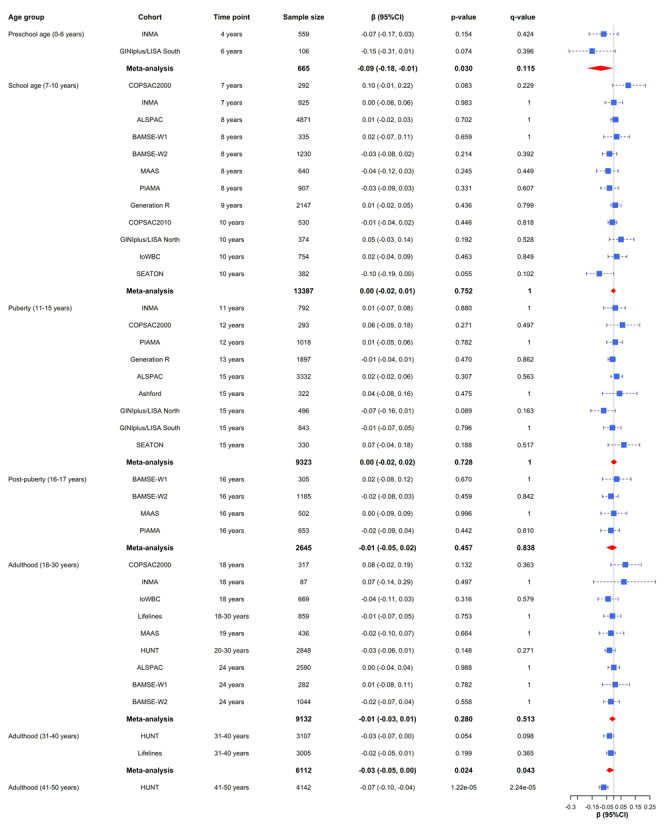


Analogously, similar association effects with z-scores of FEV_1_ were found across the age groups (Fig. 2B, Figure S6). The magnitude of the association with lower FEV_1_ increased to some extent in older groups, but only a nominally significant trend in the association effect size across age groups was found (*p* = 0.016). Association with FVC was detected in adults aged between 31 and 40 years old, and also those included in the 41-50-year age group (Fig. 2C, Figure S7). No significant relationship between the effect size of the association with FVC and the age groups was detected (*p* = 0.098). The effect size and significance level of the association with FVC were found to be weaker than with FEV_1_ and FEV_1_/FVC within each of these age groups (Table 2). Additionally, evidence of nominal association with FEV_1_ (β: −0.09, 95% CI: −0.17, 0.00, *p*-value = 0.042) and FVC (β: −0.09, 95% CI: −0.18, −0.01, *p*-value = 0.031) was observed in preschool children. This age group makes an exception to the slightly increased effect size with lower spirometry measurements with age, where a stronger association effect size with FEV_1_ and FVC than expected was found compared to the subsequent groups. However, these associations did not remain significant after FDR adjustment (*q*-value = 0.115) (Table 2, Fig. 2B and C, Figures S6 and S7).

The association results with FEV_1_ and FEV_1_/FVC z-scores were validated in 9,027 subjects aged >50 years old, with a similar association effect size (Table 3). Similarly, lower FEV_1_/FVC levels were observed in participants of this age group at the highest decile of the PRS distribution compared to those with the lowest genetic risk (e.g., a mean FEV_1_/FVC z-score of −0.91 in the top decile *versus* −0.26 in the bottom decile of the PRS distribution) (Figure S4B). This change was found to be statistically significant (β: −0.64, 95% CI: −0.75, −0.53, *p*-value = 8.20 × 10^−29^, *q*-value = 2.40 × 10^−27^) (Figure S5G). In this older dataset, a significant association with z-scores of FVC was also detected (β: −0.07, 95% CI: −0.09, −0.05, *q*-value = 1.06 × 10^−9^) (Table 3). Similar results were observed in participants older than 50 years from the three time points evaluated in an independent cohort included in the base dataset (Table S10).

**Table 3:** Association results with spirometry measurements in HUNT participants aged >50 years from the basic regression model and sensitivity analyses accounting for smoking.

Association model	Sample size^a^	Spirometry measurement^b^	β (95% CI)^c^	*p*-value	*q*-value^d^
Basic^e^	9027	FEV_1_	−0.16 (−0.19, −0.13)	5.80 x 10^−31^	**1.60 x 10**^**−**^**^30^**
		FVC	−0.07 (−0.09, −0.05)	5.77 x 10^−10^	**1.06 x 10**^**−**^**^9^**
		FEV_1_/FVC	−0.17 (−0.19, −0.14)	1.97 x 10^−39^	**1.09 x 10**^**−**^**^38^**
Sensitivity–Smoking status^f^	8784^h^	FEV_1_	−0.16 (−0.19, −0.13)	5.70 x 10^−31^	**1.57 x 10**^**−**^**^30^**
		FVC	−0.07 (−0.09, −0.05)	9.77 x 10^−10^	**1.79 x 10**^**−**^**^9^**
		FEV_1_/FVC	−0.17 (−0.19, −0.14)	3.79 x 10^−40^	**2.08 x 10**^**−**^**^39^**
Sensitivity–Smoking pack-years^g^	2205^i^	FEV_1_	−0.19 (−0.24, −0.14)	7.08 x 10^−13^	**1.95 x 10**^**−**^**^12^**
		FVC	−0.08 (−0.13, −0.04)	2.46 x 10^−4^	**4.52 x 10**^**−**^**^4^**
		FEV_1_/FVC	−0.21 (−0.26, −0.16)	3.00 x 10^−16^	**1.65 x 10**^**−**^**^15^**

CI: confidence interval; FEV_1_: forced expiratory volume in 1 s; FVC: forced vital capacity; PC: Principal Component of genetic ancestry.

Significant results (*q*-value ≤0.05) are highlighted in bold font.

a Number of individuals with available genotype data and spirometry measurements included in the association analyses.

b Z-score of pre-bronchodilator spirometry measurements obtained from applying the Global Lung Function Initiative equations.

c Effect size as the change in z-score of lung function per one z-score increase in the PRS.

d Adjusted *p*-value accounting for the false discovery rate. The Benjamini & Yekutieli method was applied across spirometry measurements per age group.

e Basic association model, including ten PCs, the participation round, and the genotyping batch as covariates.

f Sensitivity analyses accounting for active smoking. Ten PCs, the participation round, the genotyping batch, and smoking status were included as covariates.

g Sensitivity analyses accounting for active smoking. Ten PCs, the participation round, the genotyping batch, and smoking pack-years were included as covariates. Tobacco pack-years were calculated by multiplying the number of smoking years by the number of daily cigarettes and dividing by 20 cigarettes often contained in a package.

h Number of individuals with available genotype data, spirometry measurements, and smoking status information included in the association analyses.

i Number of individuals with reported active smoking habits and available genotype data, spirometry measurements, smoking status information included in the association analyses.

**Fig. 3:**
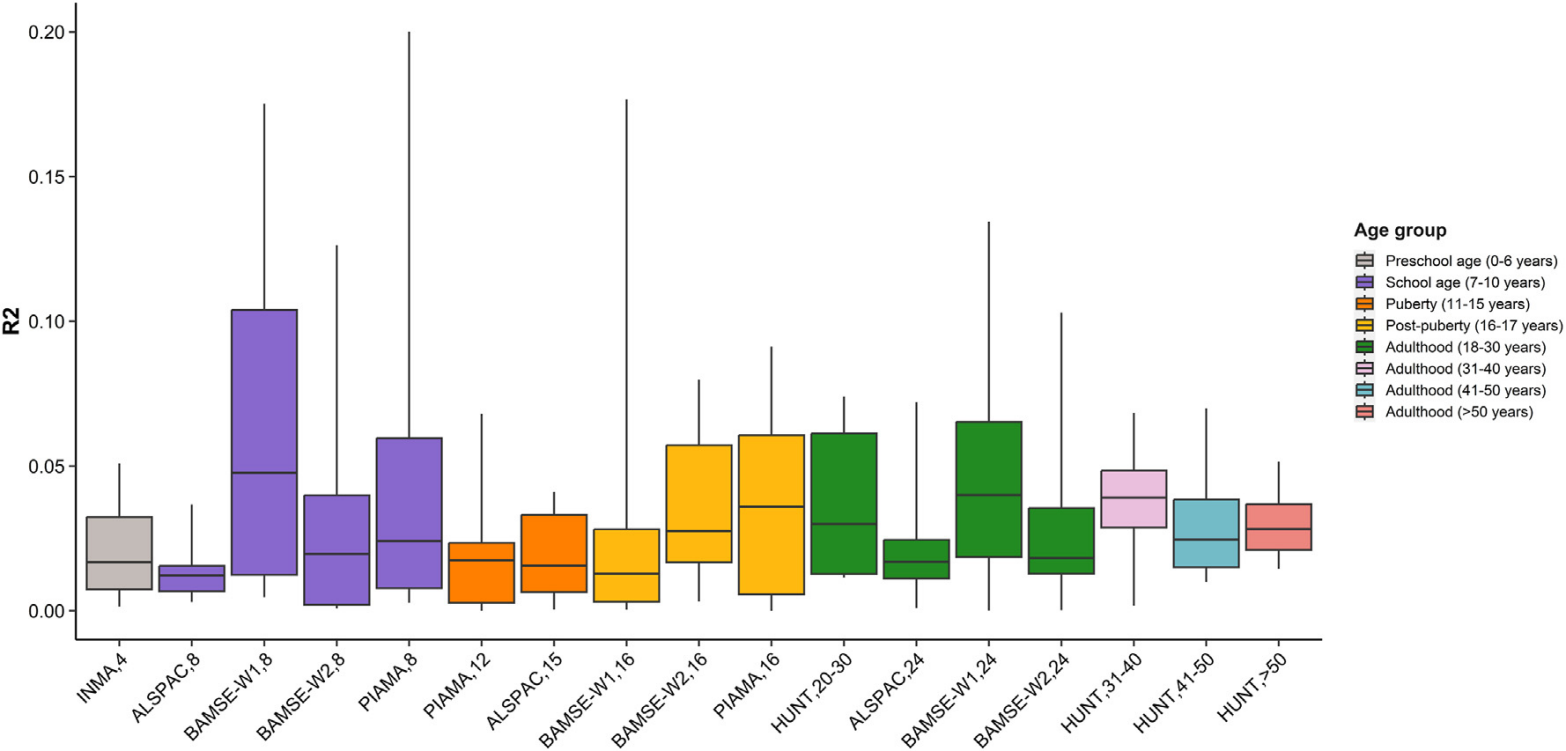
**Box plot of variance in FEV**_**1**_**/FVC explained by the PRS for airflow limitation across the lifespan.** The proportion of the total variance in FEV_1_/FVC explained by the PRS is shown in the *y*-axis in terms of R2. The time points from large cohorts from each age group are represented in the *x*-axis. Boxes are color-coded based on the age group from preschool age to adulthood (>50 years). The median of the R2 is displayed by the thick horizontal line at each box, whereas whiskers extending vertically indicate the minimum and maximum values. The variance explained by the PRS for airflow limitation was estimated including the same sample size and covariates of the basic association model (Principal Components of genetic ancestry and any cohort-specific covariates). FEV_1_: forced expiratory volume in 1 s; FVC: forced vital capacity; PRS: polygenic risk score; W1: Genotyping Wave 1; W2: Genotyping Wave 2.

The PRS for airflow limitation explained a limited proportion of the total variance in lung function indices (Table S11). The largest proportion of the variance explained by the PRS was found for the z-score of FEV_1_/FVC, with an average ranging from 1.5% to 6.5% across age groups (Fig. 3).

### Assessment of the effect of additional risk factors

#### Active smoking habits

**Supplementary Figure S9:**
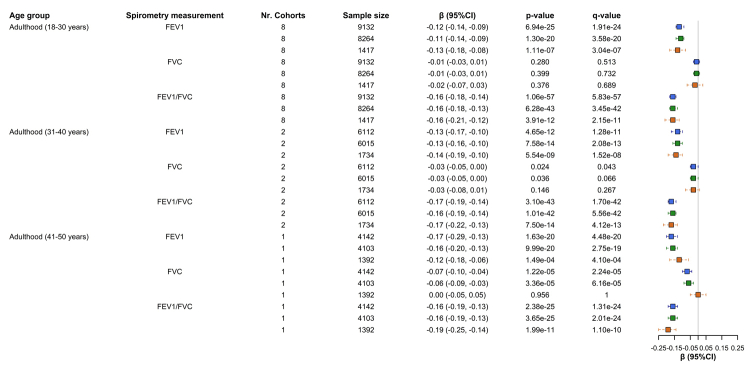


No major changes in terms of the magnitude of the association effect or significance level were detected in adults aged up to 50 years when further adjusting by a covariate related to active smoking status in the regression models and performing a meta-analysis in the adulthood age groups (Table 4, Figure S9). However, the association with FVC z-scores observed in adults aged 31–40 years (Table 2) was not significant when accounting for this variable and correcting for multiple comparisons. Findings were consistent in adults older than 50 years (Table 3, Table S10). Even though no substantial differences in the effect size were detected accounting for tobacco pack-years, the association with FVC z-scores in the 31–40 and 41-50-year age groups (Table 2) did not remain after adjusting by this covariate (Table 4, Figure S9). This association remained significant in older adults (>50 years of age) (Table 3), except for Rotterdam Study PFT 1 (50–98 years) and PFT 2 (51–96 years), although with almost identical effect sizes. Surprisingly, the effect size of the association with FEV_1_, FVC, and FEV_1_/FVC increased in PFT 3 (70–100 years) when this marker of cumulative smoking history was also included as a covariate (Table S10). These differences might be explained by the reduced sample size of subjects with reported smoking habits and available information related to pack-years at this time point (n = 45).

**Table 4:** Results of the meta-analyses accounting for active smoking habits in adults aged 18–50 years.

Age group	Spirometry measurement^c^	Smoking status^a^				Smoking pack-years^b^			
		Sample size^d^	β (95% CI)^e^	*p*-value^f^	*q*-value^g^	Sample size^h^	β (95% CI)^e^	*p*-value^f^	*q*-value^g^
Adulthood (18–30 years)	FEV_1_	8264	−0.11 (−0.14, −0.09)	1.30 x 10^−20^	**3.58 x 10**^**−**^**^20^**	1417	−0.13 (−0.18, −0.08)	1.11 x 10^−7^	**3.05 x 10**^**−**^**^7^**
	FVC	8264	−0.01 (−0.03, 0.01)	0.399	1	1417	−0.02 (−0.07, 0.03)	0.376	0.689
	FEV_1_/FVC	8264	−0.16 (−0.18, −0.13)	6.28 x 10^−43^	**3.45 x 10**^**−**^**^42^**	1417	−0.16 (−0.21, −0.12)	3.91 x 10^−12^	**2.15 x 10**^**−**^**^11^**
Adulthood (31–40 years)	FEV_1_	6015	−0.13 (−0.16, −0.10)	7.58 x 10^−14^	**2.08 x 10**^**−**^**^13^**	1734	−0.14 (−0.19, −0.10)	5.54 x 10^−9^	**1.52 x 10**^**−**^**^8^**
	FVC	6015	−0.03 (−0.05, 0.00)	0.036	0.066	1734	−0.03 (−0.08, 0.01)	0.146	0.268
	FEV_1_/FVC	6015	−0.16 (−0.19, −0.14)	1.01 x 10^−42^	**5.56 x 10**^**−**^**^42^**	1734	−0.17 (−0.22, −0.13)	7.50 x 10^−14^	**4.13 x 10**^**−**^**^13^**
Adulthood (41–50 years)^i^	FEV_1_	4103	−0.16 (−0.20, −0.13)	9.99 x 10^−20^	**2.75 x 10**^**−**^**^19^**	1392	−0.12 (−0.18, −0.06)	1.49 x 10^−4^	**4.10 x 10**^**−**^**^4^**
	FVC	4103	−0.06 (−0.09, −0.03)	3.36 x 10^−5^	**6.16 x 10**^**−**^**^5^**	1392	0.00 (−0.05, 0.05)	0.956	1
	FEV_1_/FVC	4103	−0.16 (−0.19, −0.13)	3.65 x 10^−25^	**2.01 x 10**^**−**^**^24^**	1392	−0.19 (−0.25, −0.14)	1.99 x 10^−11^	**1.09 x 10**^**−**^**^10^**

CI: confidence interval; FEV_1_: forced expiratory volume in 1 s; FVC: forced vital capacity.

Results are shown for sensitivity analyses exploring the potential modification of the association effect by active smoking in adults (≥18 years of age).

Significant results (*q*-value ≤0.05) are highlighted in bold font.

a Linear regressions were adjusted by the same covariates as in the basic association model, including PCs of genetic ancestry and any cohort-specific variables as covariates and additionally smoking status.

b A covariate related to tobacco pack-years was added to the basic regression model. Tobacco pack-years were calculated by multiplying the number of smoking years by the number of daily cigarettes and divided by 20 cigarettes often contained in a package.

c Z-score of pre-bronchodilator spirometry measurements obtained from applying the Global Lung Function Initiative equations.

d Number of individuals with available genotype data, spirometry measurements, and smoking status information included in the association analyses.

e Effect size of the association as the change in z-score of lung function per one z-score increase in the PRS.

f A random-effects model was applied to account for the heterogeneity across studies regardless of the significance of the Cochran Q-test and *I*^*2*^ estimate.

g Adjusted *p*-value accounting for the false discovery rate. The Benjamini & Yekutieli method was applied across spirometry measurements per age group.

h Number of individuals with reported active smoking habits and available genotype data, spirometry measurements, and smoking status information included in the association analyses.

i Results shown correspond only to the association testing with spirometry measurements in the HUNT cohort given the absence of more cohorts with available spirometry data within that age range.

#### Sex and asthma

**Supplementary Figure S10:**
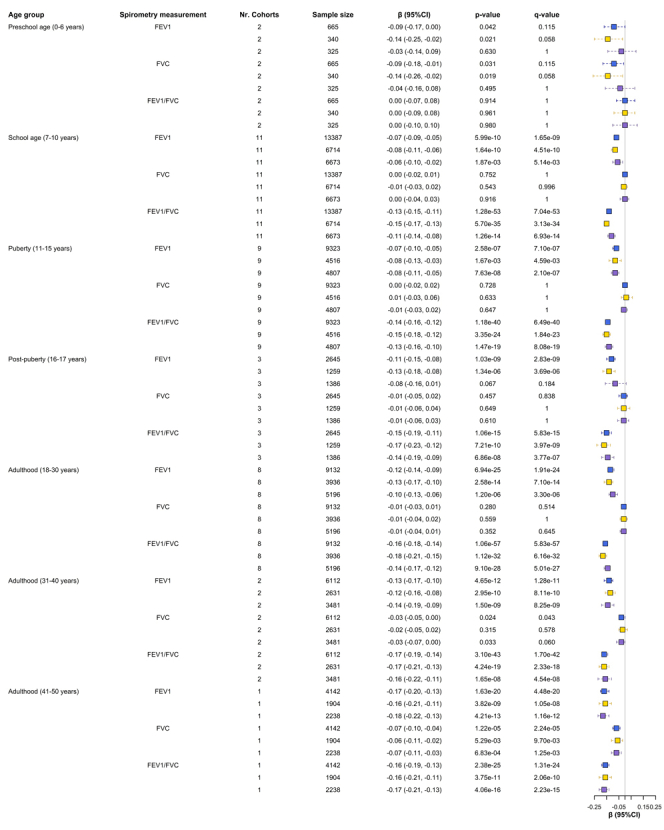


A similar effect size of the association with lower FEV_1_ and FEV_1_/FVC z-scores from school age to late adulthood was observed after stratifying the analyses by sex (Table S14, Figure S10). Similar findings were obtained in adults aged >50 years (Tables S15 and S16). Additionally, effect estimates of the association between the PRS for airflow limitation and lung function were very similar to the original results of the basic association model after excluding subjects with a report of asthma in one of the cohorts (Table S17). No evidence of a significant association of the PRS for airflow limitation with asthma was observed either (Table S18).

Further details of the results obtained in this study can be found in the Supplementary Material.

## Discussion

Our results provide firm evidence that genetic variants linked to an increased risk for COPD (i.e., moderate-to-severe airflow limitation) are also associated with lower lung function across the life course from childhood to adulthood. The association effect sizes remained similar when accounting for smoking habits or after stratifying by sex and were validated in subjects aged above 50 years from two independent cohorts. Collectively, our results strongly support that COPD should be considered a disease whose pathophysiologic mechanisms operate across the entire life course.^9^

Although our understanding of the influence of the genetic makeup of COPD on lung function across the lifespan is still incomplete,^9^ our results support the involvement of loci for airflow limitation in lower FEV_1_/FVC and FEV_1_ not only in adults but also in childhood and adolescence. Nonetheless, firm evidence of the association in preschool children could not be provided, which might be explained by the technical challenges of spirometry testing in young children^27^ and the smaller sample size of this age group. Therefore, the association results obtained in this age group should be taken with caution. The role of genetic variants combined into a quantitative PRS related to the development of COPD has been previously investigated mainly in adults, but most PRSs were developed based on association signals of lung function outcomes.^17^^,^^28^, ^29^, ^30^, ^31^, ^32^, ^33^, ^34^, ^35^ The rationale for the use of genetic variants associated with lung function measures for disease prediction in those studies has been the fact that COPD might occur after the progressive deterioration of lung function before the criteria for diagnosis can be met.^17^ For instance, Moll et al.^17^ calculated PRSs for FEV_1_ and FEV_1_/FVC based on a GWAS of each of these spirometry measurements carried out by Shrine et al.,^31^ which were combined into a single score by the weighted sum of both PRSs.^17^ Therefore, the main difference between the PRS built by Moll et al. and the PRS for airflow limitation presented in this study is the base dataset, which was comprised of association signals of quantitative spirometry measurements in the first, and a binary variable of COPD status based on airflow limitation in ours.^12^ Significant associations of PRSs for lung function with lower FEV_1_ and/or FEV_1_/FVC in adults have been described.^28^^,^^30^^,^^33^^,^^35^ However, only a few studies have revealed associations with lower lung function indices in children.^29^ Indeed, the special importance of the combination of high genetic risk for lower lung function with prematurity has been suggested in preschool children.^34^ Nonetheless, scarce investigation on the implication of genetic determinants of COPD on lung function throughout childhood, adolescence, and adulthood had been conducted in individuals born term before the present study.^36^

PRSs for lung function have been linked to a higher risk for the development of COPD in adults from different ancestry groups.^17^^,^^28^, ^29^, ^30^, ^31^^,^^35^ There is a substantial overlap between genetic markers of lung function and COPD, mostly given the key contribution of spirometry measurements to the current criteria for a COPD diagnosis.^10^ Nonetheless, the evaluation of PRS estimates based on association signals of COPD susceptibility in relation to lung function across the life course has been scarcely investigated before the present study.^18^^,^^28^^,^^31^

Several PRSs for lung function, as a proxy of COPD, have been linked to visual and quantitative emphysema and lung structure-related traits revealed by computed tomography (CT) imaging.^17^^,^^30^^,^^32^ It has been suggested that part of the risk for COPD might be due to genetic factors linked to developmental processes^11^ given the genetic overlap between COPD and height,^37^ and regulatory regions of the genome involved in lung development.^38^ Indeed, Sakornsakolpat et al. thoroughly investigated the potential functional implications of the loci associated with airflow limitation included in our PRS and observed the enrichment of epigenomic markers in fetal lung tissue and gene sets in lung morphogenesis and development-related processes.^12^ This supports the hypothesis of the early origins of COPD and our findings in children. The combination of different data layers revealed potential candidate effector genes involved in functions related to the extracellular matrix, previously linked to lung function.^12^ There is previous evidence suggesting that the variation in lung structure might be an important mediator and a plausible underlying biological mechanism,^17^^,^^30^ but the association of the PRS for airflow limitation with CT imaging-related phenotypes could not be explored in the present study given the lack of such data in the participating cohorts. However, Sakornsakolpat et al. identified gene clusters associated with emphysema features (e.g., *CHRM3*, *ITGA1*, *FAM13A*) and airway structure (e.g., *ASTN2*, *AGER*, *ADAMTSL3*) revealed by CT scan through a phenome-wide association analysis.^12^ These results suggest the implication of our PRS for airflow limitation not only in spirometry measurements across the lifespan but also in lung structure and emphysema features, at least in adulthood.

Tobacco smoking has been classically considered the most important environmental risk factor for decreased lung function and COPD development.^6^ Nonetheless, we did not find any smoking-related variables to modify the association between genetic determinants for airflow limitation and lung function in adulthood, suggesting that the loci included in our PRS exert an effect on lung function independent of smoking. The GWAS that served as the base dataset for our study precisely aimed to identify pure genetic effects on COPD susceptibility independent from environmental influences. Their findings were certainly demonstrated not to be driven by any smoking-related variable,^12^ which is consistent with previous suggestions of shared genetic determinants for airflow limitation between heavy smokers and non-smokers.^31^ Additionally, strong interactions between genetic factors and smoking have been scarcely reported to date.^11^^,^^18^^,^^39^ Altogether, these pieces of evidence suggest that smoking and the individual's genetic composition might exert separate effects on the risk for the development of COPD.

Females often show a faster decline in lung function, more severe COPD, higher risk for exacerbations and development of early-onset COPD, as well as more comorbidities and different clinical manifestations.^40^ The mechanisms underlying these differences by sex are still unknown, although genetic factors have been proposed to be fundamental contributors.^41^ Our study did not reveal any substantial effect of sex in the association of the PRS for airflow limitation with lung function at any of the age groups evaluated, concordantly with the design of the base dataset aiming at the identification of sex-independent COPD association signals.^12^

The proportion of the total variation in spirometry measurements explained by the PRS for airflow limitation developed in the present study (up to 6%) might seem small compared to the moderate predictability of COPD risk revealed by previous PRSs for COPD or lung function in addition to clinical factors (area under the curve (AUC)_Europeans_ = 0.60–0.80; AUC_African Americans_ = 0.75; AUC_East Asians_ = 0.79).^17^^,^^28^^,^^31^ It is worth noting that this increases when CT-related phenotypes are also taken into account.^30^ However, these estimates are not comparable to the ones obtained in our study since our ultimate objective was not to develop a predictive tool for COPD risk but rather to understand the contribution of genetic factors of COPD on lung function across different age groups from childhood to adulthood. Furthermore, the outcome under investigation has been spirometry measurements instead of COPD status; thus, weaker effect sizes are to be expected in this scenario. Nonetheless, the estimates of the proportion of variance explained by our PRS are consistent with other complex traits, such as body mass index (2.4–9.5%)^42^^,^^43^ and blood pressure (0.5–7.5%).^44^

We also attempted to assess whether there were differences in the magnitude of the effect from childhood to adulthood but found no strong evidence of a trend in the association effect estimates across age groups. Future efforts including a large range of environmental exposures and combination with other omics layers could help us to shorten the path to achieve the early and accurate prediction and identification of subjects at high risk of COPD. The success of these approaches in other traits^45^, ^46^, ^47^, ^48^, ^49^ suggests their potential, even though these are still incipient in the respiratory field.^49^, ^50^, ^51^ The substantial heterogeneity of COPD is an important aspect that should be also taken into account since it might be the result of the involvement of trajectories of poor lung function marked by reduced lung growth, an early and reduced plateau phase, or an accelerated decline.^17^ Indeed, the latter is one of the key events leading to the development of COPD.^52^ Even though a rapid decline in lung function has been suggested to be a result of the interaction of several factors,^53^ the underlying molecular and cellular mechanisms are yet to be disentangled. This has been evidenced to be a heritable trait^54^ but, only a limited number of genetic loci have been identified to date; thus, the calculation of a PRS of this trait still seems unfeasible. The strong association of genetic markers of airflow limitation with lower FEV_1_/FVC z-scores we found since early life suggests that the mechanisms that lead to the development of COPD for some adults already begin in childhood. A potential direct clinical use of an individual PRS to assess COPD risk (and general lung health) remains to be evaluated, although early screening of lung function in subjects with a high genetic risk for COPD has been discussed.^55^

The most important strength of our work is the evaluation of a PRS for adult airflow limitation in 16 different, large cohorts from childhood to adulthood. This allowed us to investigate the influence of a genetic risk for airflow limitation on lung function levels in different age groups across the life course, including young subjects primarily from longitudinal cohorts. Most of the previous work had been carried out on adults, with a clear lack of evaluation of how genetic determinants for COPD might affect lung function across the life course. We acknowledge that this study has several limitations. First, COPD was defined based on only pre-bronchodilator spirometry information in the base dataset^12^; thus, the genetic variants identified might be signals of solely airflow limitation rather than COPD in its complete sense. The criteria utilized for the definition of COPD cases were based on FEV_1_/FVC and FEV_1_,^6^ which might explain the fact that the strongest evidence of association of our PRS was observed with FEV_1_/FVC, followed by FEV_1_, and the absence of significant association with FVC in most younger age groups. Additionally, pre-bronchodilator spirometry measurements were used^12^ despite international recommendations for the definition of COPD based on measures obtained after the administration of bronchodilators to minimize potential variability.^6^ Second, most cohorts included in the present work were of European ancestry, except for a small proportion of subjects of several non-European ethnic groups from one participating cohort. This limited us from drawing firm conclusions applicable to other ancestry groups. However, the calculation of our PRS in non-Europeans might result in expected reduced predictability given the ancestry heterogeneity with the base dataset. Furthermore, the use of summary statistics of a multi-ancestry GWAS might increase the predictive power and applicability of PRSs estimated in different populations,^35^ although these studies are still widely underrepresented.^56^ Nonetheless, the future validation of the association of our PRS with lung function indices across the life course in diverse ancestry groups would be of great interest. Third, the association of the PRS for airflow limitation with spirometry measurements was evaluated in a different number of available time points among the participating cohorts. Additionally, both longitudinal and cross-sectional cohorts were included in this study. Fourth, a reduced set of genetic variants restricted to those reaching the genome-wide significance level was included in the calculation of our PRS for airflow limitation, which might seem conservative and simplistic with consequences on the proportion of variance explained.^23^ However, Sakornsakolpat et al. demonstrated the robustness of the selected association signals by the combination of different omics layers, and the association with CT features, and comorbidities.^12^ Despite the contradictory evidence of the influence of the number of SNPs on the PRS performance,^57^^,^^58^ it has been suggested that it highly depends on the intended application of a given PRS.^59^ Risk prediction often requires a large set of genetic variants,^23^ whereas the selection of independent signals with strong evidence of association might be the most appropriate approach for the evaluation of the effect of an exposure on an outcome (as in our study).^56^^,^^59^ Furthermore, most of the loci included in the PRS for airflow limitation described here had also been previously associated with lung function measurements,^29^^,^^60^, ^61^, ^62^ which might not reflect only COPD susceptibility *per se* but also lung function. Finally, asthma and COPD diagnoses were not taken into account in the basic association testing, although COPD is rarely diagnosed in individuals younger than 50 years and never in children. However, sensitivity analyses in relation to asthma showed reassuring results.

This study provides fundamental evidence of the link between the genetics of airflow limitation linked to COPD and lung function, primarily FEV_1_/FVC, across the life course independent of tobacco smoking. These findings suggest that a higher genetic risk of developing COPD in combination with several other factors is linked to lower lung function from an early age. This has important implications for COPD prevention as early in life as possible.

## Contributors

NH-P, AKi, AKu, JAC, NO, SKr, XB, LLa, LB, RG, SM, JRB, YS, C-ETP, TK, RF, AA, MS, and EM equally contributed to this work. NH-P, AKu, SKM, GW, JH, and EM were involved in the conceptualization of this study. NH-P, AKi, JAC, NO, SKr, XB, LLa, LB, RG, SM, JRB, YS, C-ETP, TK, ET, and CD conducted the formal analyses. LLa, SKo, JG-A, AE, MT, JI, LLo, AS, UG, RCHV, GR, AB, JMV, JFF, LD, KB, NT, GB, BMB, AL, ST, JWH, SHA, AC, PC, CSM, MVDB, IK, TS, JAW, GK, RF, AA, MS, and EM participated in the funding acquisition for the included cohorts or this specific project. NH-P, AKi, AKu, JAC, NO, SKr, XB, LLa, LB, RG, SM, JRB, YS, C-ETP, TK, ET, CD, SKB, GW, JH, and EM were involved in the investigation. NH-P, AKi, AKu, SKM, GW, JH, MS, and EM participated in designing the methodology used in this study. NH-P and EM were responsible for the project administration and coordination. LLa, JH, SKo, JG-A, AE, MT, JI, LLo, AS, UG, RCHV, GR, AB, JMV, JFF, LD, KB, NT, GB, BMB, AL, ST, JWH, SHA, AU, AC, PC, CSM, MVDB, IK, TS, JAW, GK, RF, AA, MS, and EM directly or indirectly provided with the resources necessary for this project. LLa, JRB, JMV, JFF, LD, BMB, TS, GK, RF, AA, MS, and EM supervised the research activity related to this study. NH-P, AKi, JAC, NO, SKr, XB, LLa, LB, RG, SM, JRB, YS, C-ETP, TK, ET, and CD participated in the validation of the findings described. NH-P, JAC, NO, SKr, XB, LLa, LB, RG, SM, JRB, YS, C-ETP, TK, ET, CD, SKB, and GW contributed to the visualization of the results. NH-P, AKu, SKM, GW, JH, and EM wrote the original draft and all authors participated in the revision and editing of this manuscript.

## Data sharing statement

The data supporting the findings of this article can be available from the corresponding authors upon reasonable request.

## Declaration of interests

NH-P was supported with a Medium-Term Research Fellowship by the European Academy of Allergy and Clinical Immunology (EAACI) and a Long-Term Research Fellowship by the European Respiratory Society (ERS) (LTRF202101-00861), and lecture honoraria from OMNIPREX, S.L (outside of the submitted work). LLa was supported by the Fund for Scientific Research Flanders (Grant 3G037618), lecture honoraria from IPSA vzw, a non-profit organization facilitating lifelong learning for health care providers, and Chiesi; and consulting fees from AstraZeneca, all paid to the institution. LLa also declares unpaid membership of faculty board and faculty committees of the European Respiratory Society and Belgian Respiratory Society. LB received support from the K.G. Jebsen Center for Genetic Epidemiology funded by Stiftelsen Kristian Gerhard Jebsen; Faculty of Medicine and Health Sciences, NTNU; The Liaison Committee for Education, Research and Innovation in Central Norway; and the Joint Research Committee between St Olavs Hospital and the Faculty of Medicine and Health Sciences, NTNU. YS was supported by a grant from the China Scholarship Council. AL declares consulting fees regarding presentation of spirometry from Diagnostica Ltb and lecture honoraria from AstraZeneca, Boehringer Ingelheim, and GlaxoSmithKline. AC reports research grants funded by MRC, EPSRC, and Wellcome Trust; consulting fees from Worg Pharmaceuticals; lecture honoraria from GlaxoSmithKline, AstraZeneca, Stallergens-Greer, and Sanofi; and unpaid membership of a board of officers of the World Allergy Organization. GK was supported by grants from ZON-MW, Lung Foundation of the Netherlands, UBBO EMMIUS Foundation, GSK, Vertex, European Union (H2020 program), TEVA the Netherlands; consulting fees from AstraZeneca and PURE IMS; lecture fees from AstraZeneca, Boehringer Ingelheim and Sanofi; and participation as a chair at the exquAIro foundation (AI education for medicine and pharma). RF received funding from the European Research Council (ERC) under the European Union's Horizon 2020 research and innovation program (grant agreement No. 101044387), Instituto de Salud Carlos III (PI18/00018, PI21/00735), SEPAR and Serra Hunter Program. AA reports research grants, consulting fees, and lecture honoraria by GlaxoSmithKline, AstraZeneca, Menarini, Chiesi, and Sanofi; and unpaid roles as Chair Board of Directors of GOLD and Co-chair of CADSET. MS received funding from ERC under the European Union's Horizon 2020 research and innovation program (grant agreement No. 949906). EM is supported by grants from the EU (ERC, TRIBAL No 757919). EM also declares advisory board and lecture fees from ALK, AstraZeneca, and Chiesi outside the submitted work. The rest of the authors declare no conflicts of interest that might be perceived to influence the interpretation of this article.

## Figures and Tables

**Supplementary Figure S2A:**
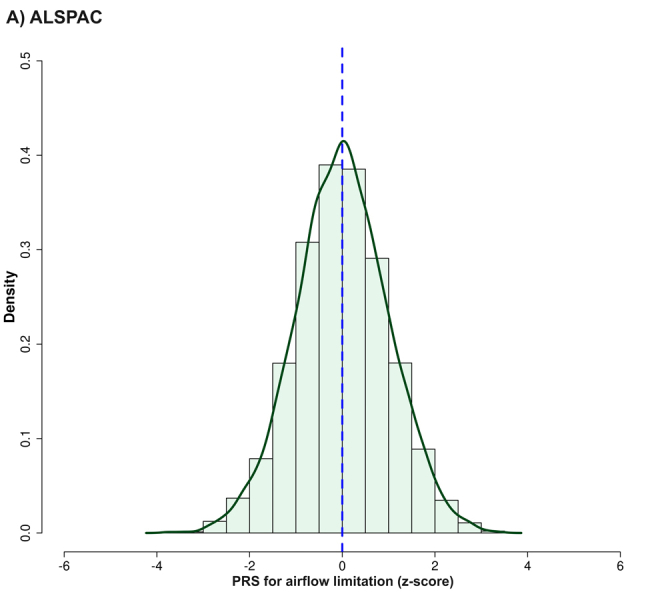


**Supplementary Figure S2B:**
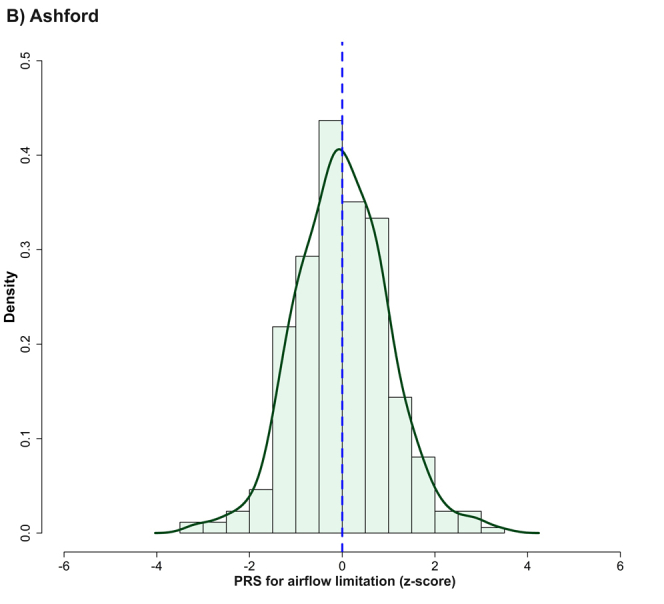


**Supplementary Figure S2C:**
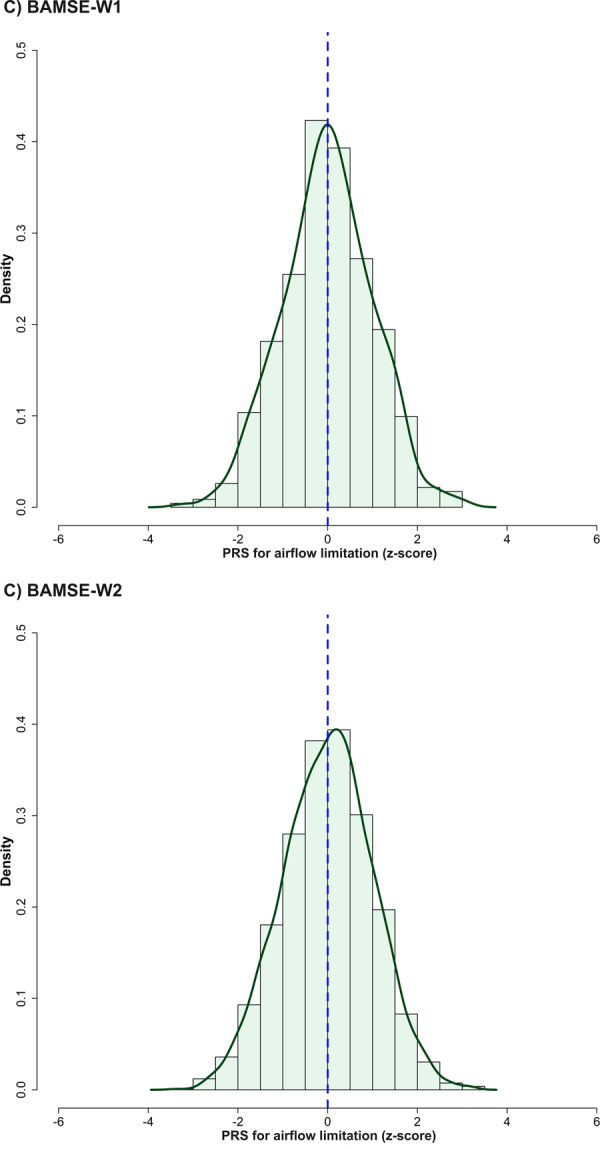


**Supplementary Figure S2D:**
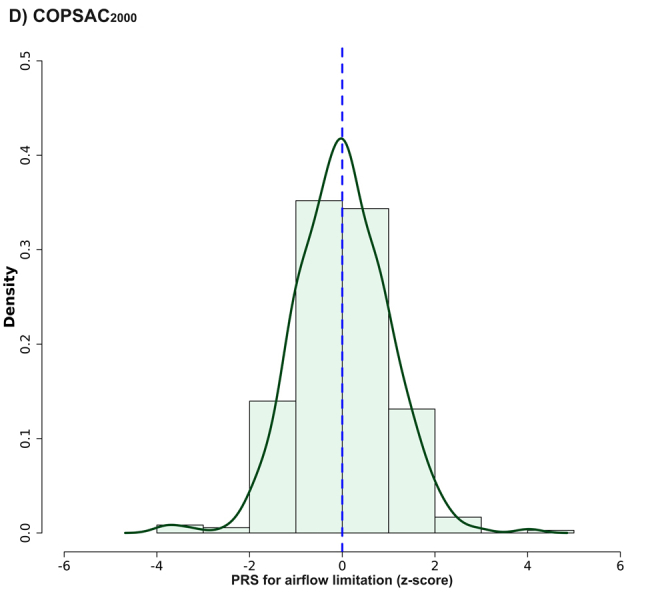


**Supplementary Figure S2E:**
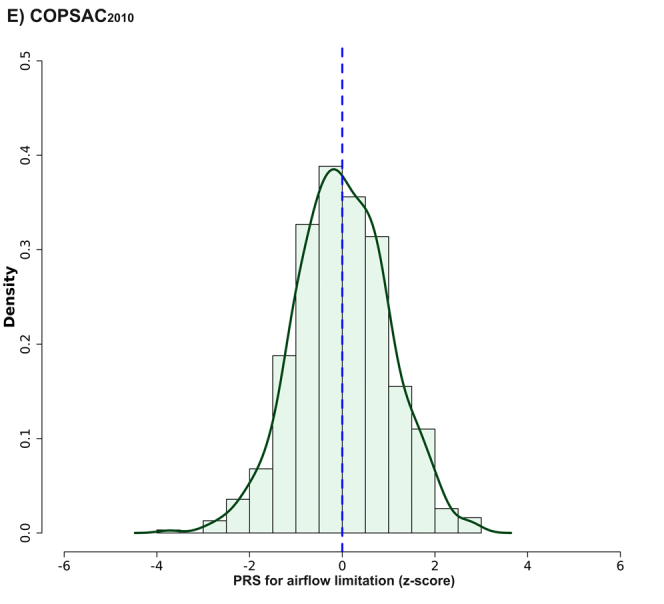


**Supplementary Figure S2F:**
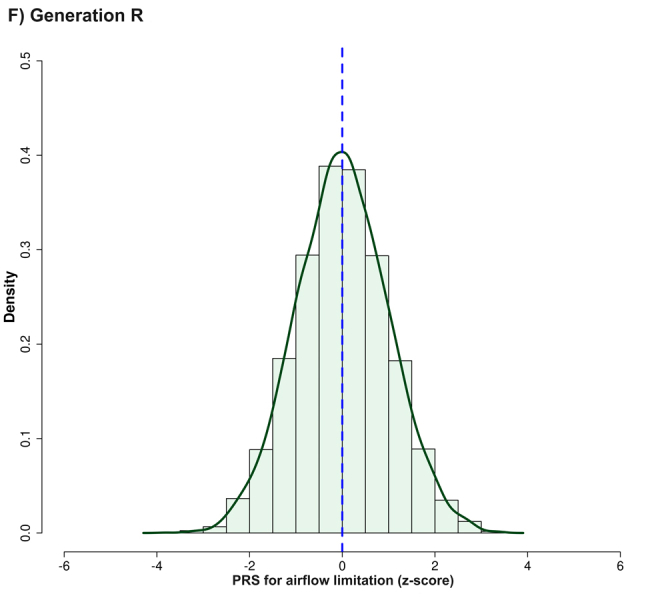


**Supplementary Figure S2G:**
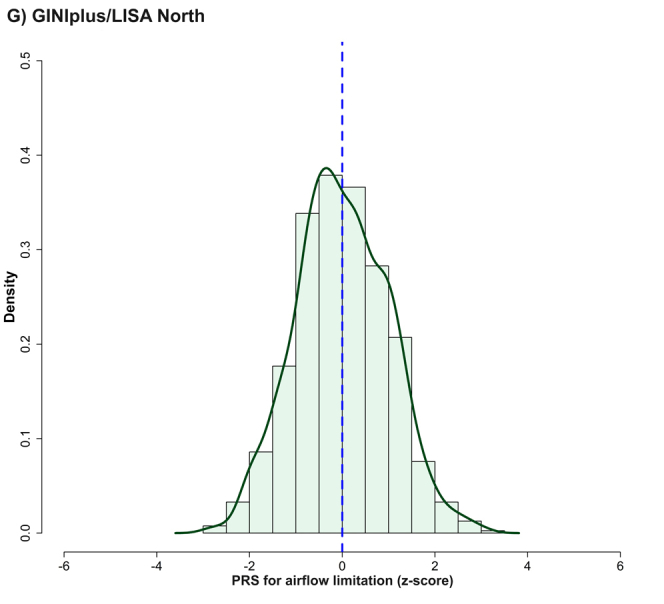


**Supplementary Figure S2H:**
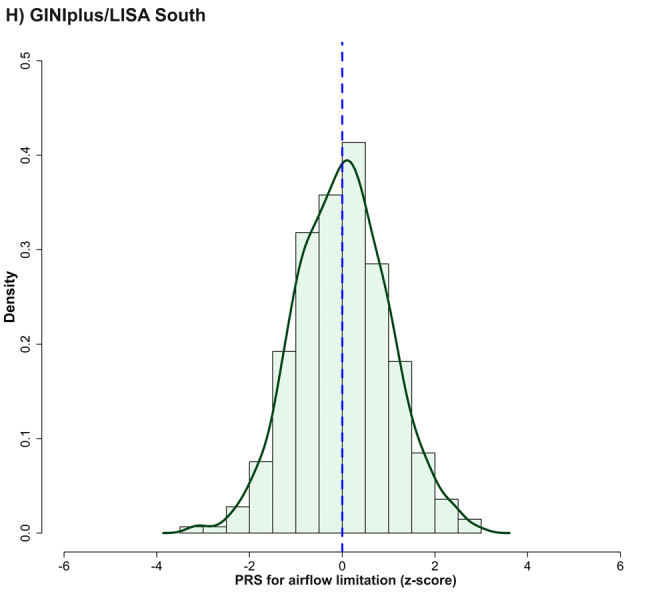


**Supplementary Figure S2I:**
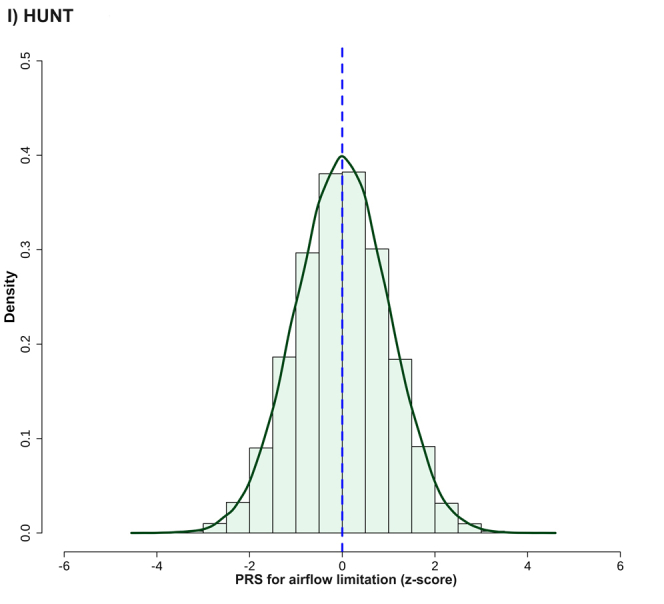


**Supplementary Figure S2J:**
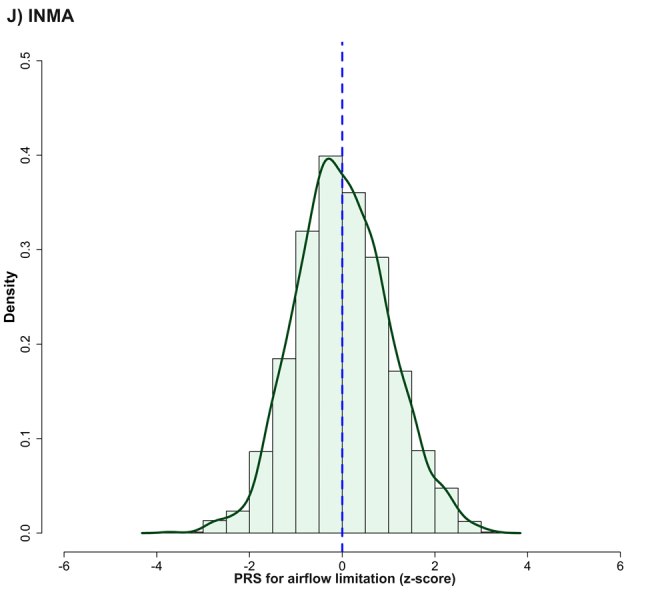


**Supplementary Figure S2K:**
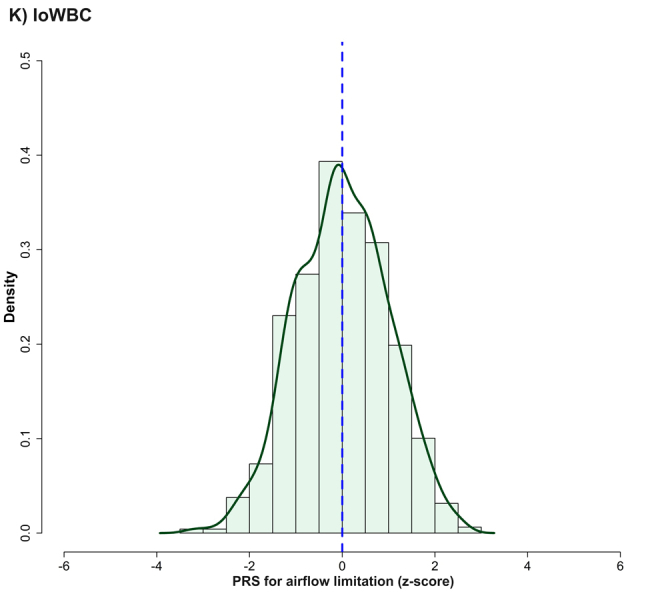


**Supplementary Figure S2L:**
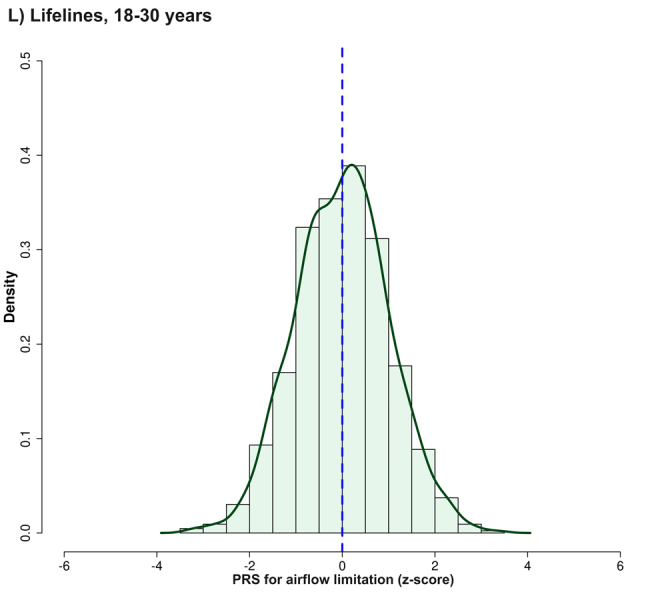


**Supplementary Figure S2L:**
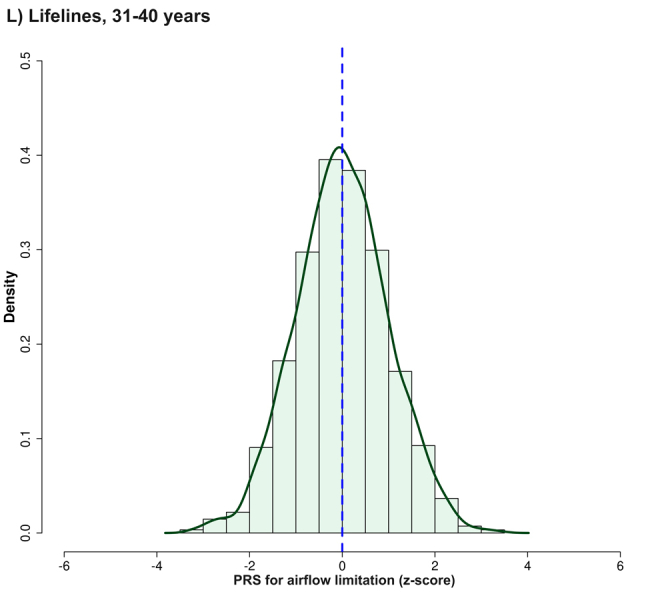


**Supplementary Figure S2M:**
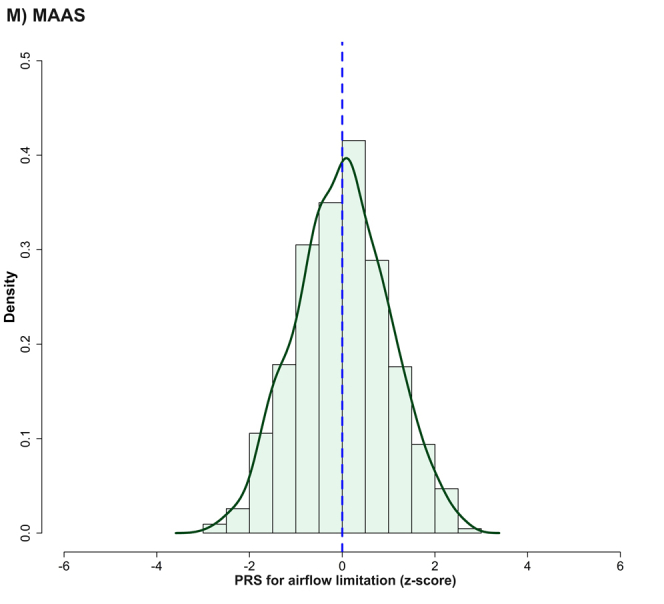


**Supplementary Figure S2N:**
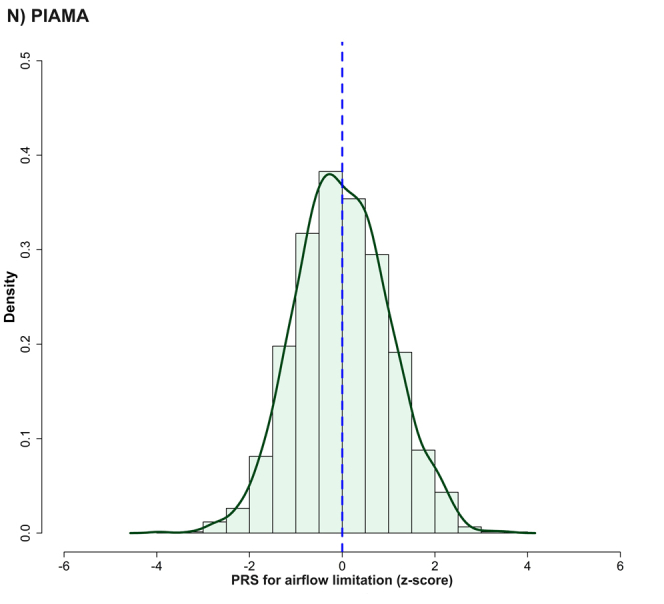


**Supplementary Figure S2O:**
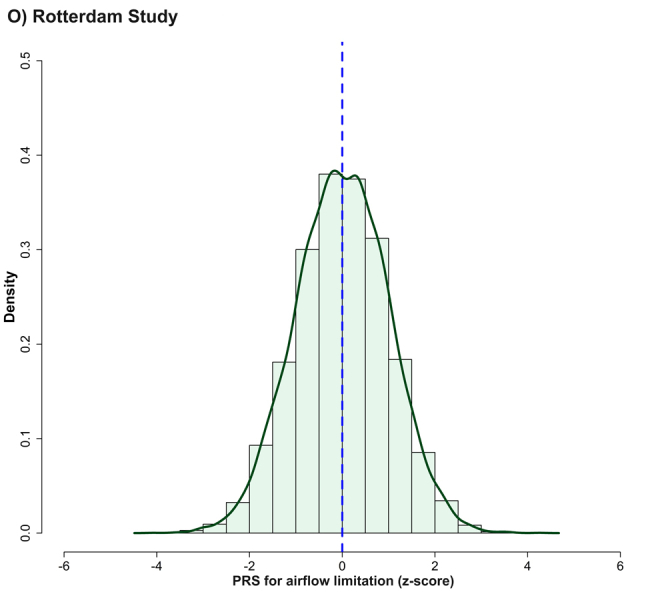


**Supplementary Figure S2P:**
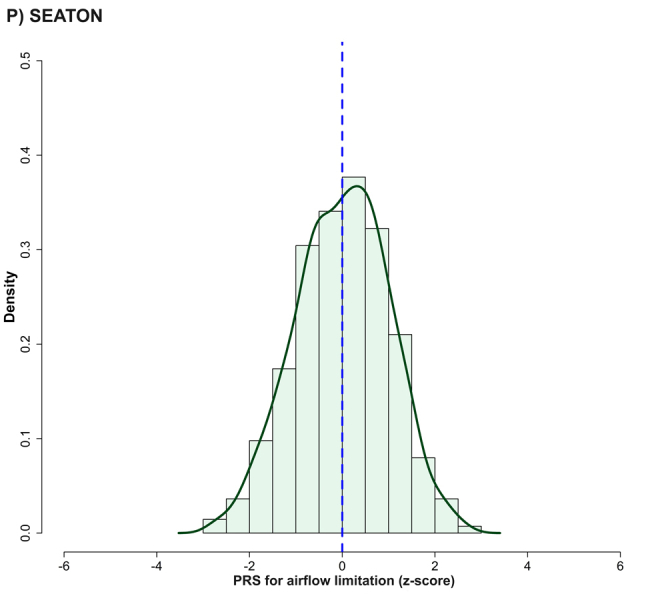


**Supplementary Figure S4A:**
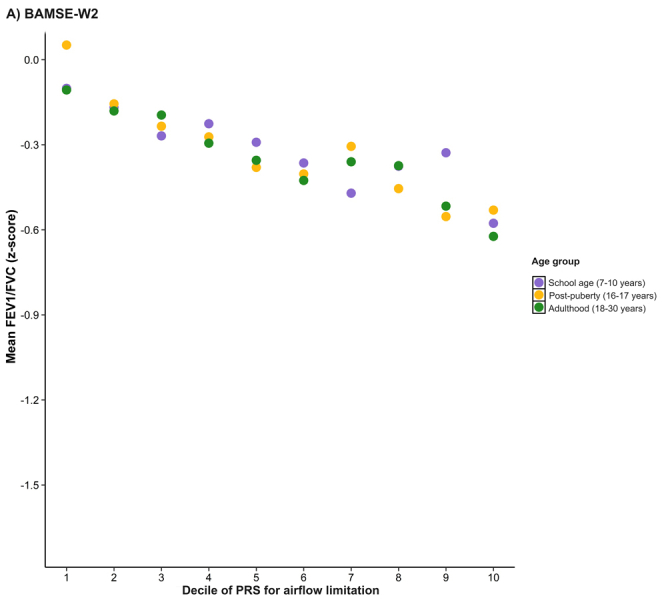


**Supplementary Figure S4B:**
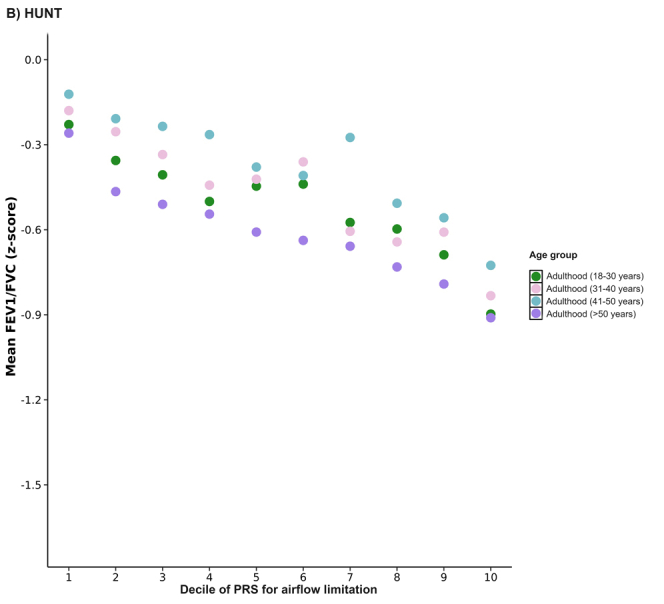


**Supplementary Figure S5A:**
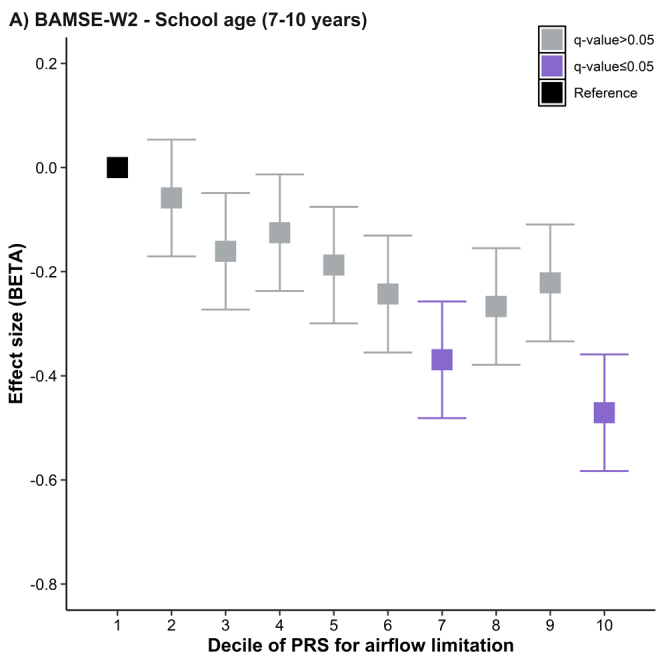


**Supplementary Figure S5B:**
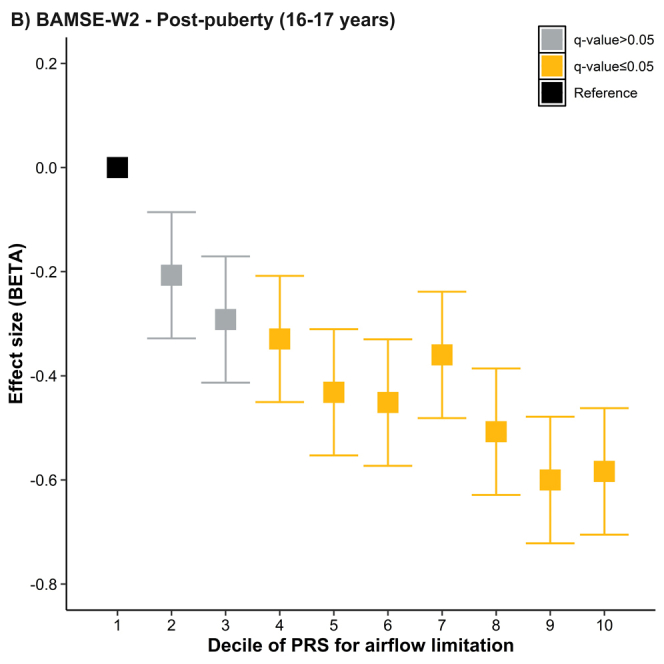


**Supplementary Figure S5C:**
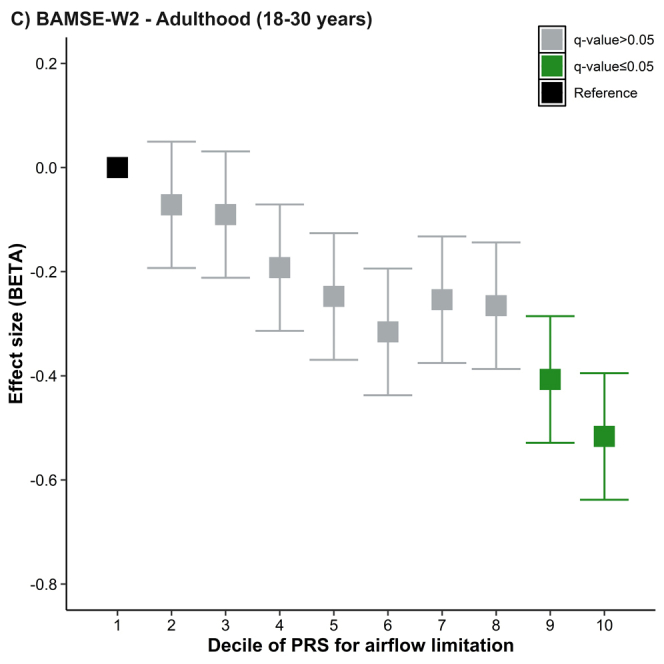


**Supplementary Figure S5D:**
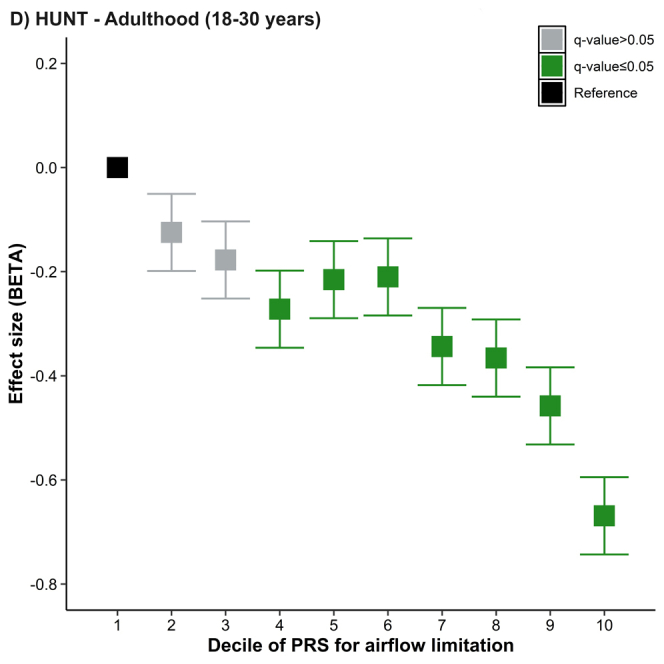


**Supplementary Figure S5E:**
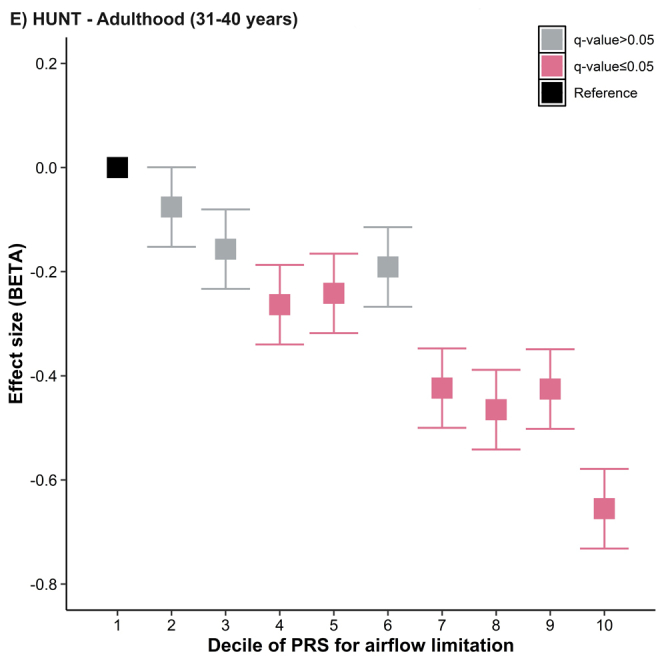


**Supplementary Figure S5F:**
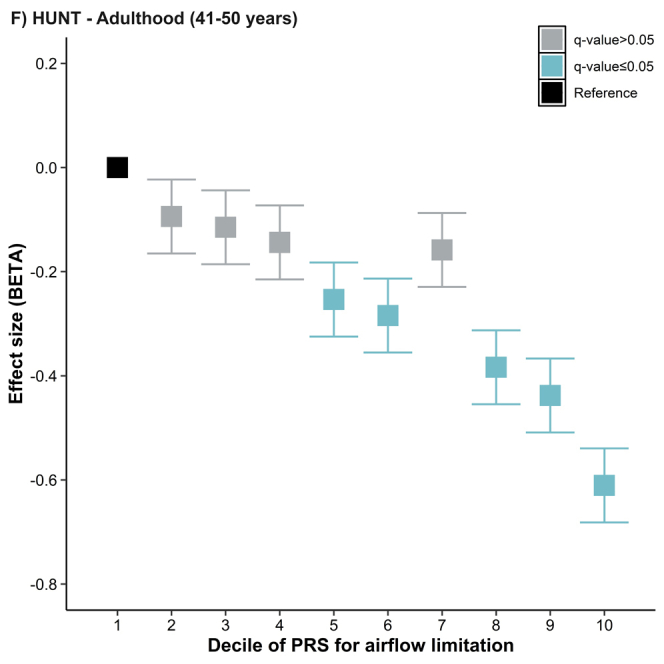


**Supplementary Figure S5G:**
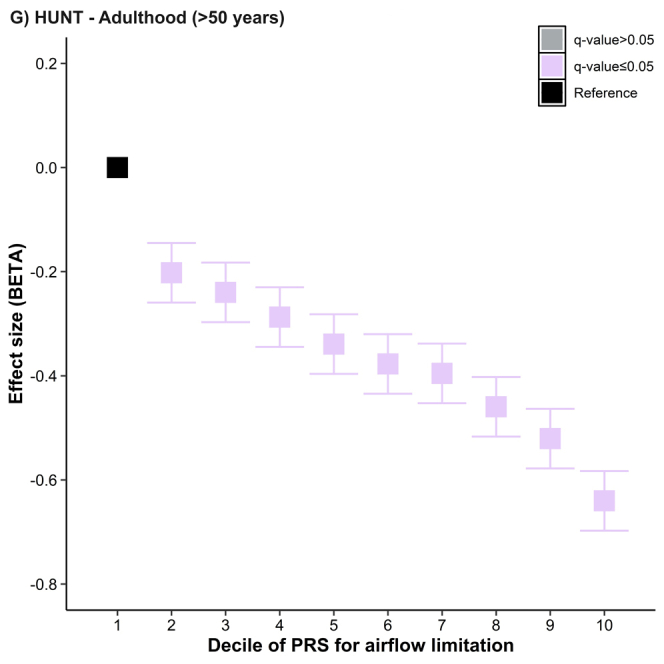


**Supplementary Figure S8:**
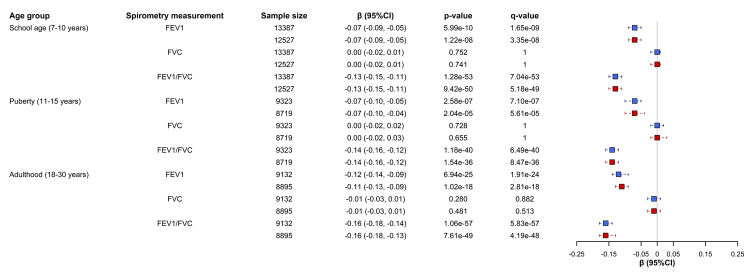


**Supplementary Figure S11A:**
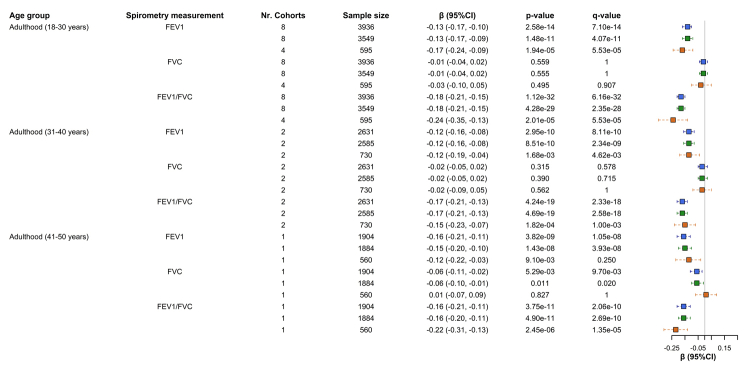


**Supplementary Figure S11B:**
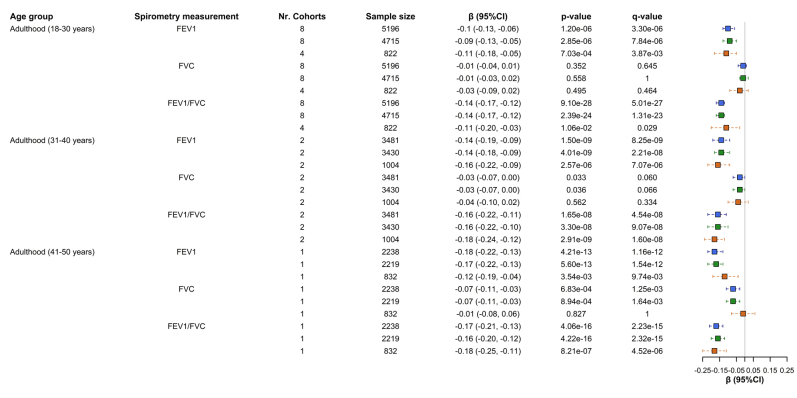


**Supplementary Figure S12A:**
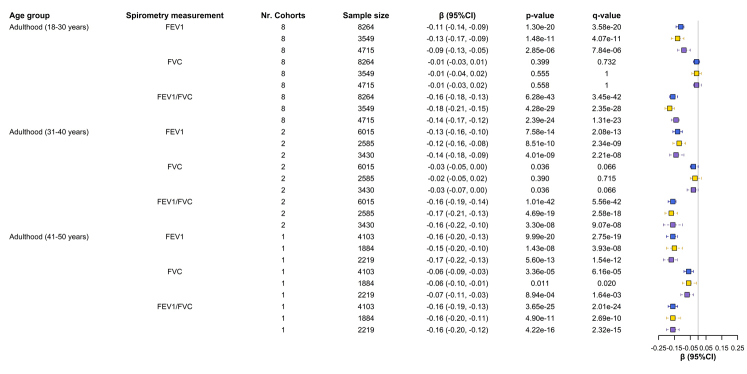


**Supplementary Figure S12B:**