# Supplementary material for: Exploring the genetics of airflow limitation in lung function across the lifespan – a polygenic risk score study
Source: eClinicalMedicine. 2024 Aug 12;75:102731. doi: 10.1016/j.eclinm.2024.102731 (PMC11577569; doi:10.1016/j.eclinm.2024.102731)
Supplement: Supplementary Tables and Figures [file mmc1.pdf]

SUPPLEMENTARY MATERIAL

Exploring the genetics of airflow limitation in lung function across the lifespan – a polygenic risk score study

Natalia Hernandez-Pacheco, PhD[1*], Anna Kilanowski, PhD[2,3*], Ashish Kumar, MSc[1*], John A. Curtin, PhD[4*], Núria Olvera, MSc[5,6*], Sara Kress, PhD[7*], Xander Bertels, MSc[8,9*], Lies Lahousse, PhD[8,9*], Laxmi Bhatta, PhD[10,11,12*], Raquel Granell, PhD[13*], Sergi Marí, MSc[14*], Jose Ramon Bilbao, PhD[14,15*], Yidan Sun, MSc[16*], Casper-Emil Tingskov Pedersen, PhD[17*], Tarik Karramass, MD[18,19*], Elisabeth Thiering, PhD[2,3], Christina Dardani, PhD[13], Simon Kebede Merid, PhD[1], Gang Wang, PhD[1,20], Jenny Hallberg, PhD[1,21], Sarah Koch, PhD[22,23,24], Judith Garcia-Aymerich, PhD[22,23,24], Ana Esplugues, PhD[24,25,26], Maties Torrent, PhD[27], Jesus Ibarluzea, PhD[15,28,29,30], Lesley Lowe, PhD[4], Angela Simpson, PhD[4], Ulrike Gehring, PhD[31], Roel C. H. Vermeulen, PhD[31], Graham Roberts, PhD[32,33,34], Anna Bergström, PhD[35,36], Judith M. Vonk, PhD[37,38], Janine F. Felix, PhD[18,39], Liesbeth Duijts, PhD[18,19], Klaus Bønnelykke, PhD[17], Nic Timpson, PhD[13], Guy Brusselle, PhD[40,41], Ben M. Brumpton, PhD[10,11], Arnulf Langhammer, PhD[42], Stephen Turner, PhD[43], John W. Holloway, PhD[33,34], Syed Hasan Arshad, PhD[32,33,44], Anhar Ullah, MSc[45], Adnan Custovic, PhD[45], Paul Cullinan, PhD[45], Clare S. Murray, PhD[4], Maarten van den Berge, PhD[16,38], Inger Kull, PhD[1], Tamara Schikowski, PhD[7], Jadwiga A. Wedzicha, PhD[45], Gerard Koppelman, PhD[16,38], Rosa Faner, PhD[5,6*], Àlvar Agustí, PhD[5,6,46,47*], Marie Standl, PhD[2,48*], Erik Melén, PhD[1,21*], on behalf of the CADSET Clinical Research Collaboration of the European Respiratory Society

[1]Department of Clinical Science and Education, Södersjukhuset, Karolinska Institutet, Sjukhusbacken 10, 118 83 Stockholm, Sweden.

[2]Institute of Epidemiology, Helmholtz Zentrum München – German Research Center for Environmental Health, Campus Neuherberg, Ingolstädter Landstraße 1, 85764 Neuherberg, Germany.

[3]Division of Metabolic and Nutritional Medicine, Dr. von Hauner Children's Hospital, University of Munich Medical Center, Lindwurmstraße 4, 80337 Munich, Germany.

[4]Division of Infection, Immunity and Respiratory Medicine, School of Biological Sciences, The University of Manchester, Manchester Academic Health Science Centre, and Manchester University NHS Foundation Trust, Cobbett House Manchester Royal Infirmary, Oxford Rd, Manchester M13 9WL, United Kingdom.

[5]CIBER de Enfermedades Respiratorias (CIBERES), Spain.

[6]Universitat de Barcelona, Departament de Biomedicina, Institut d'investigacions Biomediques August Pi I Sunyer (IDIBAPS), Calle Rosselló 149, 08036 Barcelona, Spain.

[7]IUF – Leibniz Research Institute for Environmental Medicine, Auf'm Hennekamp 50, 40225 Düsseldorf, Germany.

[8]Department of Bioanalysis, Faculty of Pharmaceutical Sciences, Ghent University, Ottergemsesteenweg 460, 9000 Ghent, Belgium.

[9]Department of Epidemiology, Erasmus MC, University Medical Center Rotterdam, PO Box 2040, Rotterdam 3000, CA, The Netherlands.

[10]K.G. Jebsen Center for Genetic Epidemiology, Department of Public Health and Nursing, NTNU, Norwegian University of Science and Technology, Håkon Jarls gt.11, 7491 Trondheim, Norway.

[11]HUNT Research Centre, Department of Public Health and Nursing, Faculty of Medicine and Health Sciences, NTNU, S.P. Andersens veg 11, 7031 Trondheim, Norway.

[12]Division of Mental Health Care, St. Olavs Hospital, Trondheim University Hospital, Olav Kyrres gate 9, 7030 Trondheim, Norway.

[13]Medical Research Council Integrative Epidemiology Unit (MRC-IEU), Population Health Sciences, Bristol Medical School, Faculty of Health Sciences, University of Bristol, 5 Tyndall Ave, Bristol BS8 1UD, United Kingdom.

[14]Biobizkaia Health Research Institute, University of the Basque Country (UPV/EHU), Leioa, 48940 Bizkaia, Spain.

[15]CIBER Diabetes y Enfermedades Metabólicas asociadas (CIBEDEM), Spain.

[16]University of Groningen, University Medical Center Groningen, Beatrix Children's Hospital, Department of Pediatric Pulmonology and Pediatric Allergology, Hanzeplein 1, 9713 GZ Groningen, The Netherlands.

[17]COPSAC, Copenhagen Prospective Studies on Asthma in Childhood, Herlev and Gentofte Hospital, Ledreborg alle 34, 2820, Gentofte, Denmark.
[18]The Generation R Study Group, Erasmus MC, University Medical Center, Dr. Molewaterplein 40, 3015 GD Rotterdam, The Netherlands.
[19]Department of Pediatrics, Division of Respiratory Medicine and Allergology, Erasmus MC, University Medical Center, Dr. Molewaterplein 40, 3015 GD Rotterdam, The Netherlands.
[20]Institute of Integrated Traditional Chinese and Western Medicine, West China Hospital, Sichuan University, 17 Renmin South Rd Section 3, 小天竺 Wuhou District, Chengdu, Sichuan, China, 610041.
[21]Sachs' Children and Youth Hospital, Södersjukhuset, Hjalmar Cederströms gata 14, 118 61 Stockholm, Sweden.
[22]ISGlobal, Barcelona, Spain.
[23]Universitat Pompeu Fabra, Barcelona, Spain.
[24]CIBER Epidemiología y Salud Pública (CIBERESP), Spain.
[25]Department of Nursing, University of Valencia, Avenida de Menéndez y Pelayo, 19, 46010 Valencia, Spain.
[26]FISABIO-Universitat Jaume I-Universitat de València Joint Research Unit of Epidemiology and Environmental Health, Av. de Catalunya, 21, 46020 Valencia, Spain.
[27]Ib-Salut, Area de Salut de Menorca, Palma, Spain.
[28]Biodonostia Health Research Institute, Group of Environmental Epidemiology and Child Development, Paseo Doctor Begiristain S/n, 20014, San Sebastian, Spain.
[29]Department of Health of the Basque Government, Subdirectorate of Public Health of Gipuzkoa, Avenida Navarra 4, 20013, San Sebastian, Spain.
[30]Faculty of Psychology, University of the Basque Country (UPV/EHU), 20008, San Sebastian, Spain.
[31]Institute for Risk Assessment Sciences, Utrecht University, Institute for Risk Assessment Sciences, Utrecht University, Utrecht, The Netherlands.
[32]David Hide Asthma and Allergy Research Centre, St Marys Hospital Nhs Trust, Newport PO30 5TG, United Kingdom.
[33]NIHR Southampton Biomedical Research Centre, University Hospitals Southampton NHS Foundation Trust, Tremona Road, Southampton, SO16 6YD, United Kingdom.
[34]Human Development and Health, Faculty of Medicine, University of Southampton, University of Southampton, 12 University Rd, Southampton SO17 1BJ, United Kingdom.
[35]Institute of Environmental Medicine, Karolinska Institutet, Nobels väg 13, 171 65 Solna, Stockholm, Sweden.
[36]Centre for Occupational and Environmental Medicine, Region Stockholm, Torsplan, Solnavägen 4, 113 65 Stockholm, Sweden.
[37]Department of Epidemiology, University Medical Center Groningen, University of Groningen, Hanzeplein 1, 9713 GZ Groningen, The Netherlands.
[38]Groningen Research Institute for Asthma and COPD (GRIAC), University of Groningne, University Medical Center Groningen, Hanzeplein 1, 9713 GZ Groningen, The Netherlands.
[39]Department of Pediatrics, Erasmus MC, University Medical Center, Dr. Molewaterplein 40, 3015 GD Rotterdam, The Netherlands.
[40]Department of Respiratory Medicine, Ghent University Hospital, Corneel Heymanslaan 10, 9000 Ghent, Belgium.
[41]Departments of Epidemiology and Respiratory Medicine, Erasmus MC, University Medical Center Rotterdam, PO Box 2040, Rotterdam 3000, CA, The Netherlands.
[42]Department of Levanger Hospital, Nord-Trøndelag Hospital Trust, Helse Nord-Trøndelag, 7601 Levanger, Norway.
[43]Royal Aberdeen Children's Hospital NHS Grampian, Westburn Rd, Aberdeen AB25 2ZG, United Kingdom.
[44]Clinical and Experimental Sciences, Faculty of Medicine, University of Southampton, 12 University Rd, Southampton SO17 1BJ, United Kingdom.
[45]National Heart and Lung Institute, Imperial College London, St Mary's Campus Medical School, Norfolk Place, London W2 1PG, United Kingdom.
[46]Cátedra de Salud Respiratoria, University of Barcelona, Calle Casanovas, 143, 08036 Barcelona, Spain.
[47]Pulmonary Service, Respiratory Institute, Hospital Clinic, Calle Villarroel, 170, 08036 Barcelona, Spain.
[48]German Center for Lung Research (DZL), Aulweg 130, 35392 Gießen, Munich, Germany.

** These authors contributed equally to this work.*

TABLE OF CONTENTS

1 **SUPPLEMENTARY METHODS ... 4**
2 Characteristics of each participating CADSET cohort ... 4
3 Calculation of a polygenic risk score for airflow limitation in different populations ... 13
4 Association testing of the PRS for airflow limitation with spirometry measurements in each study ... 14
5 Estimation of the variance in lung function explained by the PRS for airflow limitation ... 16
6 Sensitivity analyses ... 16
7 **SUPPLEMENTARY RESULTS ... 16**
8 Characteristics of the participating CADSET cohorts ... 16
9 Calculation of a polygenic risk score for airflow limitation in different cohorts ... 17
10 Association testing with spirometry measurements ... 18
11 Estimation of the variance in lung function explained by the PRS for airflow limitation across the lifespan 19
12 Sensitivity analyses ... 19
13 **SUPPLEMENTARY REFERENCES ... 21**
14 **SUPPLEMENTARY FIGURE LEGENDS ... 23**
15 **SUPPLEMENTARY TABLES ... 27**

SUPPLEMENTARY METHODS

Characteristics of each participating CADSET cohort

ALSPAC

Recruitment and study design

Pregnant women residents in Avon (United Kingdom) with expected dates of delivery from 1st April 1991 to 31st December 1992 were invited to take part in the Avon Longitudinal Study of Parents and Children (ALSPAC). The initial number of pregnancies enrolled is 14,541 (for these at least one questionnaire has been returned or a "Children in Focus" clinic had been attended by 19/07/99). Of these initial pregnancies, there was a total of 14,676 fetuses, resulting in 14,062 live births and 13,988 children who were alive at 1 year of age.

When the oldest children were approximately 7 years of age, an attempt was made to bolster the initial sample with eligible cases who had failed to join the study originally. As a result, when considering variables collected from the age of seven onwards (and potentially abstracted from obstetric notes) there are data available for more than the 14,541 pregnancies mentioned above. The number of new pregnancies not in the initial sample (known as Phase I enrolment) that are currently represented on the built files and reflecting enrolment status at the age of 24 is 913 (456, 262, and 195 recruited during Phases II, III and IV respectively), resulting in an additional 913 children being enrolled. The phases of enrolment are described in more detail in the cohort profile paper and its update. The total sample size for analyses using any data collected after the age of seven is therefore 15,454 pregnancies, resulting in 15,589 fetuses. Of these 14,901 were alive at 1 year of age.

A 10% sample of the ALSPAC cohort, known as the Children in Focus (CiF) group, attended clinics at the University of Bristol at various time intervals between 4 to 61 months of age. The CiF group was chosen at random from the last 6 months of ALSPAC births (1432 families attended at least one clinic). Excluded were those mothers who had moved out of the area or were lost to follow-up, and those partaking in another study of infant development in Avon. Lung function information collected at the age of 8, 15, and 24 years was used for the current study. Study data were collected and managed using REDCap electronic data capture tools hosted at the University of Bristol. REDCap (Research Electronic Data Capture) is a secure, web-based software platform designed to support data capture for research studies.[1]

Ethical approval for the study was obtained from the ALSPAC Ethics and Law Committee and the Local Research Ethics Committees. Further details are available in the cohort profile article[2–5] and the study website contains details of all the data that is available through a fully searchable data dictionary and variable search tool: http://www.bristol.ac.uk/alspac/researchers/our-data/. Informed consent for the use of data collected via questionnaires and clinics was obtained from participants following the recommendations of the ALSPAC Ethics and Law Committee at the time.

We are extremely grateful to all the families who took part in this study, the midwives for their help in recruiting them, and the whole ALSPAC team, which includes interviewers, computer and laboratory technicians, clerical workers, research scientists, volunteers, managers, receptionists, and nurses.

Genotyping and quality control analyses

Samples from ALSPAC children were genotyped using the Illumina HumanHap550 BeadChip (Illumina) by the Wellcome Trust Sanger Institute, Cambridge (United Kingdom) and the Laboratory Corporation of America, Burlington, NC, using support from 23andMe. The resulting genotype data were subjected to standard quality control (QC) procedures, excluding samples based on sex mismatches, minimal or excessive heterozygosity, disproportionate levels of individual missingness (>3%), and insufficient sample replication. Population stratification was assessed by multidimensional scaling analysis and compared with Hapmap II (release 22) European descent (CEU), Han Chinese, Japanese, and Yoruba reference populations, whereas individuals with non-European ancestry were removed. On the other hand, single nucleotide polymorphisms (SNPs) with a minor allele frequency (MAF) <1%, a call rate (CR) <95%, or evidence for violations of the Hardy-Weinberg equilibrium (HWE) ($p<5x10^{-7}$) were removed. Cryptic relatedness was measured as the proportion of identity by descent (IBD >0·1). Related subjects that passed all other quality control thresholds were retained during subsequent phasing and imputation. As a result, a total of 9,115 subjects and 500,527 SNPs passed these QC filters.[6]

Spirometry

Spirometry tests were conducted at $8^{1/2}$, 15, and 24 years according to American Thoracic Society (ATS)/European Respiratory Society (ERS) guidelines[7] using a Vitalograph pneumotachograph system with

animated incentive software (Spirotrac, Vitaograph, United Kingdom) in a dedicated research clinic by trained technicians. Calibration checks were performed with a standard 3L calibration syringe according to the manufacturer's instructions at the start of each half-day clinic session. Subjects were seated with a nose clip in place and were asked to inhale to total lung capacity (TLC), then instructed to perform a forced expiration, through a mouthpiece, to residual volume (RV). The test was repeated at intervals of 30 seconds until 3 technically acceptable traces were obtained from a maximum of eight attempts. Forced expiratory volume in one second (FEV_1) and forced vital capacity (FVC) were recorded and the data expressed as FEV_1 % predicted and FEV_1/FVC ratio.

Ashford

Recruitment and study design

The Ashford study is a birth cohort that included 658 pregnant women who visited three general practice centers in Ashford, Kent (United Kingdom) for antenatal care over 18 months in 1993. Among these, 625 women gave birth, and a total of 642 newborns were recruited. A skin prick test (SPT) for three common aeroallergens was conducted on both parents at the time of recruitment, and reports of a personal history of asthma, hayfever, or childhood eczema were requested. Children's mothers were annually interviewed for the following 6 years using a standardized questionnaire to obtain reports of their child's wheezing symptoms in the last year. Information related to environmental exposure at home and socioeconomic status was also collected. Children have been followed prospectively and attended review clinics at ages 1, 2, 4, 5, and 14 years when a detailed questionnaire was administered by a specialized interviewer.

Genotyping and quality control analyses

Genotyping was performed using the Illumina Human610-Quad BeadChip (Illumina) platform. Subjects with sex mismatches, minimal or excessive heterozygosity, and genotyping CR <97% were discarded. Exclusion criteria for genetic variants consisted of CR <95%, MAF <0·5%, and HWE $p<3x10^{-8}$.[6]

Spirometry

Spirometry was assessed according to the ATS/ERS recommendations at the age of 10 and 15 years.[7]

BAMSE

Recruitment and study design

The Children Allergy Milieu Stockholm Epidemiology (BAMSE) study is a prospective ongoing population-based cohort that recruited a total of 4,089 infants born between 1994 and 1996 in Stockholm (Sweden). A questionnaire about environmental exposures and allergy-related symptoms was obtained from parents when the children were two months old and followed up during childhood and adolescence (1, 2, 4, 8, 12, and 16 years of age). Information about environmental exposures, lifestyle, socioeconomic status, allergy-related or respiratory symptoms, and medication use has also been collected. Subjects have been invited at several time points for extensive clinical examination, including lung function assessment, and collection of different biological samples. At the 24-year-old follow-up visit, 3,064 participants filled out questionnaires themselves, and complete clinical data, blood, nasal, and urine samples were collected from 2,617 individuals. This study was restricted to individuals included in the follow-up visits at the age of 8, 16, or 24 years old when lung function was evaluated through spirometry. Further details have been described elsewhere.[8–13]

Genotyping and quality control analyses

DNA was extracted from blood collected at the age of 8 and 16 years and genotyping was conducted in two subsets of participants, hereon referred to as "Waves" with the Illumina Human 610-quad array (Illumina, Inc.) (Wave 1, n=505) and the Illumina Infinium Global Screening Array-24 v1.0 BeadChip (Illumina, Inc.) (Wave 2, n=2,387). Given the differences in the genotyping platform used, data from each Wave are usually separately analyzed. QC analyses were performed at the individual and genetic markers levels in each Wave using PLINK 2.0.[14,15] Subjects with CR <98% and heterozygosity rates higher or lower than 4 standard deviations of the population mean were discarded. Moreover, individuals with discordances between the reported and genetic sex based on the genotype data from the X chromosome were discarded, as well as subjects with evidence of relatedness for at least second-degree relatives (PIHAT ≥0·2). Duplicated samples included in both genotyped Waves were excluded, retaining it only in the subset with a higher CR. A Principal Component (PC) analysis was carried out using PLINK 2.0[14,15] to inspect the existence of individuals with large differences in ancestry. After representing the ten first PCs of the study participants projected on the samples from different populations included in the 1,000 Genome Project reference panel,[16] those subjects identified as outliers of the distribution of European individuals were excluded, resulting in 2,636 samples that passed the QC procedures (Wave 1,

n=463; Wave 2, n=2,173). On the other hand, genetic variants with CR <95%, deviations from the HWE (p<1x10^{-6}), and MAF <0·01 were discarded from further analyses.

Spirometry

Lung function was assessed by spirometry at 8 years of age using the 2200 Pulmonary Function Laboratory (Sensormedics, Anaheim, California, United States), at age 16 years using the Jaeger Masterscreen-IOS system (Care Fusion, San Diego, California, United States), and at 24 years of age using the Jaeger MasterScreen-IOS system (Carefusion Technologies, San Diego, California, United States). Each subject was instructed to perform at least three MEFV recordings in the sitting position and wearing a nose clip. The highest values of FVC and FEV_1 were extracted, provided that the subject's effort was coded as being maximal by the test leader, the MEFV curve passed visual quality inspection, and the two highest FEV_1 and FVC readings were reproducible according to the ATS/ERS guidelines.[7,17]

$COPSAC_{2000}$

Recruitment and study design

The Copenhagen Prospective Studies on Asthma in Childhood (COPSAC) 2000 is a single-center prospective clinical birth cohort that includes 411 children of mothers with asthma attending prenatal clinics from greater Copenhagen, Denmark. This study was designed to investigate gene-environment interactions in infants and children with high risk for asthma, atopic dermatitis, and allergic rhinitis in order to identify exposures in early life that could be modified to prevent the development of these diseases. The inclusion criteria included being pregnant, a physician's diagnosis, and a history of daily asthma treatment use for mothers. Infants were enrolled in the cohort one month after birth between 1998 and 2001. A severe congenital anomaly, a gestational age younger than 36 weeks, a need for mechanical ventilation, or a lower respiratory tract infection were considered reasons for exclusion. Children were monitored from birth until 7 years of age every 6 months and at the age of 13 and 18 years. Information about socioeconomic status, mother's pregnancy, pre-natal exposures, any diseases, and medication use were collected from mothers. Participants were invited to clinical examination visits when extensive physical tests were undertaken at each follow-up, as well as spirometry measurement from 5 years of age and collection of detailed information about the history and diagnosis of atopic diseases, growth, mental health, and environmental exposures. A wide range of biological samples was also collected at several time points.[18]

Genotyping and quality control analyses

DNA was extracted from blood samples and genotyping was carried out using the Illumina HumanOmniExpressExome 8 v1-2 BeadChip at the AROS Applied Biotechnology AS Center, Aarhus, Denmark. Several QC procedures on genotype data were performed using PLINK 1.9.[15] Genotype calling was conducted using the Illumina GenCall Data Analysis tool. SNPs with MAF >0·01, CR >99%, cluster separation score ≥0·3, and no evidence of deviations from the Hardy-Weinberg equilibrium (p>1x10^{-6}) were selected. Individuals with CR ≤99%, sex discordances, non-European ancestry, genetic duplicates, or monozygotic twins were excluded.[19]

Spirometry

Lung function was assessed at 7, 12, and 18 years of age by spirometry using a pneumotachograph Masterscope Pneumoscreen, system 754,916 spirometer (Erich Jaeger, Wurtzburg, Germany), measuring FEV_1 and $FEV_{0.5}$. Airway reversibility was determined as the change in FEV_1 after inhalation of 2 doses of 0.25 mg of short-acting β2-agonist pressurized metered-dose inhaler (Terbutaline; AstraZeneca, Lund, Sweden) administered through a nonelectrostatic spacer with a facemask.[20]

$COPSAC_{2010}$

Recruitment and study design

$COPSAC_{2010}$ is a Danish population-based clinical cohort of 736 pregnant women and their 700 children built on $COPSAC_{2000}$. It was designed to reduce the burden of asthma and other wheezing-related disorders, eczema, and allergy. Women were enrolled in the 22nd-26th gestational week between 2008 and 2010. Participating children were followed up at the age of 1 week, 1, 3, 6, 12, 18, 24, 30, and 36 months, 4, 5, 6, 8, and 10 years. Complete information about the history of atopic diseases, lung function, growth pattern, and neurological conditions have been collected, as well as a wide range of environmental exposures and biological samples.

Genotyping and quality control analyses

Samples were genotyped and subsequent QC procedures were conducted following the same approach described for $COPSAC_{2000}$.

Spirometry

Lung function was assessed at 10 years of age using the same approach as described for $COPSAC_{2000}$.

Generation R

Recruitment and study design

The Generation R Study is a multi-ethnic multidisciplinary population-based prospective cohort study from early fetal life onwards in Rotterdam, the Netherlands.[21] The study has been approved by the Medical Ethical Committee of the Erasmus MC, University Medical Center in Rotterdam.[22] Written informed consent was obtained from all participants. Eligible mothers were those who were residents in the study area at their delivery date and had an expected delivery date from April 2002 until January 2006. Mothers were mainly enrolled in early pregnancy, but enrolment was possible until the birth of their child. In total, 9,778 mothers were enrolled in the study. Of these mothers, 91% (n = 8,879) were enrolled during pregnancy. Partners from mothers enrolled in pregnancy were invited to participate. In total, 71% (n = 6,347) of all fathers were included. The 9,778 mothers enrolled in the study gave birth to 9,749 live-born children. During the preschool period (0–4 years), the logistics of the postnatal follow-up studies were embedded in the municipal routine childcare system and restricted to only part of the study area. In total, 1,166 children lived outside this defined study area at birth and were therefore not approached for postnatal follow-up studies during the preschool period. Of the remaining 8,583 children, 690 (8%) parents did not give consent, or their children died or were lost to follow-up, leaving 7,893 children for the preschool studies. At the age of 6 years (early school age), we invited all 9,278 children from the original cohort of 9,749 children to participate in follow-up studies. This invitation was independent of their home address and participation in the preschool period. In total, 8,305 children (90% of those who were invited (n = 9,278) and 85% of the original cohort (n = 9,749)) still participated in the study at this age, of whom 6,690 visited the research center at a median age of 6.0 years. For the follow-up phase at the age of 10 years (mid-childhood period), 730 children of the 9,278 could not be invited. In total, 7,393 children (86% of those who were invited (n = 8548) and 76% of the original cohort (n = 9,749)) participated in the study in mid-childhood, of whom 5,862 visited the research center at a median age of 9.7 years. For the follow-up phase at the age of 13 years (adolescent period), 603 children of the 9,278 could not be invited. In total, 6,842 children (86% of those who were invited (n = 7,949) and 70% of the original cohort (n = 9,749)) participated in the study during adolescence, of whom 4,973 visited the research center at a median age of 13.5 years. Information on a variety of information such as medical and family history, lifestyle, diet, and socioeconomic status, among others, was collected during each time point by questionnaires and measurements at the research center. Blood and urine samples were collected from mothers and children during each visit. Further details have been described elsewhere.[22]

Genotyping and quality control analyses

DNA from parents and children has been extracted and used for genotyping using TaqMan analyses for individual genetic variants and a genome-wide association scan (GWAS) using the Illumina 670 K platform in the children. For genotyping, we used the infrastructure of the Human Genomics Facility (HuGe-F) of the Genetic Laboratory of the Department of Internal Medicine (www.glimdna.org). The GWAS dataset underwent a stringent QC process, which has been described elsewhere.[22]

Spirometry

Lung function was assessed by spirometry (MasterScreen-Pneumo, Jaeger Toennies (Viasys) CareFusion Netherlands) in the Generation R research center on two time-points at the median age of 9.74 (quartile range 9.62 – 9.86) and a median age of 13.62 years (quartile range 13.54 – 13.72), according to the ATS/ERS recommendations.[23] Lung function parameters included FVC, FEV_1, the Tiffeneau-index (FEV_1/FVC), mid-expiratory flow (FEF_{25-75}), and forced expiratory flow after exhaling 75% of FVC (FEF_{75}). Values were converted into sex-, age-, height- and ethnicity-adjusted z-scores based on the Global Lung Function Initiative (GLI) reference values.[24]

GINIplus/LISA North

Recruitment and study design

The German Infant Study on the Influence of Nutrition Intervention plus Air pollution and Genetics on Allergy Development (GINIplus) and the Influence of Life-style factors on Development of the Immune System and Allergies in East and West Germany (LISA) studies are prospective population-based birth cohorts initially conceived for the investigation of the development of allergic diseases. In GINIplus, a total of 5,991 full-term and normal-weighted newborns were recruited between 1995 and 1998 in Munich and Wesel (Germany). The LISA study included 3,097 healthy children born between 1997 and 1999 in Munich, Wesel, Bad Honnef, and Leipzig (Germany). Both studies followed up their participants in a harmonized and comparable way at the age of 1, 2, 4, 6, 10, and 15 years in GINIplus and at 0.5, 1, 1.5, 2, 4, 6, 10, and 15 years in LISA. Spirometry measurements were conducted on a subset of participants at ages 6, 10, and 15 years.

Data from GINIplus and LISA have been pooled due to a harmonized study design and follow-up approach, thus these are referred to as GINIplus/LISA. In the current study, subjects recruited in Leipzig and Bad Honnef were discarded since genome-wide data was not available for those study areas. Due to different genotyping platforms, data is analyzed separately for the Wesel (GINIplus/LISA North) and Munich (GINIplus/LISA South) study centers. In particular, GINIplus/LISA North includes participants recruited in a predominantly rural area from Wesel and the adjacent regions of Münster and Düsseldorf. For the current study, valid lung function information from the 10 and 15-year follow-ups was evaluated. Detailed study descriptions have been included in previous publications.[25–28]

Genotyping and quality control analyses

Blood and saliva samples collected at 6 and 10 years of age were genotyped using the Illumina Infinium Global Screening Array-24 v2.0 BeadChip (Illumina). Quality control (QC) procedures included SNP level 1: Exclusion of variants on chromosome 0, insert/deletion variants, MAF (<0.01), and call rates (<0.95); Sample level: Removal of duplicated individuals, individuals with sex-mismatch, call rates (<0.95), heterozygosity (inbreeding coefficient 0.1), highly related individuals (identity-by-descent analysis with ld.tresh=0.2 and kin.tresh=0.1), individuals belonging to a different ancestry group (Tukey's rule based on the 1-10 eigenvectors from Principal Component Analysis), and Hardy-Weinberg ($p<10^{-6}$), SNP level 2: Hardy-Weinberg ($p<10^{-6}$), and strand designation/ strand flips corrections. After QC, 792 individuals and 479,023 variants remained. After genotype imputation, the QC of genetic variants including MAF <0.01, exclusion of multi-allelic variants, and imputation quality ($R^2<0.3$) were conducted. Rs-ids using the Haplotype Reference Consortium (HRC) reference manual were merged using chromosome, position, and alleles. After QC, 792 individuals with 7,658,196 SNPs remained.[29]

Spirometry

Lung function was assessed using a pneumotachograph-type spirometer (EasyOne Worldspirometer, ndd, Zürich, Switzerland) was used to obtain flow-volume curves. During the lung function measurements, the children were seated and wearing nose clips. The participants performed at least three but not more than eight trials per test under the guidance of specifically trained and experienced examiners to obtain optimal-flow volume curves. Lung function indices before and after the administration of salbutamol were obtained following the ATS/ERS recommendations.[7] After the completion of baseline spirometry and examination for contraindications, subjects inhaled two puffs of salbutamol delivered into a spacer (Volumatic) by a metered dose inhaler to induce a bronchodilator response. The subject was asked to take five slow deep breaths and hold their breath for five to ten seconds to allow optimal particle deposition. Spirometry was performed 15 minutes after salbutamol inhalation. Although initially a 400 µg dose of salbutamol was used during the pilot phase, this does was reduced to 200 µg six weeks after the study was initiated as unwanted side effects were frequently observed.[30]

GINIplus/LISA South

Recruitment and study design

The same recruitment and follow-up methodologies described above for GINIplus/LISA North were applied. GINIplus/LISA South predominantly comprises children living in urban areas, including the city of Munich and adjacent regions of Upper Bavaria and Swabia.[25–28] Spirometry measurements from subjects aged 6 and 15 years were included in this study.

Genotyping and quality control analyses

Blood and saliva samples collected at the 6 and 10-year follow-up visits were genotyped using the Affymetrix Genome-Wide Human SNP Array 5.0 and Affymetrix Genome-Wide Human SNP Array 6.0 (Thermo Fisher) platforms. Individuals were excluded based on the following criteria: CR <95%, heterozygosity rate higher or lower than 4 standard deviations of the population mean, any failure of the sex check, or the similarity based on IBS using MDS analyses. Variants with CR <95%, MAF <0·01, or HWE $p<1x10^{-5}$) were excluded, remaining 1,511 samples with available high-quality genome-wide genetic data.[31]

Spirometry

Lung function was assessed through spirometry following the same methodology described for GINIplus/LISA North.

HUNT

Recruitment and study design

The Trøndelag Health Study (HUNT) is an ongoing population-based study that invited all inhabitants aged between 13 and 104 years living in Trøndelag County (Norway). Health-related information has been collected from participants through questionnaires, interviews, and clinical examinations. More than 229,000 subjects older than 20 years of age at recruitment) have participated in the study to date, of whom 95,000 have provided at least one biological sample.[32–35] Participants from the Nord Trøndelag area were recruited at four time points: 1984-1986 (HUNT1), 1995-1997 (HUNT2), 2006-2008 (HUNT3), and 2017-2019 (HUNT4). Basic questionnaires were also collected in HUNT4 from adults living in the Sør-Trøndelag region in the last follow-up (n=105,797). Approximately 19,000 adults participated in all the HUNT data collection points. Additionally, data has been collected from nearly 25,000 adolescents aged 13-19 years through four different Young-HUNT surveys. Adults were invited to field stations located in all municipalities, while the data collection was carried out at schools in Young-HUNT. Lung function was assessed through spirometry in subjects included in Young-HUNT1, Young-HUNT3, and adult HUNT2-4 participants.[32–35] For the present study, subjects from HUNT2 and HUNT3 have been selected and classified into four different groups based on age similarity: 20-30, 31-40, 41-50, and ≥51 years. Thus, there are available lung function measurements collected during HUNT2 and HUNT3 follow-ups for some of the participants selected, whereas others have been recruited at one time point.

Genotyping and quality control analyses

DNA samples were obtained from 88,000 HUNT participants. The genetic research in HUNT was initially focused on exploring the genetic determinants of myocardial infarction (MI). Thus, low-pass whole-genome sequencing was conducted on 2,201 samples from early-onset MI patients, and sex- and age-matched controls included in HUNT2 and HUNT3 (HUNT-WGS) served as a HUNT-specific reference panel. A customized reference panel including HRC and HUNT-WGS was used for imputation. Genome-wide genotyping of HUNT2 and HUNT3 subjects (n=70,517) was carried out using the Illumina Infinium CoreExome-24+ BeadChip (Illumina, Inc.). A strict QC protocol was followed, retaining samples and genetic variants that showed CR ≥99%.[36] A total of 69,717 subjects were included in the PRS calculation in this project after the restriction for participants in HUNT2 and HUNT3 and excluding individuals with missing basic demographic information (age and sex).

Spirometry

Lung function measures were obtained using the heated pneumotachograph (Jaeger MasterScope spirometer version 4.15 and 4.25) in HUNT2-3 and Young-HUNT1-3. The participants were seated and wore a nose clip, and extension or flexion of the neck was avoided. Following the ATS/ERS guidelines, subjects were instructed to perform at least three spirometry maneuvers, and the test was considered satisfactory when the two largest FEV_1 and FVC differed by less than 200 mL, but we did not apply the end of the test criterion with exhalation >6 s. Among adolescents, due to the short expiration time it was difficult to evaluate of end of the test, therefore only smoothly ending flow-volume curves with FEV_1/FVC ≤0.95 were accepted. The flow-volume curve with the highest sum of FEV_1 and FVC was retained.[37,38]

INMA

Recruitment and study design

The INfancia y Medio Ambiente – Environment and Childhood (INMA) study is a Spanish prospective population-based mother-child cohort that recruited pregnant women between 1997 and 1998 in Menorca and between 2003 and 2008 in Gipuzkoa, Sabadell, and Valencia. Being at least 16 years old, having the intention

of delivering in the reference hospital, having a singleton pregnancy, and not having any communication problems were the inclusion criteria. This study aims to investigate the influence of pre and postnatal environmental exposures on growth, development, and health from intrauterine life to adolescence. Children have been followed from birth to puberty (12 years of age) in Gipuzkoa, Sabadell, and Valencia and until 18 years in Menorca. Information related to sociodemographic characteristics, lifestyle factors, and respiratory symptoms was obtained from mothers during pregnancy and from the child at each follow-up visit.[39] More information about the INMA project is available online (http://www.proyectoinma.org/). The study was approved by the ethical committees of the centers involved in the study, and written informed consent was obtained from all the participants.

Genotyping and quality control analyses

DNA was extracted from cord blood, whole blood collected at 4 years of age, or saliva samples using the Chemagen protocol at the Spanish National Genotyping Centre (CEGEN). Children with parents with reported Caucasian ethnicity and birthplace in Spain or any other European country and available information were selected for genome-wide genotyping. This was conducted using the Illumina HumanOmni1-Quad BeadChip and the Illumina Infinium Global Screening Array BeadChip (Illumina) at CEGEN (MEN, SAB, VAL cohorts) and the Human Genotyping Facility (HuGeF) from the Department of Internal Medicine, Erasmus MC, The Netherlands (GIP subcohort), respectively. QC procedures were conducted using PLINK.[14,15] Samples were discarded based on CR <98% and/or LRR SD<0·3, as well as sex check, relatedness, heterozygosity rate, and population stratification standard criteria. Genetic variants with CR ≤95%, MAF ≤1%, and HWE $p<1{\cdot}10x10^{-6}$ were filtered out.

Spirometry

Forced spirometry testing was conducted by trained pulmonary function technicians following the ATS/ERS guidelines.[7] Pre-bronchodilator FVC and FEV_1 were measured, and the FEV_1/FVC ratio was derived.[40] Upon collection, all spirometry maneuvers underwent quality control to ensure acceptability and reproducibility criteria were met. Spirometry maneuvers were considered acceptable if the back extrapolated volume (BEV) was below 0.1L, forced expiratory time (FET) was between 1 and 10 seconds, and the shape of the curve was adequate. Criteria used to evaluate the shape of the flow-volume curves were (i) a sharp rise with no signs of hesitation or cough within the first second; (ii) no cough, inhalation, or early termination during the exhalation phase; and (iii) existence of a volume-time plateau shape. All children included in the analysis had a minimum of one acceptable and reproducible spirometry maneuver at every single assessment timepoint.

IoWBC

Recruitment and study design

The Isle of Wight Birth Cohort (IoWBC) is a population-based study that recruited 1,456 newborns between 1st January 1989 and the 28th February 1990 in the Isle of Wight (United Kingdom).[41] Adoptions, perinatal deaths, and refusal for follow-up were exclusion criteria. Participants were followed up from birth to 26 years of age to prospectively investigate the development of asthma and allergic diseases. Validated questionnaires were filled in by the parents at each follow-up. The SPT was performed on 980, 1036, and 853 participants at 4, 10, and 18 years of age to study allergic reactions to common allergens. The diagnosis of any pulmonary conditions was evaluated through spirometry and methacholine challenge tests at the age of 10, 18, and 26 years.[41–43]

Genotyping and quality control analyses

Genome-wide genotype data were obtained using Illumina Human Infinium Omni2.5-8 v1.3 BeadChip (Illumina). Subjects with sex mismatches, minimal or excessive heterozygosity, and genotyping CR <97% were discarded. Exclusion criteria for genetic variants consisted of CR <95%, MAF <0·5%, and HWE $p<3x10^{-8}$ as previously described.[6]

Spirometry

Pre-bronchodilator lung function tests were conducted at 10, 18, and 26 years of age. FVC and FEV_1 were measured using a Koko Spirometer and software with a portable desktop device (both PDS Instrumentation, Louisville, Kentucky, United States). Spirometry was performed and evaluated according to the ATS criteria. The children or adults, respectively, were required to be free of respiratory infection for 2 weeks and not to be taking any oral steroids and were advised to abstain from any β-agonist medication for 6 h and caffeine intake for at least 4 h before lung function assessment.

Lifelines

Recruitment and study design

The Lifelines Cohort Study is a population-based cross-sectional study that recruited patients invited by their general practitioner in three northern provinces from The Netherlands: Friesland, Groningen, and Drenthe. Subjects with any severe psychiatric or physical illness, limited life expectancy (<5 years), or insufficient knowledge of the Dutch language to complete a questionnaire were excluded. The participants received a baseline questionnaire and an invitation to a comprehensive health assessment at the Lifelines research center. Any interested family members were also invited to participate in the study. Additionally, any other inhabitants of these northern provinces could also register themselves via the Lifelines website. An age of 25 and 50 years and living in any of the provinces mentioned above were considered as inclusion criteria. Children could only participate if one of their parents was a study participant as well.[44] For this study, individuals with available spirometry, genetic, and demographic data were included. Moreover, subjects were classified into two groups of similar age (18-30 and 31-40 years old), and the estimation of the polygenic risk score (PRS) of chronic obstructive pulmonary disease (COPD) and the subsequent evaluation of the association with lung function was separately conducted in each of them. This allowed for maximizing the inclusion of cohort participants and for avoiding sample overlap[45] of older participants (n=10,329) already included in the genome-wide association study (GWAS) of COPD susceptibility[46] used as the base dataset for the PRS calculation.

Genotyping and quality control analyses

Samples from independent participants collected at the second baseline assessment visit were genotyped using the Illumina Infinium HumanCytoSNP-12 v2.0 BeadChip (Illumina). Non-Caucasian participants were excluded based on self-reports, IBS, and population stratification analyses. Moreover, the individuals with the best genotyping quality metrics from first-degree pair family relations were selected. Criteria for SNP selection were: MAF >0·001, HWE $p \geq 1x10^{-4}$, and CR ≥95%.

Spirometry

Pre-bronchodilator spirometry was performed using the Welch Allyn SpiroPerfect device (Version 1.6.0.489, PC-based SpiroPerfect with CardioPerfect Workstation software; Welch Allyn) according to the ATS/ERS criteria.[7] FEV_1, FVC, FEF_{25-75} and FEV_1/FVC were measured.[47]

MAAS

Recruitment and study design

The Manchester Asthma and Allergy Study (MAAS) is an unselected birth cohort established in 1995 in Manchester (United Kingdom). All pregnant women from urban and rural areas within 50 square miles of South Manchester and Cheshire (United Kingdom) located within the maternity catchment area of Wythenshawe and Stepping Hill Hospitals were screened for eligibility at antenatal visits (8th-10th week of pregnancy). Among the selected couples, only those that satisfied the inclusion criteria (≤10 weeks of pregnancy, maternal age ≥18 years, and questionnaire and SPT data available for both parents) were recruited. A total of 1,184 children born between February 1996 and April 1998 were included in the study. They were followed prospectively for 19 years to date and attended follow-up clinical assessments, including lung function measurements, SPT, collection of biological samples (serum, plasma, and urine), and questionnaire data.[48] Subjects with available genotype data and lung function measured at the age of 8, 16, or 19 years were included in this study.

Genotyping and quality control analyses

Genotyping was performed using the Illumina Human610-Quad BeadChip (Illumina) platform. Low-quality SNPs and samples were discarded following the same criteria as for Ashford.[6]

Spirometry

Spirometry was performed at ages 8, 11, 16, and 20 years according to the ATS/ERS guidelines[7] using a Lilly pneumotachograph system with animated incentive software (Jaeger, Germany). For home visits, we used a flow turbine spirometer (Micro Medical, United Kingdom). Subjects were asked to inhale to TLC, then instructed to perform a forced expiration, through a mouthpiece, to RV. The test was repeated at intervals of 30 seconds until 3 technically acceptable traces were obtained. FEV_1 and FVC were recorded, and the data expressed as FEV_1 % predicted and FEV_1/FVC ratio. Short-acting β2-agonists were withheld for at least four, and long-acting for at least 24 hours before testing. Participants were symptom-free at the time of assessment.

PIAMA

Recruitment and study design

The Prevention and Incidence of Asthma and Mite Allergy (PIAMA) study is an ongoing population-based birth cohort. Pregnant women were recruited through antenatal clinics in the north, west, and center of The Netherlands between 1996 and 1997, resulting in 3,963 newborns included in the baseline study population. Questionnaires were completed by the parents during pregnancy, when the child was 3 months old, and then annually from 1 up to 8 years of age. Questionnaires were completed by the parents as well as by the participants themselves at ages 11, 14, and 17 years. Information on child and family characteristics, a wide range of environmental and lifestyle exposures, asthma, and other allergic and respiratory outcomes was collected. Lung function was measured at ages 8, 12, and 16 years using spirometry.[49,50]

Genotyping and quality control analyses

DNA was extracted from blood or buccal swabs collected at the age of 4 or 8 years. Samples were genotyped using four different platforms: Illumina Human610-Quad BeadChip, Illumina Human OmniExpress BeadChip, Infinium OmniExpressExome BeadChip, and Infinium Global Screening Array (Illumina). SNPs were harmonized by base pair position annotation to the GRCh37/hg19 build of the human genome, genetic variant identifier, and strand for each platform. Individuals were removed based on the following criteria: 1) sex-mismatch, 2) heterozygous outliers (deviate ± 3SD from the sample heterozygosity mean), 3) duplicated or highly related (IBD score <0·1875), 4) ethnic outliers from PCA plot. After QC procedures, 2,075 samples remained, and data from the four platforms were merged. Genetic variants with CR ≤95%, MAF ≤1%, and HWE $p<1\cdot10\times10^{-6}$ were filtered out.

Spirometry

Lung function was measured through spirometry using Jaeger pneumotachographs (Viasys Healthcare, Conshohocken, Pennsylvania, United States) at age 8, and EasyOne spirometers (ndd Medical Technologies Inc, Zurich, Switzerland) at age 12. Both Jaeger Masterscreen pneumotachographs (CareFusion, Yoba Linda, California, United States) and EasyOne spirometers were used at age 16. Comparison between the spirometers used at age 16 showed systematic differences, which were corrected using a regression calibration.[51] All measurements were performed following the recommendations of the ATS and ERS.[7] At least three acceptable maneuvers per subject were required. Test results that did not meet these criteria but which were obtained from technically flow-volume curves (differences between the largest and next largest values for FEV_1 and FVC ⩽200 mL; n=190 at age 12 and n=76 at age 16) were included as well.[52]

Rotterdam Study

Recruitment and study design

The Rotterdam Study is an ongoing prospective population-based cohort study initiated in 1990, randomly inviting inhabitants of Ommoord, a district near Rotterdam, aged 45 or older. All participants who met the minimum age criterium, lived or moved into the study district were invited to the cohort. A complete questionnaire was filled in by the participants, a clinical examination was undertaken, and blood samples were provided for plasma measurements. Lung function was assessed through spirometry in 2002 and repeated in subsequent follow-up visits. A subset of participants was followed up in two or three visits and others were examined only once. Further details of the cohort description have been included in previous publications.[53]

Therefore, subjects have been classified into three different groups based on the time point when the pulmonary function test was carried out: pulmonary function test (PFT) 1, PFT2, and PFT3. For the present study, only subjects with available genotype data aged 50 years and older have been selected (n=11,496). Of this sample, 5,723 individuals had at least one interpretable spirometry measurement. A fraction of Rotterdam Study participants (n=3,534) have been included in the GWAS of COPD susceptibility performed by Sakornsakolpat *et al.*.[46] Therefore, subjects from this cohort were excluded from the main analyses of the evaluation of the association of the PRS for airflow limitation with lung function outcomes and assigned as a sensitivity group. Thus, both sample overlap between the base and target datasets,[45] and bias selection in case of exclusion of those samples from the analyses would be avoided.

Genotyping and quality control analyses

DNA was sampled from most participants of the Rotterdam Study and genotyped using 550 K (Rotterdam Study PFT 1 and PFT 2; single and duo array format) and 610 K (Rotterdam Study PFT 3; quattro array format) Illumina arrays. Exclusions included a call rate<98%, Hardy-Weinberg p-value $<1\times10^{-6}$, and MAF <0.01%. A

total of 11,496 participants passed genotyping quality control. Around 500,000 SNPs have been genotyped in the Illumina GWAS dataset, and imputation was performed using the HRC 1.1 reference panel.

Spirometry

Pre-bronchodilator spirometry was performed by trained paramedical personnel and validated in compliance with American Thoracic Society/European Respiratory Society guidelines.[7] Spirometry was performed using a SpiroPro portable spirometer (Erich Jaeger, Hoechberg, Germany) from 2002 until 2008. From 2009 onward spirometry measurements were performed on a Master Screen PFT Pro (Care Fusion, Houten, the Netherlands). Study visits to the research center before 2002 did not include spirometry. Participants were asked to refrain from using any prescribed pulmonary medication before the study visit.[54]

SEATON

Recruitment and study design

The Study of Eczema and Asthma to Observe the Effects of Nutrition (SEATON) is an unselected birth cohort designed for the exploration of the association between maternal diet during pregnancy and asthma and allergic outcomes in childhood. A total of 2,000 healthy pregnant women attending an antenatal clinic in Aberdeen (United Kingdom), at a median of 12 weeks of gestation, were recruited and administered a questionnaire by a specialized interviewer. The atopic status was ascertained by a skin prick test (SPT). Thus, 1,924 children born between April 1998 and December 1999 were included prenatally and followed up by a self–completion questionnaire at the age of 15 years provided by post mail to record the presence of asthma and allergic diseases. Lung function measurements and SPT to common allergens were performed at 5, 10, and 15 years.[55] For the present study, only lung measurements for the follow-up visit at 10 and 15 years of age were evaluated.

Genotyping and quality control analyses

Samples were genotyped with Illumina Human610-Quad BeadChip (Illumina). The same QC criteria for the selection of genetic variants and samples described for Ashford were applied.[6]

Spirometry

Spirometry was assessed according to the ATS/ERS recommendations at 15 years of age.[7]

Calculation of a polygenic risk score for airflow limitation in different populations

Base dataset

The summary statistics of the largest GWAS of COPD susceptibility published to date[46] were selected as the base dataset for the PRS estimation in this study. The authors of this investigation evaluated the association of genetic variants across the genome with COPD in 257,811 adults of European ancestry included in the International COPD Genetics Consortium (ICGC)[56] and the UK Biobank.[57] Cases (n=35,735) were defined as COPD patients with evidence of moderate to severe airflow limitation according to modified Global Initiative for Chronic Obstructive Lung Disease (GOLD)[58] criteria: pre-bronchodilator FEV_1<80% of the predicted and $FEV_1/FVC<0{\cdot}7$. Controls showed normal spirometry parameters of lung function (n=222,076).[46] Subjects with a self-report of asthma were not excluded, as well as both smokers and non-smokers were included in the analyses. Nonetheless, sensitivity analyses performed by the authors suggested that the genetic association with COPD susceptibility was not driven by smoking or asthma statuses.[46] The authors identified the association of 82 independent SNPs with COPD as airflow limitation at the genome-wide significance level (p-value $\leq 5x10^{-8}$).[46] These were selected for the calculation of the PRS for airflow limitation in the present study.

Quality control analyses prior to the PRS calculation

QC procedures were applied on the 82 initially selected SNPs before the calculation of the individual PRS for airflow limitation in subjects with available genome-wide genotype data of each of the CADSET cohorts part of the target dataset.[31,45] The R software (version ≥3.6.0) was used for the analyses described below.[59] These were aimed to ensure the correct matching of variant information between the base and target datasets and ensure the avoidance of sample overlaps between both datasets as well. The standard guidelines by Choi *et al.*[45] were followed, applying the approach previously described,[31] except for minor modifications.

In the base dataset, the heritability of the 82 SNPs selected for the PRS estimation in the base dataset was checked in order to avoid leading to incorrect conclusions from the PRS estimates. These were reported by the authors to

explain up to 7·0% of the variance in COPD susceptibility on the liability scale, based on the prevalence of 10% for this disease.[46] PRS estimates are recommended to be calculated from genetic variants with $h^2 > 0{\cdot}05$;[45] thus, it could be assumed that these genetic variants explain a sufficient proportion of the phenotypic variation. Additionally, genetic variants without available effect alleles or sizes reported in the base dataset were excluded.

On the other hand, allele dosages for the selected variants were extracted from the imputation files for each of the cohorts included in the target dataset (**Table S1**). Various QC criteria were applied to ensure that the variant information from the base and target dataset were correctly matched to the same allele. First, chromosome positions and SNP identifiers were verified to be annotated in the same build of the human genome (GRCh37/hg19) in both datasets. Otherwise, the tool LiftOver[60] was used to convert them and standardize the genome build. Furthermore, the correspondence between the effect allele of the association with COPD susceptibility in the base and target dataset was evaluated. Thus, strand-ambiguous SNPs with complementary alternative alleles (C/G, G/C, A/T, or T/A) were excluded to avoid introducing potential errors pairing up alleles in the absence of information related to the chromosome strand used for genotyping. Multiallelic SNPs, duplicates, and insertions/deletions (INDELs) were also discarded. Moreover, allele and strand mismatches were identified, and the sign of the effect size of the association in the reference GWAS was inverted for genetic variants with switched alleles and the same or opposite strand to make certain that the effect size in the base dataset and allele dosage in each cohort part of the target dataset are referred to the same allele. Only common genetic variants with good imputation quality (MAF $\geq 0{\cdot}01$ and Rsq $\geq 0{\cdot}5$) were selected. The pairwise linkage disequilibrium (LD) among the selected 82 SNPs was assessed using European populations from the 1,000 Genomes Project (1KGP) reference panel utilizing the *LDpop* function implemented in the *LDlinkR* R package.[59,61] Thus, only independent SNPs ($R^2 < 0{\cdot}2$) were retained for subsequent analyses.

Alternative SNPs or proxies were investigated to replace the excluded variants that were not available among the imputation data or that did not satisfy any of the QC criteria. A set of SNPs in high LD ($R^2 > 0.7$) with each of the failing variants was obtained by using the *LDproxy* function implemented in the *LDlinkR* R package.[59,61] Allele dosages of proxies were extracted from the imputation files and the same QC procedures described above were applied. Additionally, the sign of the effect size of the association in the base dataset was inverted for those proxies whose effect allele in the target dataset was correlated to the non-effect allele of the corresponding original SNP that had failed any of the QC procedures or when the non-effect allele of the proxy was in high LD with the effect allele of the correlated SNP included in the original list. Thus, the effect size of the association of the original SNPs in the base dataset and the allele dosage of the proxy correspond to the same alleles. For each SNP that failed the QC, the proxy with the highest R^2 value that satisfied the QC criteria was selected for PRS estimation.

Additionally, part of the Lifelines cohort had been included in the GWAS of COPD susceptibility assigned as the base dataset[46] for the current study. Therefore, only subjects that had not previously been analyzed were selected for the estimation of the PRS for airflow limitation and subsequent evaluation of its association with lung function to avoid sample overlap between the base and target datasets, as recommended by standard PRS guidelines.[45]

PRS calculation

A weighted PRS for airflow limitation was independently derived per subject in each participating CADSET cohort using the R software.[59] This was calculated as the sum of the dosage of the effect allele of the genetic variants that passed QC criteria (from the original list and proxies) in the target dataset weighted by the effect size of the association between each SNP and COPD susceptibility in the base dataset. PRS estimates were scaled by their transformation into z-scores with a mean of 0 and standard deviation of 1 by applying the following formula: PRS – mean (PRS) / standard deviation (PRS).

Association testing of the PRS for airflow limitation with spirometry measurements in each study

The PRS for airflow limitation obtained was tested in association with lung function in participants with available genotype data, and thus the individual PRS, as well as spirometry measurements. According to the standard recommendations for the PRS calculation by Choi *et al.*,[45] the overlap between the samples included in the reference GWAS of COPD susceptibility and the CADSET cohorts was inspected to prevent potential inflation of the results of the association with lung function in the target dataset. Thus, subjects also included in the GWAS carried out by Sakornsakolpat *et al.*[46] were discarded from the main analyses.

Association analyses were independently conducted in each cohort, separately evaluating the association with spirometry measurements collected at each available time point in the case of longitudinal studies. In the only

participating cross-sectional study, Lifelines, subjects were classified into two groups based on age similarity (18-30 and 31-40 years), and the association testing was independently performed in each of them. The same approach was applied in the HUNT cohort since participants of different ages were simultaneously recruited, resulting in four different groups (20-30, 31-40, 41-50, and >50 years of age). Moreover, subjects from the Rotterdam Study were classified into three groups based on the time point when the pulmonary function test was conducted. No effects of the PRS for airflow limitation on the trend of lung function over time through the combination of measurements or individual trajectory groups were assessed. Our rationale for this was to reduce the complexity of the analyses, as well as to evaluate the potential variability in the contribution of the genetic factors of COPD susceptibility on lung function across different age groups.

The association with GLI z-scores of each pre-bronchodilator spirometry measurement (FEV_1, FVC, and FEV_1/FVC) was evaluated through linear regression models using the R software (version ≥3.6.0).[59] A regression model, hereafter basic association model, was first applied including two PCs of genetic ancestry as covariates in all cohorts, except for the INMA, $COPSAC_{2010}$ and $COPSAC_{2000}$, HUNT, and Generation R cohorts, where three, five, ten, and twenty PCs, respectively, were needed to allow sufficient control for population stratification. Association models were not adjusted by any PCs in the GINIplus/LISA South cohort due to high homogeneity in genetic ancestry among participants. Study-specific variables were also included as covariates such as the study group in GINIplus/LISA South; and the participation round and genotyping batch in HUNT.

Association results with spirometry measurements from each cohort were combined in an inverse-variance meta-analysis using the *metafor* R package.[59,62] Most of the participating studies had available repeated measurements of lung function taken at different time points, resulting in duplicated information from a great proportion of the participants. Therefore, results from each cohort and time point were classified into several groups based on age resemblance, similar to the approach previously applied.[63] This classification was designed to avoid data redundancy given by the inclusion of two or more time points per cohort into the same age group. In the case of duplicated studies within groups despite this classification, the time point with the smallest sample size was excluded from the meta-analysis. Thus, effect sizes of the association of the PRS for airflow limitation with FEV_1, FVC, and FEV_1/FVC z-scores obtained in each cohort were meta-analyzed per age group (**Table S3**). A random-effects model was applied in order to account for the heterogeneity across studies regardless of the significance of the Cochran Q-test and I^2 estimate. The significance of the association of the PRS was adjusted accounting for the false discovery rate (FDR). The Benjamini & Yekutieli method,[64] which is a conservative modification of the Benjamini & Hochberg method[65] and designed for all forms of dependency among tests, was applied across spirometry measurements per age group. Therefore, evidence of significant association was considered at q-value≤0·05. A meta-regression analysis was performed to evaluate the potential existence of a significant trend in the association effect size with spirometry measurements across age groups. A weighted least squares method was applied using the *metafor* R package.[59,62] Several linear and non-linear models were inspected, and the one that provided a better fit in terms of the log-likelihood and information criteria was selected. Evidence of a relationship between the age group and the effect size was considered in case of a significant test of moderators (p-value ≤0·05).

These results were validated in subjects older than 50 years of age from HUNT using the same approach. This was also independently conducted in the Rotterdam Study, whose participants from the extremes of the distribution of lung function had been included in the GWAS by Sakornsakolpat *et al.*.[46] Following the standard recommendations of avoiding sample overlap between the base and target datasets in the PRS calculation and the evaluation of its association with traits of interest,[45] Rotterdam Study participants were excluded from the main meta-analysis. Nonetheless, their complete exclusion from our analyses would have caused a bias in the sample selection and, subsequently in the effect of the association with spirometry measurements. Therefore, the Rotterdam Study's subjects were included in an additional validation in adults older than 50 years of age. Association analyses between the PRS for airflow limitation and lung function were separately conducted at each of the time points that participants were grouped on based on the follow-up visit when the pulmonary function test was conducted (PFT 1, PFT 2, and PFT 3).

The difference in FEV_1/FVC between the extremes of the distribution of PRS estimates divided into deciles was evaluated and quantified in one pediatric (BAMSE-W2) and one adult cohort (HUNT) as a representation of the participating studies. Linear regressions were separately performed in each age group applying the following model:

$$FEV_1/FVC\ (z\text{-}score) \sim Decile\ group + Covariates$$

This analysis was adjusted by the same covariates included in the basic regression model evaluating the association of the PRS with spirometry measurements. Therefore, linear regressions were adjusted by two PCs in BAMSE-W2, and by ten PCs, the participation round, and the genotyping batch in HUNT. Decile 1 was set as the reference group; thus, the FEV_1/FVC levels in not only the top decile (decile 10) but from decile 2 onwards

were compared to decile 1 (bottom decile). The magnitude of change in FEV_1/FVC z-score in decile 10 compared to decile 1 was calculated using the effect estimate for the intercept, the effect estimate for decile 10, and the decile group (decile 10) through the formula:

$$\textit{Change in } FEV_1/FVC \textit{ z-score} = \textit{Effect size}_{Intercept} + \textit{Effect size}_{Decile\ 10} * \textit{Decile } 10$$

Estimation of the variance in lung function explained by the PRS for airflow limitation

The performance of linear regression models evaluating the association of the PRS for airflow limitation with FEV_1, FVC, and FEV_1/FVC z-scores was estimated in different age groups through cross-validation using the *caret* R package.[59,66] This was independently carried out in the studies with the largest sample size per age group as a representation of the participating cohorts. The same covariates described above for the basic association model were included. For that, data from each of these cohorts were split into ten equally sized chunks; thus, the model was constructed in the first nine and the prediction in the tenth. The phenotypic variance in each of the spirometry measurements explained by the PRS for airflow limitation was derived in terms of R^2. Furthermore, the fit of each regression model was evaluated through the estimation of the root mean square error (RMSE) as an indicator of the average distance between the values of lung function predicted from the model and the actual ones in each cohort.

Sensitivity analyses

The potential effect modification of the association between the PRS for airflow limitation and spirometry measurements by active smoking was assessed in participants aged 18 years or older. Firstly, association testing with lung function at the available time points was carried out in each cohort using the same approach described for the basic association model but also adjusting by a binary variable related to active tobacco smoking status. This covariate was defined as the self-reported current consumption of cigarettes at the time of data collection. Secondly, linear regressions were conducted only in subjects with reported active smoking habits accounting for the duration and the number of packs of tobacco cigarettes. For that, a covariate related to "pack-years" was included in the basic association model. This was defined as the multiplication of the number of smoking years by the number of daily cigarettes and divided by 20 cigarettes often contained in a package.

Potential differences in the association between the PRS for airflow limitation and lung function by sex were explored by the stratification of the association testing into males and females per time point and cohort evaluated. The basic and smoking sensitivity association models were applied. Additional validation was carried out in overlapping cohorts with the base dataset.[46] Linear regressions were carried out for each of the spirometry measurements, adjusting by the covariates included in the basic association analyses, and additionally by active smoking status or pack-years in smokers. Results of each sensitivity analysis obtained in each cohort were meta-analyzed by age groups applying the approach described for the basic association model.

Furthermore, the potential effect of asthma on the link between the PRS for airflow limitation and lung function measurements was evaluated in the BAMSE cohort. First, individuals with asthma were excluded from the association testing between the PRS for airflow limitation and lung function to discern whether the inclusion of patients with asthma would have affected the association of the PRS for airflow limitation with spirometry measurements. Asthma was defined as a doctor's diagnosis and any symptoms with breathing difficulties or occasional or regular use of asthma medications in the last 12 months. Association analyses were separately carried out in each of the genotyping waves by applying the basic association model. Second, the association of the PRS for airflow limitation with asthma status was evaluated through logistic regressions to explore whether the genetic loci combined in the PRS for airflow limitation could be linked to asthma.

SUPPLEMENTARY RESULTS

Characteristics of the participating CADSET cohorts

A single individual PRS for airflow limitation was obtained for subjects with available genome-wide genotype data from each participating study, except for the Lifelines cohort, where age-specific scores were obtained due to the cross-sectional type of this cohort. Thus, the PRS for airflow limitation was independently estimated for

individuals included in the groups aged 18-30 (n=859) and 31-40 years (n=3,005). Moreover, analyses were separately conducted in each of the genotyping Waves of the BAMSE cohort (Wave 1, n=463; Wave 2, n=2,173) given methodological differences in the platform utilized for the genotype determination.

Association testing with lung function was separately conducted for each available time point, covering a vast majority of the lifespan spectrum after combining the information from all the contributing cohorts. The availability of spirometry measurements at at least one time point in addition to genetic data were inclusion criteria. Repeated participants' measurements collected at three follow-up visits were available for most of the cohorts (ALSPAC, BAMSE, $COPSAC_{2000}$, IoWBC, MAAS, PIAMA, and the Rotterdam Study). Nevertheless, other cohorts contributed with information on lung function levels measured at only one (Ashford, $COPSAC_{2010}$), two (Generation R, GINIplus/LISA North, GINIplus/LISA South, and SEATON), or six (INMA) different time points. In the HUNT and Lifelines cohorts, the association analyses were independently performed in each age group.

Spirometry measurements were predominantly available from preschool age to young adulthood across cohorts, with a mean age ranging from 4·5 ± 0·1 to 27·1 ± 2·6 years. Overall, pubertal individuals showed a poorer average lung function, with participants at the age of 15 years from the ALSPAC cohort presenting the lowest mean FEV_1 z-score (-0·7 ± 1·3) and FVC z-score (-0·9 ± 1·2). However, preschool participants also showed low FEV_1 (INMA, 4 years: -0·6 ± 1·2) and FVC z-scores (INMA, 4 years: -0·6 ± 1·3; GINIplus/LISA South, 6 years: -0·6 ± 0·9). Additionally, an obstructive phenotype evidenced by lower FEV_1/FVC z-score values was observed in older participants (IoWBC, 26 years: -0·7 ± 0·9). On the contrary, higher FEV_1 and FVC z-scores were found at the age of 8 years old in PIAMA (0·5 ± 0·9) and BAMSE (0·6 ± 0·9), respectively, as well as an increased mean FEV_1/FVC z-score in participants aged 6 years old (GINIplus/LISA South, 1·3 ± 0·8) and 10 years old (GINIplus/LISA North, 0·9 ± 1·0). The influence of genetic determinants of airflow limitation on lung function could also be explored in subjects from the HUNT cohort aged between 31 and 50 years, with validation in participants above 50 years of age from the same cohort (mean age 63·4 ± 8·9). Additional evaluation of the association with lung function was also conducted in the three time points from the Rotterdam Study: PFT 1 (50-98 years), n=5,722; PFT 2 (51-96 years), n=3,317; PFT 3 (70-100 years), n=741. It is important to highlight that the GLI equations had previously been found not to perform a good fit on the normalization of FEV_1, FVC, and FEV_1/FVC in several CADSET cohorts and time points, as described by Wang *et al.*.[63] Indeed, deviations from the established range (-0·4≤ mean z-score ≤0·4)[24] were detected, even though the standard deviation was approximately 1·0 in all time points.[63]

No major differences in sex were found, with nearly half of the participants being males in all the cohorts, except for the INMA cohort at the 18-year-old follow-up with a slightly lower proportion of males (35·6%). Among adults (≥18 years of age), 19-year-old subjects from the MAAS cohort showed the lowest proportion of active smokers (13·3%) together with individuals recruited by the Rotterdam Study (PFT 1, 50-98 years: 14·8%; PFT 2, 51-96 years: 10·4%; PFT 3, 70-100 years: 6·2%). No smoking-related information was available for IoWBC participants at the age of 26 years old. On the contrary, the highest proportions of subjects with reported active tobacco smoking habits were observed among HUNT and Lifelines participants (**Table 1**).

Calculation of a polygenic risk score for airflow limitation in different cohorts

The totality of the 82 independent genome-wide significant association signals (*p*-value $\leq 5x10^{-8}$) of susceptibility to adult COPD (**Table S2**) had available summary statistics in the base dataset,[46] including information related to the effect allele and size of the association. All genetic variants had been identified as independent association signals by the authors of the base dataset and confirmed in the cohorts that are part of the target dataset, showing no evidence of high pairwise LD (R^2<0·2). Among these, between 61 and 63 SNPs passed the QC criteria in the participating CADSET cohorts, except for PIAMA, where 52 SNPs remained after QC procedures (**Table S4**). Being strand-ambiguous or multiallelic SNPs were the most common reasons for exclusion across all participating cohorts. Genetic variants with low imputation quality (Rsq <0·5) or with evidence of deviation from the Hardy-Weinberg equilibrium ($p<1x10^{-6}$) were also discarded in some cohorts. Additionally, genetic variants with switched alleles and the same strands were detected, which were corrected by inverting the sign of the effect size of the association in the base dataset to guarantee that the variant information correctly matched between the base and target dataset based on the same allele. High-quality proxies (R^2 >0·7) replaced the SNPs that did not satisfy any of the QC conditions, except for genetic variants not available in the 1KGP reference panel (rs62375246, rs156394) which were discarded from subsequent analyses, as well as SNPs without any available proxies.

As a result, a total of 77-80 SNPs were included in the PRS calculation across participating cohorts, except for PIAMA, where only 69 SNPs could be included since no high-quality proxies that could replace some of the

SNPs that did not satisfy any of the QC conditions were available in this cohort (**Table S5**). A high overlap of genetic variants included was detected among studies. Even though variance in the number of genetic variants might result in differences in the PRS computed,[45] no evidence was found in BAMSE, where a high correlation (R^2 Pearson >0·8) between PRS estimates obtained using the whole set of SNPs and at least 70% of the total number of variants was found (**Table S6**). This suggests that the slight differences in the number of polymorphisms included in the PRS calculation among cohorts might have not caused substantial changes in the scores obtained. Indeed, high homogeneity was detected among participating cohorts after the normalization of the PRS estimates into z-scores (**Figures S1-S2**). Moreover, slightly broader ranges of the PRS for airflow limitation were detected in the HUNT cohort (**Figures S1**, **Figure S2I**), and the Rotterdam Study **Figures S1**, **Figure S2O**), which could likely be the result of the effect of larger sample sizes (**Table S7**).

Association testing with spirometry measurements

The PRS for adult airflow limitation was associated with lower lung function in subjects aged up to 50 years across the analyzed cohorts. Indeed, stronger evidence of association with the FEV_1/FVC z-score in terms of effect size and significance level was detected in each cohort compared to FEV_1 or FVC (**Table S8**). Association results from each time point and cohort were separately meta-analyzed in different groups based on age similarity. A total of seven age groups were defined: preschool age, 0-6 years (n=665); school age, 7-10 years (n=13,387); puberty, 11-15 years (n=9,323); post-puberty, 16-17 years (n=2,645); adulthood, 18-30 years (n=9,132); adulthood, 31-40 years (n=6,112); and adulthood, 41-50 years (n=4,142) (**Table S3**). The INMA and IoWBC cohorts had available data from several time points that would have been included in the same age group: INMA, 7 years (n=925) and INMA, 10 years (n=65) (school age); INMA, 11 years (n=792) and INMA, 14 years (n=188) (puberty); and IoWBC, 18 years (n=669) and IoWBC, 26 years (n=432) (adulthood, 18-30). Thus, only results from the time point with the largest sample size per cohort were included in the meta-analysis of these age groups to avoid duplicated cohort sample information.

After combining the association results of the PRS for airflow limitation with FEV_1, FVC, and FEV_1/FVC z-scores obtained in participants aged up to 50 years old from each cohort in an age-stratified meta-analysis, evidence of association with lower FEV_1 and FEV_1/FVC z-scores was found in age groups ranging from school age to adulthood (41-50 years). No major differences in the magnitude of the association effect were detected among studies either (**Figures S3**, **S6-S7**). Subjects at the top decile of the distribution of PRS estimates showed significantly lower mean lung function levels compared to individuals at the bottom decile (exemplified in BAMSE-W2 and HUNT cohorts; **Figure S5**). The FEV_1/FVC levels in the top decile (decile 10) were observed to be significantly different from the bottom decile (decile 1) in school age (β: -0·47, 95% Confidence Interval (CI): -0·69, -0·25, *p*-value=$2{\cdot}81\times10^{-5}$, *q*-value=$8{\cdot}23\times10^{-4}$), post-puberty (β: -0·58, 95% CI: -0·82, -0·35, *p*-value=$1{\cdot}75\times10^{-6}$, *q*-value=$2{\cdot}56\times10^{-5}$), and adulthood, 18-30 years (β: -0·52, 95% CI: -0·75, -0·28, *p*-value=$2{\cdot}35\times10^{-5}$, *q*-value=$6{\cdot}88\times10^{-4}$) in BAMSE-W2 (**Figures S5A-S5C**). The following results were observed for the intercept of the regression per age group: school age, β: -0·10, 95% CI: -0·26, 0·06, *p*-value=0·211, *q*-value=0·773; post-puberty, β: 0·07, 95% CI: -0·10, 0·24, *p*-value=0·427, *q*-value=1; adulthood (18-30 years), β: -0·10, 95% CI: -0·27, 0·07, *p*-value=0·255, *q*-value=0·934). A decrease of five (school age and adulthood, 18-30 years) or six times (post-puberty) in FEV_1/FVC z-score in the top decile compared to the bottom decile was found (**Figure S4A**, **Figures S5A-S5C**). Similar results were observed in adults aged up to 50 years participating in the HUNT cohort (**Figures S5D-S5F**), with significant differences in FEV_1/FVC between the extremes of the distribution of PRS estimates: 18-30 years, β: -0·67, 95% CI: -0·82, -0·52, *p*-value=$3{\cdot}59\times10^{-19}$, *q*-value=$1{\cdot}05\times10^{-17}$; 31-40 years, β: -0·66, 95% CI: -0·81, -0·51, *p*-value=$1{\cdot}62\times10^{-17}$, *q*-value=$4{\cdot}74\times10^{-16}$; 41-50 years, β: -0·61, 95% CI: -0·75, -0·47, *p*-value=$1{\cdot}22\times10^{-17}$, *q*-value=$3{\cdot}57\times10^{-16}$. FEV_1/FVC z-scores were between six and eight times lower in the top PRS decile compared to the bottom extreme of the distribution, considering the effect size observed in this decile and the intercept of the regression model (18-30 years: β: 0·08, 95% CI: -1·61, 1·77, *p*-value=0·925; *q*-value=1; 31-40 years: β: -1·16, 95% CI: -2·78, 0·45, *p*-value=0·158; *q*-value=0·514; 41-50 years: β: -0·22, 95% CI: -1·71, 1·27, *p*-value=0·775; *q*-value=1) (**Figure S4B**, **Figures S5D-S5F**).

The meta-analysis of the association results with lung function outcomes was also carried out by replacing the time points discarded from those cohorts with duplicated data within the school age (INMA, 10 years), puberty (INMA, 14 years), and adulthood, 18-30 groups (IoWBC, 26 years). Nonetheless, similar results were observed between the main and alternative composition of the aforementioned age groups, which was confirmed by a high correlation in the effect size (R^2=1) and significance level (R^2=0.97) obtained across age groups. These results evidence the absence of any selection bias in the definition of age groups (**Table S9**, **Figure S8**).

The association between genetic factors of airflow limitation and lung function z-scores was validated in 9,027 older subjects (>50 years old) from the HUNT cohort. Evidence of association with lower FEV_1 (β: -0·16, 95%

CI: -0·19, -0·13, q-value=1·60x10^{-30}) and FEV_1/FVC (β: -0·17, 95% CI: -0·19, -0·14, q-value=1·09x10^{-38}) was also found. Indeed, a decrease of the order of approximately six times in FEV_1/FVC levels was observed in HUNT participants from the top PRS decile compared to the bottom decile, accounting for the effect size found in the top decile (β: -0·64, 95% CI: -0·75, -0·53, p-value=8·20x10^{-29}; q-value=2·40x10^{-27}) and the intercept (β: 0·38, 95% CI: -0·85, 1·61, p-value=0·550; q-value=1) of the linear regression model assessing the differences in lung function between the extremes of the PRS distribution (**Figure S4B**, **Figure S5G**). Significant association with FVC was also detected in this age group (β: -0·07, 95% CI: -0·09, -0·05, q-value=1·06x10^{-9}).

Evidence of significant association of the PRS for airflow limitation with lower FEV_1 was also observed in each of the time points of the Rotterdam Study evaluated by applying the basic association model: PFT 1 (50-98 years), β: -0·15, 95% CI: -0·18, -0·12, q-value=5·89x10^{-20}; PFT 2 (51-96 years), β: -0·14, 95% CI: -0·17, -0·10, q-value=9·19x10^{-12}; PFT 3 (70-100 years), β: -0·17, 95% CI: -0·25, -0·09, q-value=1·09x10^{-4} (**Table S10**). Significant association with FEV_1/FVC was found as well: PFT 1 (50-98 years), β: -0·14, 95% CI: -0·17, -0·11, q-value=2·38x10^{-23}; PFT 2 (51-96 years), β: -0·15, 95% CI: -0·19, -0·12, q-value=5·31x10^{-21}; PFT 3 (70-100 years), β: -0·19, 95% CI: -0·26, -0·13, q-value=9·41x10^{-8} (**Table S10**). These results validated the findings obtained in younger individuals. Consistently with the results found in adults aged 31-40 and 41-50 years, as well as HUNT participants from the same age group (>50 years of age), the PRS for airflow limitation was also associated with the FVC z-score in the Rotterdam Study (**Table S10**).

Estimation of the variance in lung function explained by the PRS for airflow limitation across the lifespan

The evaluation of the performance of linear regression models, testing the association of the PRS for airflow limitation with lung function across different age groups, revealed that the PRS explained a limited proportion of the total variance in lung function. Nonetheless, a larger proportion of the variation in FEV_1/FVC and FEV_1 than FVC was found to be explained by the PRS for airflow limitation in most of the age groups. These findings are consistent with the meta-analysis results, suggesting a stronger influence of genetic determinants of COPD on FEV_1/FVC and FEV_1. Despite slight differences in the average percentage of FEV_1 explained by the PRS, ranging from 1·4% in puberty to 6·3% in school age, no evidence of a trend across age groups was observed. Nonetheless, the PRS explained a lower proportion of FEV_1 in 8-year-old and 15-year-old ALSPAC participants (R^2=0·005, and R^2=0·004, respectively) than in subjects from other cohorts. Similar findings were obtained for FEV_1/FVC, with the PRS for airflow limitation explaining between 1·5% and 6·5% in school-age children (**Table S11**). Lower estimates were found in ALSPAC's subjects (8 years of age: R^2=0·015; 15 years of age: R^2=0·019).

Sensitivity analyses

Active smoking habits

Sensitivity analyses evaluating the effect of active smoking suggested that the association of the PRS for airflow limitation with z-scores of pre-bronchodilator lung function outcomes is not driven by this risk factor. The same approach described for the basic association model was applied; thus, association analyses were independently carried out in each of the cohorts with available spirometry measurements in adulthood (≥18 years of age), including only subjects with available information about smoking habits (**Table S12**). Then, an age-stratified meta-analysis of the association results obtained for the three groups of adults up to 50 years of age was conducted: 18-30 years (n=8,264), 31-40 years (n=6,015), and 41-50 years (n=4,103).

The results obtained were similar to the ones applying the basic association model in adults (**Figure S9**). No major changes were found after including a covariate related to smoking status in the regression models. Indeed, a higher genetic risk of COPD remained significantly associated with lower FEV_1 [adulthood (18-30 years): β: -0·11, 95% CI: -0·14, -0·09, q-value=3·58x10^{-20}; adulthood (31-40 years): β: -0·13, 95% CI: -0·16, -0·10, q-value=2·08x10^{-13}; adulthood (41-50 years): β: -0·16, 95% CI: -0·20, -0·13, q-value=2·75x10^{-19}] and FEV_1/FVC [adulthood (18-30 years): β: -0·16, 95% CI: -0·18, -0·13, q-value=3·45x10^{-42}; adulthood (31-40 years): β: -0·16, 95% CI: -0·19, -0·14, q-value=5·56x10^{-42}; adulthood (41-50 years): β: -0·16, 95% CI: -0·19, -0·13, q-value=2·01x10^{-24}] in the three adulthood groups. Similarly, the PRS for airflow limitation was not associated with FVC in subjects aged between 18 and 30 (β: -0·01, 95% CI: -0·03, 0·01, q-value=1), and 31-40 years (β: -0·03, 95% CI: -0·05, 0·00, q-value= 0·066), but it was significant in the 41-50 year age group (β: -0·06, 95% CI: -0·09, -0·03, q-value=6·16x10^{-5}).

The significance level of the association with spirometry measurements accounting for the tobacco pack-years mostly decreased in the three adulthood groups inspected (up to 50 years of age). This might be explained by the

restrictive inclusion criteria for these analyses performed exclusively in participants with active smoking habits, as well as the non-availability of the necessary information to define a variable related to tobacco pack-years for most cohorts, subsequently reducing the sample size of the adulthood groups up to 50 years of age: 18-30 years (n=1,417), 31-40 years (n=1,734), and 41-50 years (n=1,392). However, no substantial differences in the effect size were detected (**Table S13**, **Figure S9**). Interestingly, the weak association with FVC that had been found in adults aged 31-40 (β: -0.03, 95% CI: -0.05, 0.00, *q*-value=0.043) and 41-50 years (β: -0.07, 95% CI: -0.10, -0.04, *q*-value=2.24x10^{-5}) after carrying out a meta-analysis of results from the basic association model of each cohort did not remain significant when adjusting by tobacco pack-years [adulthood (31-40 years): β: -0.03, 95% CI: -0.08, 0.01, *q*-value=0.268; adulthood (41-50 years): β: 0.00, 95% CI: -0.05, 0.05, *q*-value=1)] (**Figure S9**).

The robustness of these results was validated in 8,784 and 2,205 adults older than 50 years of age participating in the HUNT cohort through the inclusion of smoking status and tobacco pack-years as covariates, respectively, where a similar magnitude of the association effect was detected compared to younger subjects. The association results were almost identical when taking into account for smoking status (FEV$_1$: β: -0.16, 95% CI: -0.19, -0.13, *q*-value=1.57x10^{-30}; FVC: β: -0.07, 95% CI: -0.09, -0.05, *q*-value=1.79x10^{-9}; FEV$_1$/FVC: β: -0.17, 95% CI: -0.19, -0.14, *q*-value=2.08x10^{-39}) or pack-years, although less significant in the latter model (FEV$_1$: β: -0.19, 95% CI: -0.24, -0.14, *q*-value=1.95x10^{-12}; FVC: β: -0.08, 95% CI: -0.13, -0.04, *q*-value=4.52x10^{-4}; FEV$_1$/FVC: β: -0.21, 95% CI: -0.26, -0.16, *q*-value=1.65x10^{-15}).

Sex

The association results from each cohort were combined in an age-stratified meta-analysis separately in males (preschool age, n=340; school age, n=6,714; puberty, n=4,516; post-puberty, n=1,259; adulthood, 18-30 years, n=3,936; adulthood, 31-40 years, n=2,631; adulthood, 41-50 years, n=1,904), and females (preschool age, n=325; school age, n=6,673; puberty, n=4,807; post-puberty, n=1,386; adulthood, 18-30 years, n=5,196; adulthood, 31-40 years, n=3,481; adulthood, 41-50 years, n=2,238) (**Table S14**).

The PRS for airflow limitation remained significantly associated with lower FEV$_1$ and FEV$_1$/FVC after stratifying the analyses into males and females (**Table S14**). However, the link between the PRS and FEV$_1$ was interestingly found not to be significant in post-pubertal females (16-17 years of age) (β:-0.08, 95% CI: -0.16, 0.01, *q*-value=0.184) in contrast to males (β:-0.13, 95% CI: -0.18, -0.08, *q*-value=3.69x10^{-6}), and the results from both female and male strata and the same age group (β: -0.11, 95% CI: -0.15, -0.08, *q*-value=2.83x10^{-9}). Equivalently to the findings from analyzing the whole set of individuals, no association with FVC was observed in males or females between school age and adulthood (18-30 years), as well as adults aged 31-40 years. However, it was significantly associated in both males and females from the adulthood, 41-50 years group (males: β: -0.06, 95% CI: -0.11, -0.02, *q*-value=9.70x10^{-3}; females: β: -0.07, 95% CI: -0.11, -0.03, *q*-value=1.25x10^{-3}) (**Figure S10**).

On the other hand, the adjustment by smoking status caused almost no changes in the effect size or significance level of the association with lung function in adults when males (**Figure S11A**) and females (**Figure S11B**) were independently analyzed compared to the basic association model stratified by sex. The inclusion of a tobacco pack-year-related variable in the association models in smokers did not substantially change the magnitude of the association effect with lung function either (**Figure S11A**, **Figure S11B**). Compared to the whole set of individuals regardless of sex, consistent results of the sensitivity analyses accounting for smoking status (**Figure S12A**) and pack-years (**Figure S12B**) were detected in males and females.

In adults aged older than 50 years from the HUNT cohort, the stratification by sex of the basic regression model did not substantially affect the association between the PRS for airflow limitation and spirometry measurements, except for a slightly larger effect size and significance level of the association in males (FEV$_1$: β: -0.21, 95% CI: -0.25, -0.17, *q*-value= 2.75x10^{-23}; FVC: β: -0.08, 95% CI: -0.12, -0.05, *p*-value=4.60x10^{-7} *q*-value=8.43x10^{-7}; FEV$_1$/FVC: β: -0.22, 95% CI: -0.26, -0.18, *q*-value=8.86x10^{-30}) than in females (FEV$_1$: β: -0.12, 95% CI: -0.16, -0.09, *q*-value=1.71x10^{-10}; FVC: β: -0.06, 95% CI: -0.09, -0.03, *q*-value=2.59x10^{-4}; FEV$_1$/FVC: β: -0.12, 95% CI: -0.15, -0.09, *q*-value=2.39x10^{-12}). Smoking-related variables were not found to drive the association with lung function in any sex stratum from this age group either (**Table S15**). In PFT 1 (50-98 years) from the Rotterdam Study, females interestingly showed a more significant association of the PRS for airflow limitation with spirometry measurements compared to males, even though no major differences in the magnitude of the effect were found. Remarkably, the effect size and significance of the association with FEV$_1$, FVC, and FEV$_1$/FVC was larger in males included in PFT 3 (70-100 years) compared to females (**Table S16**). The negative direction of the effect and significance level of the association were preserved after including the smoking status. Nonetheless, no strong evidence of association with lung function was observed in males or females when adjusting by tobacco pack-years in smokers, which might be explained by the reduced sample size. Consistent results were found after stratifying by sex (**Table S16**).

SUPPLEMENTARY REFERENCES

1. Harris PA, Taylor R, Thielke R, Payne J, Gonzalez N, Conde JG. Research electronic data capture (REDCap)-A metadata-driven methodology and workflow process for providing translational research informatics support. *J Biomed Inform* 2009;**42**(2).
2. Fraser A, Macdonald-Wallis C, Tilling K, et al. Cohort Profile: the Avon Longitudinal Study of Parents and Children: ALSPAC mothers cohort. *Int J Epidemiol* 2013;**42**(1):97–110.
3. Boyd A, Golding J, Macleod J, et al. Cohort Profile: the 'children of the 90s'--the index offspring of the Avon Longitudinal Study of Parents and Children. *Int J Epidemiol* 2013;**42**(1):111–27.
4. Haag K, Fraser A, Hiller R, Seedat S, Zimmerman A, Halligan SL. The emergence of sex differences in PTSD symptoms across development: Evidence from the ALSPAC cohort. *Psychol Med* 2020;**50**(10):1755–60.
5. Northstone K, Lewcock M, Groom A, et al. The Avon Longitudinal Study of Parents and Children (ALSPAC): an update on the enrolled sample of index children in 2019. *Wellcome Open Res* 2019;**4**:51.
6. Granell R, Curtin JA, Haider S, et al. A meta-analysis of genome-wide association studies of childhood wheezing phenotypes identifies ANXA1 as a susceptibility locus for persistent wheezing. *Elife* 2023;**12**:e84315.
7. Miller MR, Hankinson J, Brusasco V, et al. Standardisation of spirometry. *Eur Respir J* 2005;**26**(2):319–38.
8. Wickman M, Kull I, Pershagen G, Nordvall SL. The BAMSE project: presentation of a prospective longitudinal birth cohort study. *Pediatr Allergy Immunol* 2002;**13**(s15):11–3.
9. Ostblom E, Lilja G, Pershagen G, van Hage M, Wickman M. Phenotypes of food hypersensitivity and development of allergic diseases during the first 8 years of life. *Clin Exp Allergy* 2008;**38**(8):1325–32.
10. Kull I, Melen E, Alm J, et al. Breast-feeding in relation to asthma, lung function, and sensitization in young schoolchildren. *J Allergy Clin Immunol* 2010;**125**(5):1013–9.
11. Hallberg J, Ballardini N, Almqvist C, et al. Impact of IgE sensitization and rhinitis on inflammatory biomarkers and lung function in adolescents with and without asthma. *Pediatr Allergy Immunol* 2019;**30**(1):74–80.
12. Melén E, Bergström A, Kull I, et al. Male sex is strongly associated with IgE-sensitization to airborne but not food allergens: Results up to age 24 years from the BAMSE birth cohort. *Clin Transl Allergy* 2020;**10**(1):15.
13. Odling M, Andersson N, Hallberg J, et al. A Gap Between Asthma Guidelines and Management for Adolescents and Young Adults. *J Allergy Clin Immunol Pr* 2020;**8**(9):3056-3065 e2.
14. Purcell S, Neale B, Todd-Brown K, et al. PLINK: a tool set for whole-genome association and population-based linkage analyses. *Am J Hum Genet* 2007;**81**(3):559–75.
15. Chang CC, Chow CC, Tellier LCAM, Vattikuti S, Purcell SM, Lee JJ. Second-generation PLINK: Rising to the challenge of larger and richer datasets. *Gigascience* 2015;**4**(1):7.
16. Abecasis GR, Auton A, Brooks LD, et al. An integrated map of genetic variation from 1,092 human genomes. *Nature* 2012;**491**(7422):56–65.
17. Hallberg J, Thunqvist P, Schultz ES, et al. Asthma phenotypes and lung function up to 16 years of age - The BAMSE cohort. *Allergy Eur J Allergy Clin Immunol* 2015;**70**(6):667–73.
18. Bisgaard H. The Copenhagen Prospective Study on Asthma in Childhood (COPSAC): Design, rationale, and baseline data from a longitudinal birth cohort study. *Ann Allergy, Asthma Immunol* 2004;**93**(4):381–9.
19. Igartua C, Myers RA, Mathias RA, et al. Ethnic-specific associations of rare and low-frequency DNA sequence variants with asthma. *Nat Commun* 2015;**6**:5965.
20. Vinding RK, Stokholm J, Chawes BLK, Bisgaard H. Blood lipid levels associate with childhood asthma, airway obstruction, bronchial hyperresponsiveness, and aeroallergen sensitization. *J Allergy Clin Immunol* 2016;**137**(1):68-74.e4.
21. Jaddoe VWV, Van Duijn CM, Franco OH, et al. The generation r study: Design and cohort update 2012. *Eur J Epidemiol* 2012;**27**(9).
22. Kooijman MN, Kruithof CJ, van Duijn CM, et al. The Generation R Study: design and cohort update 2017. *Eur J Epidemiol* 2016;**31**(12).
23. Graham BL, Steenbruggen I, Barjaktarevic IZ, et al. Standardization of spirometry 2019 update an official American Thoracic Society and European Respiratory Society technical statement. *Am J Respir Crit*

Care Med 2019;**200**(8):E70–88.

24. Quanjer PH, Stocks J, Cole TJ, Hall GL, Stanojevic S. Influence of secular trends and sample size on reference equations for lung function tests. *Eur Respir J* 2011;**37**(3):658–64.
25. Heinrich J, Bolte G, Hölscher B, et al. Allergens and endotoxin on mothers' mattresses and total immunoglobulin E in cord blood of neonates. *Eur Respir J* 2002;**20**(3):617–23.
26. Zutavern A, Brockow I, Schaaf B, et al. Timing of solid food introduction in relation to atopic dermatitis and atopic sensitization: Results from a prospective birth cohort study. *Pediatrics* 2006;**117**(2):401–11.
27. Berg A V., Krämer U, Link E, et al. Impact of early feeding on childhood eczema: Development after nutritional intervention compared with the natural course - The GINIplus study up to the age of 6 years. *Clin Exp Allergy* 2010;**40**(4):627–36.
28. Heinrich J, Brüske I, Cramer C, et al. GINIplus and LISAplus. Design and selected results of two German birth cohorts about natural course of atopic diseases and its determinants. *Allergol Sel* 2017;**1**(1):85–95.
29. Reed E, Nunez S, Kulp D, Qian J, Reilly MP, Foulkes AS. A guide to genome-wide association analysis and post-analytic interrogation. *Stat Med* 2015;**34**(28):3769–92.
30. Fuertes E, Bracher J, Flexeder C, et al. Long-term air pollution exposure and lung function in 15 year-old adolescents living in an urban and rural area in Germany: The GINIplus and LISAplus cohorts. *Int J Hyg Environ Health* 2015;**218**(7):656–65.
31. Kilanowski A, Thiering E, Wang G, et al. Allergic disease trajectories up to adolescence: Characteristics, early-life, and genetic determinants. *Allergy* 2023;**78**(3):836–50.
32. Holmen J, Midthjell K, Forsén L, Skjerve K, Gorseth M, Oseland A. A health survey in Nord-Trøndelag 1984-86. Participation and comparison of attendants and non-attendants [article in Norwegian]. *Tidsskr den Nor laegeforening* 1990;**110**(15):1973–7.
33. Krokstad S, Langhammer A, Hveem K, et al. Cohort profile: The HUNT study, Norway. *Int J Epidemiol* 2013;**42**(4):968–77.
34. Holmen J, Midthjell K, Krüger Ø, et al. The Nord-Trøndelag Health Study 1995-97 (HUNT 2): Objectives, contents, methods and participation. *Nor Epidemiol* 2003;**13**(1):19–32.
35. Åsvold BO, Langhammer A, Rehn TA, et al. Cohort Profile Update: The HUNT Study, Norway. *Int J Epidemiol* 2023;**52**(1):e80–91.
36. Brumpton BM, Graham S, Surakka I, et al. The HUNT study: A population-based cohort for genetic research. *Cell Genomics* 2022;**2**(10):12989.
37. Langhammer A, Johnsen R, Gulsvik A, Holmen TL, Bjermer L. Forced spirometry reference values for Norwegian adults: The bronchial obstruction in nord-trøndelag study. *Eur Respir J* 2001;**18**(5):770–9.
38. Langhammer A, Johannessen A, Holmen TL, et al. Global lung function initiative 2012 reference equations for spirometry in the Norwegian population. *Eur Respir J* 2016;**48**(6):1602–11.
39. Guxens M, Ballester F, Espada M, et al. Cohort profile: The INMA-INfancia y Medio Ambiente-(environment and childhood) project. *Int J Epidemiol* 2012;**41**(4):930–40.
40. Bosch de Basea M, Carsin AE, Abellan A, et al. Gestational phthalate exposure and lung function during childhood: A prospective population-based study. *Environ Pollut* 2022;**312**:119833.
41. Arshad SH, Holloway JW, Karmaus W, et al. Cohort profile: The isle of wight whole population birth cohort (ioWBC). *Int J Epidemiol* 2018;**47**(4):1043-1044I.
42. Kurukulaaratchy RJ, Fenn M, Twiselton R, Matthews S, Arshad SH. The prevalence of asthma and wheezing illnesses amongst 10-year-old schoolchildren. *Respir Med* 2002;**96**(3):163–9.
43. Kurukulaaratchy RJ, Fenn MH, Waterhouse LM, Matthews SM, Holgate ST, Arshad SH. Characterization of wheezing phenotypes in the first 10 years of life. *Clin Exp Allergy* 2003;**33**(5):573–8.
44. Scholtens S, Smidt N, Swertz MA, et al. Cohort Profile: LifeLines, a three-generation cohort study and biobank. *Int J Epidemiol* 2015;**44**(4):1172–80.
45. Choi SW, Mak TSH, O'Reilly PF. Tutorial: a guide to performing polygenic risk score analyses. *Nat Protoc* 2020;**15**(9):2759–72.
46. Sakornsakolpat P, Prokopenko D, Lamontagne M, et al. Genetic landscape of chronic obstructive pulmonary disease identifies heterogeneous cell-type and phenotype associations. *Nat Genet* 2019;**51**(3):494–505.
47. Faruque MO, Vonk JM, Kromhout H, Vermeulen R, Bültmann U, Boezen HM. Airborne Occupational Exposures and Lung Function in the Lifelines Cohort Study. *Ann Am Thorac Soc* 2021;**18**(1):60–7.
48. Custovic A, Simpson BM, Murray CS, Lowe L, Woodcock A. The National Asthma Campaign Manchester

Asthma and Allergy Study. *Pediatr Allergy Immunol Suppl* 2002;**13**(15):32–7.

49. Brunekreef B, Smit J, De Jongste J, et al. The Prevention and Incidence of Asthma and Mite Allergy (PIAMA) birth cohort study: Design and first results. *Pediatr Allergy Immunol Suppl* 2002;**13**(15):55–60.
50. Wijga AH, Kerkhof M, Gehring U, et al. Cohort profile: The prevention and incidence of asthma and mite allergy (PIAMA) birth cohort. *Int J Epidemiol* 2014;**43**(2):527–35.
51. Milanzi EB, Koppelman GH, Oldenwening M, et al. Considerations in the use of different spirometers in epidemiological studies. *Environ Heal A Glob Access Sci Source* 2019;**18**(1).
52. Gehring U, Milanzi EB, Koppelman GH, et al. Air pollution exposure and lung function until age 16 years: The PIAMA birth cohort study. *Eur Respir J* 2018;**52**(3):1800218.
53. Ikram MA, Brusselle GGO, Murad SD, et al. The Rotterdam Study: 2018 update on objectives, design and main results. *Eur J Epidemiol* 2017;**32**(9):807–50.
54. Loth DW, Brusselle GG, Lahousse L, Hofman A, Leufkens HGM, Stricker BH. β-adrenoceptor blockers and pulmonary function in the general population: The Rotterdam Study. *Br J Clin Pharmacol* 2014;**77**(1):190–200.
55. Cullinan P, MacNeill SJ, Harris JM, et al. Early allergen exposure, skin prick responses, and atopic wheeze at age 5 in English children: A cohort study. *Thorax* 2004;**59**(10):855–61.
56. Hobbs BD, De Jong K, Lamontagne M, et al. Genetic loci associated with chronic obstructive pulmonary disease overlap with loci for lung function and pulmonary fibrosis. *Nat Genet* 2017;**49**(3):426–32.
57. Sudlow C, Gallacher J, Allen N, et al. UK Biobank: An Open Access Resource for Identifying the Causes of a Wide Range of Complex Diseases of Middle and Old Age. *PLoS Med* 2015;**12**(3):e1001779.
58. Vogelmeier CF, Criner GJ, Martinez FJ, et al. Global strategy for the diagnosis, management, and prevention of chronic obstructive lung disease 2017 report. *Am J Respir Crit Care Med* 2017;**195**(5):557–82.
59. R Development Core Team. R: A language and Environment for Statistical. Vienna, Austria: R Foundation for Statistical Computing; 2013. http://www.R-project.org/.
60. Hinrichs AS, Karolchik D, Baertsch R, et al. The UCSC Genome Browser Database: update 2006. *Nucleic Acids Res* 2006;**34**(Database issue).
61. Myers TA, Chanock SJ, Machiela MJ. LDlinkR: An R Package for Rapidly Calculating Linkage Disequilibrium Statistics in Diverse Populations. *Front Genet* 2020;**11**:157.
62. W V. Conducting meta-analyses in R with the metafor package. *J Stat Softw* 2010;**36**(3):1–48.
63. Wang G, Hallberg J, Charalampopoulos D, et al. Spirometric phenotypes from early childhood to young adulthood: a Chronic Airway Disease Early Stratification study. *ERJ Open Res* 2021;**7**(4):00457–2021.
64. Benjamini Y, Yekutieli D. The control of the false discovery rate in multiple testing under dependency. *Ann Stat* 2001;**29**(4):1165–88.
65. Benjamini Y, Hochberg Y. Controlling the False Discovery Rate: A Practical and Powerful Approach to Multiple Testing. *J R Stat Soc Ser B* 1995;**57**(1):289–300.
66. Max Kuhn. Contributions from Jed Wing, Steve Weston, Andre Williams Chris Keefer, Allan Engelhardt, Tony Cooper, Zachary Mayer, Brenton Kenkel, the R Core Team, Michael Benesty, Reynald Lescarbeau A, Ziem, Luca Scrucca YT and CC. caret: Classification and Regression Training. R package version 6.0-71. 2016.

SUPPLEMENTARY FIGURE LEGENDS

Figure S1. Box plot of scaled estimates of the PRS for airflow limitation in each of the participating CADSET cohorts. The *y*-axis shows the z-scores of the PRS for airflow limitation calculated in each cohort (*x*-axis). Lower values in the *y*-axis indicate a low genetic risk of airflow limitation, in contrast to high PRS estimates. Color-coded boxes based on the cohort show the inter-quartile range. The thick horizontal line at each box displays the median of the distribution of normalized PRS estimates. Whiskers extending vertically from the boxes indicate the minimum and maximum values. The calculation of the PRS for airflow limitation was conducted in the complete set of individuals with available genome-wide genotype data, except for the BAMSE cohort, where it was estimated separately in each genotyping Wave. The PRS was also independently obtained in two groups of similar age in Lifelines (18-30 and 31-40 years). CADSET: Chronic Airway Diseases Early Stratification; PRS: polygenic risk score; W1: Genotyping Wave 1; W2: Genotyping Wave 2.

Figure S2. Distribution of normalized estimates of the PRS for susceptibility to airflow limitation calculated in each of the participating CADSET cohorts. The transformed values of the PRS in the form of z-scores obtained for the complete set of individuals with available genome-wide genotype data from each cohort are represented on the *x*-axis. Higher values of the PRS in the *x*-axis indicate a higher genetic risk for COPD. The mean is shown by the dashed blue line. Exceptionally, the PRS calculation was separately conducted in each of the genotyping Waves of the BAMSE cohort, as well as in two age groups of the Lifelines cohort. Figures S1A-P show the density plot of normalized PRS estimates for each cohort: A) ALSPAC (n=8,943); B) Ashford (n=348); C) BAMSE-W1 (n=463), and BAMSE-W2 (n=2,173); D) $COPSAC_{2000}$ (n=358); E) $COPSAC_{2010}$ (n=618); F) Generation R (n=5,756); G) GINIplus/LISA North (n=792); H) GINIplus/LISA South (n=1,511); I) HUNT (n=69,717); J) INMA (n=2,034); K) IoWBC (n=956); L) Lifelines, 18-30 years (n=859), and Lifelines, 31-40 years (n=3,005); M) MAAS (n=852); N) PIAMA (n=1,526); O) Rotterdam Study (n=11,496); P) SEATON (n=552). COPD: chronic obstructive pulmonary disease; PRS: polygenic risk score; W1: Genotyping Wave 1; W2: Genotyping Wave 2.

Figure S3. Forest plot of the results of the evaluation of the association between the PRS for airflow limitation and FEV_1/FVC z-scores from the age-stratified meta-analysis and each separate cohort (up to 50 years of age). The association effect (β estimates) and 95% Confidence Intervals (95% CI) are represented for each cohort and time point included in each age group by blue boxes and dash lines (*x*-axis). The effect size obtained from the meta-analysis (random-effects model) of each group is shown by a red diamond as the change in z-score of lung function per one z-score increase in the PRS, except for the adulthood group (41-50 years of age), where only association results from the HUNT cohort are shown due to the absence of more cohorts within that age range. Results are shown for the basic association model, adjusted by Principal Components of genetic ancestry and any cohort-specific covariates. Details of the number of cohorts, sample size, and association results are provided for each cohort and age group. The *q*-value represents the adjusted *p*-value accounting for the false discovery rate. FEV_1: forced expiratory volume in one second; FVC: forced vital capacity; PRS: polygenic risk score; W1: Genotyping Wave 1; W2: Genotyping Wave 2.

Figure S4. Decile plot of the mean FEV_1/FVC z-score for each decile of the PRS for airflow limitation in different age groups from one pediatric and one adult cohort. Scatter plot showing the link between the genetic risk for COPD and lung function. Subjects were classified into ten equally sized deciles based on the distribution of scaled estimates of the PRS for airflow limitation (*x*-axis). The mean FEV_1/FVC z-score is shown for each decile group (*y*-axis) in BAMSE-W2 (panel A) and HUNT (panel B) as a representation of the whole set of participating cohorts. Color-based dots represent the age group with available spirometry data in each cohort. FEV_1: forced expiratory volume in one second; FVC: forced vital capacity; PRS: polygenic risk score; W2: Genotyping Wave 2.

Figure S5. Evaluation of the difference in FEV_1/FVC in different deciles of the PRS distribution across age groups. Plot showing the results of linear regression models evaluating the statistical difference in FEV_1/FVC z-score in decile 2 onwards compared to the bottom decile. These analyses were performed in BAMSE-W2 and HUNT as a representation of all participating cohorts. The decile group of the PRS for airflow limitation is shown in the *x*-axis. The effect estimate of the difference in FEV_1/FVC z-score between each decile and decile 1 is represented in the *y*-axis by boxes color-coded based on the age group and the significance level accounting for the false discovery rate. The reference group (decile 1) is indicated by a black box, whereas the gray boxes represent the deciles with a non-significant difference in FEV_1/FVC from the bottom decile (*q*-value>0·05). The results are shown for each age group in panel A-C for BAMSE-W2, and panel D-G for HUNT. FEV_1: forced expiratory volume in one second; FVC: forced vital capacity; PRS: polygenic risk score; W2: Genotyping Wave 2.

Figure S6. Forest plot of association effects of the PRS for airflow limitation with FEV_1 across age groups up to 50 years. The effects are shown in terms of β estimates for each cohort and after performing an age-stratified meta-analysis (random-effects model) with blue boxes and a red diamond, respectively. The 95% Confidence Intervals (95% CI) are represented by blue dash lines. No meta-analysis was performed in the adulthood, 41-50 years group, and the results provided correspond only to the HUNT cohort. Results are shown for the basic association model, adjusted by Principal Components of genetic ancestry and any cohort-specific covariates. FEV_1: forced expiratory volume in one second; PRS: polygenic risk score; W1: Genotyping Wave 1; W2: Genotyping Wave 2.

Figure S7. Forest plot of the effect size of the association of the PRS for airflow limitation with FVC from preschool age to adulthood (41-50 years). The association results in each cohort and time point as well as the age-stratified meta-analysis of each age group are shown (random-effects model). The blue boxes and dash lines represent the effect size and 95% Confidence Interval (95% CI) obtained in each cohort, whereas the red diamonds show the estimate of the meta-analysis of association results per age group. The association effect size shown for adults (41-50 years of age) corresponds to the HUNT cohort. Results are shown for the basic association model, adjusted by Principal Components of genetic ancestry and any cohort-specific covariates. FVC: forced vital capacity; PRS: polygenic risk score; W1: Genotyping Wave 1; W2: Genotyping Wave 2.

Figure S8. Forest plot comparing the effect size of the association with lung function obtained from the age-stratified meta-analysis of those age groups with available data for two time points from the same cohorts. Blue boxes represent the association effects (β estimate) from the main composition of age groups selected for the meta-analysis. The time point with the largest sample size was selected per group. The effect size obtained from the meta-analysis with the alternative classification replacing the discarded time points is indicated by a red box. The corresponding 95% Confidence Intervals (95% CI) are represented by dash lines. Results are shown for the basic association model, adjusted by Principal Components of genetic ancestry and any cohort-specific covariates.

Figure S9. Forest plot of the age-stratified meta-analysis of the association between the PRS for airflow limitation and z-scores of spirometry measurements in adults up to 50 years of age comparing the basic association model and sensitivity analyses accounting for smoking. The boxes represent the association effect obtained in adults aged up to 50 years after the age-stratified meta-analysis (random-effects model) from the basic association model (blue), sensitivity analysis accounting for smoking status (green), and tobacco pack-years in active smokers (orange). Exceptionally, the effect size obtained from each regression model shown for the oldest adulthood group (41-50 years of age) corresponds to the result obtained in the HUNT cohort. The basic association model was adjusted by Principal Components of genetic ancestry and any cohort-specific covariates. The sensitivity analyses accounting for active smoking also included a covariate related to the smoking status. The analyses exploring the effect of tobacco pack-years were restricted to only participants with reported active smoking habits and were additionally adjusted by a variable obtained by the multiplication of the number of smoking years by the number of daily cigarettes and divided by 20 cigarettes often contained in a package. Dash lines of each corresponding color represent the 95% Confidence Intervals (95% CI) of the size of the association effect. FEV_1: forced expiratory volume in one second; FVC: forced vital capacity; PRS: polygenic risk score.

Figure S10. Forest plot of results from the age-stratified meta-analysis of the association of the PRS for airflow limitation with lung function comparing the basic association model and sensitivity analyses stratified by sex. The color-based boxes show the effect size of the association with each lung function measure from preschool age to adulthood (41-50 years), as a result of applying the basic association model in the whole set of individuals (blue), males (yellow), and females (purple). Principal Components of genetic ancestry and any cohort-specific were included as covariates in the association analyses. The 95% Confidence Interval (95% CI) of the effect magnitude is represented by dash lines of the same color. The results obtained in the HUNT cohort represent the last adulthood group (41-50 years of age). In both the basic association model and sex-stratified sensitivity analyses, Principal Components of genetic ancestry and any cohort-specific were included as covariates. FEV_1: forced expiratory volume in one second; FVC: forced vital capacity; PRS: polygenic risk score.

Figure S11. Forest plot of the age-stratified meta-analysis of association results with spirometry measurements in adults up to 50 years of age from the basic association model and sensitivity analyses accounting for smoking in males (panel A) and females (panel B). The association effect of the meta-analysis (random-effects model) of individual cohort results is represented by boxes colored based on the regression model: basic (blue), sensitivity analysis adjusting by smoking status (green), and tobacco pack-years only in smokers (orange). Dash lines show the 95% Confidence Interval (95% CI) of the association effect. The basic association model was adjusted by Principal Components of genetic ancestry and any cohort-specific covariates. The sensitivity analyses accounting for active smoking also included a covariate related to the smoking status. The analyses exploring the effect of tobacco pack-years were restricted to only participants with reported active smoking habits and were additionally adjusted by a variable obtained by the multiplication of the number of smoking years by the number of daily cigarettes and divided by 20 cigarettes often contained in a package. The results shown for adulthood, 41-50 years were obtained only in the HUNT cohort. FEV_1: forced expiratory volume in one second; FVC: forced vital capacity.

Figure S12. Forest plot of the age-stratified meta-analysis of results from the evaluation of the association between the PRS for airflow limitation and lung function from sensitivity analyses stratified by sex accounting for smoking status (panel A) and tobacco pack-years (panel B). The effect size of the association with each lung function measure from preschool age to adulthood (41-50 years) in the whole set of individuals (blue), males (yellow), or females (purple) is represented by colored boxes together with their corresponding 95% Confidence Interval (95% CI) through dash lines. The sensitivity analyses accounting for active smoking also included a covariate related to the smoking status apart from Principal Components of genetic ancestry and any cohort-specific variables. The analyses exploring the effect of tobacco pack-years were restricted to only participants with reported active smoking habits and were additionally adjusted by a variable obtained by the multiplication of the number of smoking years by the number of daily cigarettes and divided by 20 cigarettes often contained in a package. The results shown for the adulthood, 41-50 years age group were obtained only in the HUNT cohort. Results are shown for adults up to 50 years of age. FEV_1: forced expiratory volume in one second; FVC: forced vital capacity; PRS: polygenic risk score.

SUPPLEMENTARY TABLES

Table S1. Main characteristics of the participating CADSET cohorts.

	**ALSPAC**	**Ashford**	**BAMSE**	**COPSAC$_{2000}$**
Sample size [a]	8,943	348	2,636	358
Recruitment country	United Kingdom	United Kingdom	Sweden	Denmark
Type of cohort	Longitudinal, population-based	Longitudinal, population-based	Longitudinal, population-based	Longitudinal, asthma-focused
Age group	Children - young adults	Adolescents	Children - young adults	Children - young adults
Available time points [b]	8, 15, 24 years	15 years	8, 16, 24 years	7, 12, 18 years
Genotyping platform	Illumina HumanHap550 BeadChip (Illumina)	Illumina Human610-Quad BeadChip (Illumina)	Illumina Human610-Quad BeadChip, Illumina Infinium Global Screening Array-24 v1.0 BeadChip (Illumina)	Illumina Infinium OmniExpressExome-8 v1.6 BeadChip (Illumina)
Imputation of genetic variants				
Software for imputation	Minimac4	PBWT	PBWT	Minimac4
Server	MIS	SIS	SIS	MIS
Software for haplotype phasing	SHAPEIT2	EAGLE2	EAGLE2	EAGLE2
Reference panel	HRC r1.1	HRC r1.1	HRC r1.1	HRC r1.1

[a] Number of individuals with available genome-wide genotype data included in the calculation of the PRS for airflow limitation; [b] Average age of the individuals at the data collection follow-ups included in this study; [c] Subjects were classified into groups of similar age for this study; [d] Subjects aged >50 years old from HUNT and The Rotterdam Study were included in an additional validation of the association with spirometry measurements; [e] Participants have been grouped based on the time point when the pulmonary function test was conducted.
1KGP: 1,000 Genomes Project; CADSET: Chronic Airway Diseases Early Stratification; GoNL: The Genome of the Netherlands Project; HRC: The Haplotype Reference Consortium; HUNT-WGS: HUNT-specific reference panel constructed based on whole-genome sequencing of 2,201 participants; MIS: Michigan Imputation Server; NA: not available; PBWT: Positional Burrows-Wheeler Transform; SIS: Sanger Imputation Server.

Table S1 (continuation). Main characteristics of the participating CADSET cohorts.

	$COPSAC_{2010}$	Generation R	GINIplus/LISA North	GINIplus/LISA South
Sample size [a]	618	5,756	792	1,511
Recruitment country	Denmark	The Netherlands	Germany	Germany
Type of cohort	Longitudinal, population-based	Longitudinal, population-based	Longitudinal, population-based	Longitudinal, population-based
Age group	Children	Children - adolescents	Children - adolescents	Children - adolescents
Available time points [b]	10 years	9, 13 years	10, 15 years	6, 15 years
Genotyping platform	Illumina Infinium OmniExpressExome-8 v1.6 BeadChip (Illumina)	Illumina Human610-Quad BeadChip, Illumina Infinium Global Screening Array-24 v2.0 BeadChip (Illumina)	Illumina Infinium Global Screening Array-24 v2.0 BeadChip (Illumina)	Affymetrix Genome-Wide Human SNP Array 5.0, Affymetrix Genome-Wide Human SNP Array 6.0 (Thermo Fisher)
Imputation of genetic variants				
Software for imputation	Minimac4	Minimac4	Minimac4	Minimac4
Server	MIS	NA	MIS	MIS
Software for haplotype phasing	EAGLE2	MACH; SHAPEIT2	NA	NA
Reference panel	HRC r1.1	HapMap Project Phase II Release 22, 1KGP Phase III	HRC r1.1	HRC r1.1

[a] Number of individuals with available genome-wide genotype data included in the estimation of the PRS for airflow limitation; [b] Average age of the individuals at the data collection follow-ups included in this study; [c] Subjects were classified into groups of similar age for this study; [d] Subjects aged >50 years old from HUNT and The Rotterdam Study were included in an additional validation of the association with spirometry measurements; [e] Participants have been grouped based on the time point when the pulmonary function test was conducted.
1KGP: 1,000 Genomes Project; CADSET: Chronic Airway Diseases Early Stratification; GoNL: The Genome of the Netherlands Project; HRC: The Haplotype Reference Consortium; HUNT-WGS: HUNT-specific reference panel constructed based on whole-genome sequencing of 2,201 participants; MIS: Michigan Imputation Server; NA: not available; PBWT: Positional Burrows-Wheeler Transform; SIS: Sanger Imputation Server.

Table S1 (continuation). Main characteristics of the participating CADSET cohorts.

	HUNT	INMA	IoWBC	Lifelines
Sample size [a]	69,717	2,034	956	3,864
Recruitment country	Norway	Spain	United Kingdom	The Netherlands
Type of cohort	Longitudinal, population-based	Longitudinal, population-based	Longitudinal, population-based	Cross-sectional, population-based
Age group	Young adults - adults	Children - young adults	Children - young adults	Young adults - adults
Available time points [b]	20-30, 31-40, 41-50, >50 years [c,d]	4, 7, 10, 11, 14, 18 years	10, 18, 26 years	18-30, 31-40 years [c]
Genotyping platform	Illumina Infinium HumanCoreExome-24+ BeadChip (Illumina)	Illumina HumanOmni1-Quad BeadChip,<br>Illumina Infinium Global Screening Array BeadChip (multiple versions) (Illumina)	Illumina Human Infinium Omni2.5-8 v1.3 BeadChip (Illumina)	Illumina Infinium HumanCytoSNP-12 v2.0 BeadChip (Illumina)
Imputation of genetic variants				
Software for imputation	Minimac3	Minimac4	PBWT	IMPUTE2
Server	MIS	MIS	SIS	NA
Software for haplotype phasing	EAGLE2	NA	EAGLE2	SHAPEIT2
Reference panel	HRC r1.1, HUNT-WGS v1.1	HRC r1.1	HRC r1.1	1KGP Phase III, GoNL

[a] Number of individuals with available genome-wide genotype data included in the estimation of the PRS for airflow limitation; [b] Average age of the individuals at the data collection follow-ups included in this study; [c] Subjects were classified into groups of similar age for this study; [d] Subjects aged >50 years old from HUNT and The Rotterdam Study were included in an additional validation of the association with spirometry measurements; [e] Participants have been grouped based on the time point when the pulmonary function test was conducted.

1KGP: 1,000 Genomes Project; CADSET: Chronic Airway Diseases Early Stratification; GoNL: The Genome of the Netherlands Project; HRC: The Haplotype Reference Consortium; HUNT-WGS: HUNT-specific reference panel constructed based on whole-genome sequencing of 2,201 participants; MIS: Michigan Imputation Server; NA: not available; PBWT: Positional Burrows-Wheeler Transform; SIS: Sanger Imputation Server.

Table S1 (continuation). Main characteristics of the participating CADSET cohorts.

	MAAS	PIAMA	Rotterdam Study	SEATON
Sample size [a]	852	1,526	11,496	552
Recruitment country	United Kingdom	The Netherlands	The Netherlands	United Kingdom
Type of cohort	Longitudinal, population-based	Longitudinal, population-based	Longitudinal, population-based	Longitudinal, population-based
Age group	Children - young adults	Children - adolescents	Adults	Children - adolescents
Available time points [b]	8, 16, 19 years	8, 12, 16 years	3 different time points (50-100 years) [d,e]	10, 15 years
Genotyping platform	Illumina Human610-Quad BeadChip (Illumina)	Illumina Human610-Quad BeadChip, Illumina Human OmniExpress BeadChip, Infinium OmniExpressExome BeadChip, Infinium Global Screening Array (Illumina)	Illumina HumanHap550 BeadChip, Illumina Human610-Quad BeadChip (Illumina)	Illumina Human610-Quad BeadChip (Illumina)
Imputation of genetic variants				
Software for imputation	PBWT	Minimac4	Minimac4	PBWT
Server	SIS	MIS	MIS	SIS
Software for haplotype phasing	EAGLE2	NA	NA	EAGLE2
Reference panel	HRC r1.1	HRC r1.1	HRC r1.1	HRC r1.1

[a] Number of individuals with available genome-wide genotype data included in the estimation of the PRS for airflow limitation; [b] Average age of the individuals at the data collection follow-ups included in this study; [c] Subjects were classified into groups of similar age for this study; [d] Subjects aged >50 years old from HUNT and The Rotterdam Study were included in an additional validation of the association with spirometry measurements; [e] Participants have been grouped based on the time point when the pulmonary function test was conducted. 1KGP: 1,000 Genomes Project; CADSET: Chronic Airway Diseases Early Stratification; GoNL: The Genome of the Netherlands Project; HRC: The Haplotype Reference Consortium; HUNT-WGS: HUNT-specific reference panel constructed based on whole-genome sequencing of 2,201 participants; MIS: Michigan Imputation Server; NA: not available; PBWT: Positional Burrows-Wheeler Transform; SIS: Sanger Imputation Server.

Table S2. Summary statistics of the genome-wide significant SNPs associated with COPD susceptibility in the base dataset.

SNP	Chr. [a]	Chr. Band [b]	Position [c]	Nearest gene(s)	EA/NEA	Freq. [d]	OR (95% CI) [e]	*p*-value
rs9435731	1	p36.13	17306029	*MFAP2*	A/C	0·51	1·06 (1·04-1·08)	$6·6x10^{-10}$
rs76841360	1	p34.3	40060025	*PABPC4*	A/G	0·23	1·08 (1·05-1·10)	$5·0x10^{-10}$
rs4660861	1	p34.1	45946636	*TESK2*	G/T	0·57	1·06 (1·04-1·08)	$4·4x10^{-8}$
rs72673419	1	p32.1	60913143	*C1orf87*	T/C	0·05	1·14 (1·09-1·19)	$4·0x10^{-9}$
rs629619	1	p13.3	111738108	*DENND2D*	T/C	0·20	1·08 (1·06-1·11)	$2·9x10^{-10}$
rs3009947	1	q41	218689155	*TGFB2*	C/T	0·50	1·06 (1·04-1·08)	$4·0x10^{-9}$
rs11118406	1	q41	219924894	*SLC30A10*	T/A	0·28	1·08 (1·05-1·10)	$4·1x10^{-11}$
rs11579382	1	q43	239901006	*CHRM3*	C/G	0·42	1·06 (1·04-1·08)	$6·5x10^{-9}$
rs955277	2	p25.1	9290357	*ASAP2*	T/C	0·61	1·07 (1·05-1·09)	$1·9x10^{-10}$
rs10929386	2	p24.3	15906179	*DDX1*	C/T	0·49	1·06 (1·04-1·08)	$9·1x10^{-9}$
rs12466981	2	p21	42433247	*EML4*	C/T	0·73	1·06 (1·04-1·09)	$4·9x10^{-8}$
rs72902175	2	q24.1	157013035	*NR4A2*	T/C	0·13	1·09 (1·06-1·12)	$1·6x10^{-8}$
rs2571445	2	q35	218683154	*TNS1*	A/G	0·39	1·07 (1·05-1·09)	$3·4x10^{-12}$
rs16825267	2	q36.3	229569919	*PID1*	C/G	0·92	1·19 (1·15-1·24)	$1·8x10^{-20}$
rs62191105	2	q37.3	239872704	*TWIST2*	C/T	0·80	1·09 (1·07-1·12)	$2·6x10^{-12}$
rs2442776	3	p25.3	11640601	*VGLL4*	G/A	0·15	1·09 (1·06-1·12)	$2·0x10^{-10}$
rs1529672	3	p24.2	25520582	*RARB*	C/A	0·83	1·09 (1·06-1·12)	$2·5x10^{-11}$
rs13073544	3	p24.1	29472412	*RBMS3*	C/G	0·28	1·06 (1·04-1·09)	$2·0x10^{-8}$
rs17759204	3	p14.3	55158224	*CACNA2D3*	G/A	0·27	1·07 (1·05-1·10)	$8·8x10^{-11}$
rs62259026	3	p14.3	57746515	*SLMAP*	C/T	0·75	1·07 (1·04-1·09)	$2·4x10^{-8}$
rs4093840	3	q21.1	123077042	*ADCY5*	A/T	0·47	1·06 (1·04-1·08)	$3·9x10^{-10}$
rs2955083	3	q21.3	127961178	*EEFSEC*	A/T	0·88	1·13 (1·10-1·17)	$3·5x10^{-15}$
rs7650602	3	q23	141147414	*ZBTB38*	C/T	0·45	1·06 (1·04-1·08)	$4·9x10^{-8}$

[a] Chromosome; [b] Chromosomal band; [c] Positions based on the GRCh37/hg19 build of the human genome; [d] Frequency of the effect allele in the subjects included in the genome-wide association study of COPD susceptibility selected as the base dataset for the present study; [e] Effect size of the association in terms of odds ratio and 95% confidence interval for the effect alleles.

CI: Confidence Interval; COPD: chronic obstructive pulmonary disease; EA: effect allele; NEA: non-effect allele; SNP: single-nucleotide polymorphism.

Summary statistics are shown for independent SNPs associated with COPD susceptibility (*p*-value$\leq 5x10^{-8}$) reported in the original publication of the base dataset (Sakornsakolpat *et al*. *Nat Genet* 2019;51(3):494-505).

Table S2 (continuation). Summary statistics of the genome-wide significant SNPs associated with COPD susceptibility in the base dataset.

SNP	Chr. [a]	Chr. Band [b]	Position [c]	Nearest gene(s)	EA/NEA	Freq. [d]	OR (95% CI) [e]	*p*-value
rs7642001	3	q26.2	168746145	*MECOM*	A/G	0·37	1·08 (1·06-1·10)	$1{\cdot}1x10^{-14}$
rs4585380	4	q13.3	75673363	*BTC*	G/A	0·74	1·07 (1·05-1·09)	$3{\cdot}4x10^{-9}$
rs7671261	4	q22.1	89883818	*FAM13A*	A/G	0·55	1·09 (1·07-1·11)	$1{\cdot}4x10^{-18}$
rs34712979	4	q24	106819053	*NPNT*	A/G	0·25	1·18 (1·16-1·21)	$3{\cdot}0x10^{-46}$
rs13140176	4	q31.21	145489098	*HHIP*	A/G	0·61	1·18 (1·16-1·20)	$4{\cdot}1x10^{-59}$
rs1551943	5	q11.2	52195033	*ITGA1*	A/G	0·23	1·08 (1·05-1·10)	$5{\cdot}8x10^{-10}$
rs34651	5	q13.2	72144005	*TNPO1*	C/T	0·08	1·11 (1·07-1·15)	$3{\cdot}0x10^{-8}$
rs153916	5	q15	95036700	*SPATA9*	T/C	0·55	1·06 (1·04-1·08)	$6{\cdot}3x10^{-10}$
rs62375246	5	q31.1	132439010	*HSPA4*	A/T	0·26	1·06 (1·04-1·09)	$2{\cdot}2x10^{-8}$
rs10037493	5	q32	147854970	*HTR4*	C/T	0·55	1·13 (1·10-1·15)	$2{\cdot}6x10^{-33}$
rs979453	5	q33.1	150595073	*CCDC69*	G/A	0·34	1·06 (1·04-1·08)	$1{\cdot}4x10^{-8}$
rs10866659	5	q33.3	156937043	*ADAM19*	G/A	0·35	1·09 (1·07-1·11)	$1{\cdot}2x10^{-16}$
rs12519165	5	q35.1	170901586	*FGF18*	A/T	0·38	1·07 (1·05-1·09)	$1{\cdot}1x10^{-9}$
rs1334576	6	p24.3	7211818	*RREB1*	A/G	0·42	1·06 (1·04-1·08)	$1{\cdot}2x10^{-8}$
rs9350191	6	p22.3	19842661	*ID4*	T/C	0·85	1·12 (1·09-1·15)	$5{\cdot}1x10^{-14}$
rs13198656	6	p22.3	22004909	*PRL*	T/C	0·56	1·06 (1·04-1·08)	$1{\cdot}2x10^{-9}$
rs2284174	6	p21.33	30713580	*IER3*	C/T	0·22	1·12 (1·10-1·15)	$2{\cdot}1x10^{-21}$
rs2070600	6	p21.32	32151443	*AGER*	C/T	0·94	1·21 (1·15-1·26)	$1{\cdot}1x10^{-17}$
rs2806356	6	q21	109266255	*ARMC2*	C/T	0·18	1·10 (1·08-1·13)	$2{\cdot}9x10^{-15}$
rs674621	6	q22.1	117257018	*RFX6*	C/T	0·32	1·06 (1·04-1·08)	$7{\cdot}6x10^{-9}$
rs646695	6	q24.1	140280398	*CITED2*	C/T	0·24	1·08 (1·05-1·10)	$4{\cdot}6x10^{-11}$
rs9399401	6	q24.1	142668901	*ADGRG6*	T/C	0·72	1·16 (1·13-1·18)	$1{\cdot}6x10^{-40}$
rs798565	7	p22.3	2752152	*AMZ1*	G/A	0·71	1·07 (1·04-1·09)	$3{\cdot}9x10^{-9}$

[a] Chromosome; [b] Chromosomal band; [c] Positions based on the GRCh37/hg19 build of the human genome; [d] Frequency of the effect allele in the subjects included in the genome-wide association study of COPD susceptibility selected as the base dataset for the present study; [e] Effect size of the association in terms of odds ratio and 95% confidence interval for the effect alleles.

CI: Confidence Interval; COPD: chronic obstructive pulmonary disease; EA: effect allele; NEA: non-effect allele; SNP: single-nucleotide polymorphism.

Summary statistics are shown for independent SNPs associated with COPD susceptibility (p-value$\leq 5x10^{-8}$) reported in the original publication of the base dataset (Sakornsakolpat *et al*. *Nat Genet* 2019;51(3):494-505).

Table S2 (continuation). Summary statistics of the genome-wide significant SNPs associated with COPD susceptibility in the base dataset.

**SNP**	**Chr.** [a]	**Chr. Band** [b]	**Position** [c]	**Nearest gene(s)**	**EA/NEA**	**Freq.** [d]	**OR (95% CI)** [e]	***p*-value**
rs2040732	7	p21.1	20418134	*ITGB8*	C/T	0·58	1·06 (1·04-1·08)	$6{\cdot}9x10^{-9}$
rs2897075	7	q22.1	99630342	*ZKSCAN1*	C/T	0·63	1·07 (1·04-1·09)	$7{\cdot}3x10^{-10}$
rs9329170	8	p23.1	8697658	*MFHAS1*	C/G	0·86	1·10 (1·07-1·13)	$3{\cdot}6x10^{-10}$
rs10114763	9	p24.2	4143749	*GLIS3*	T/A	0·42	1·07 (1·05-1·09)	$8{\cdot}7x10^{-13}$
rs156394	9	p21.3	23588684	*ELAVL2*	T/C	0·53	1·07 (1·05-1·09)	$3{\cdot}8x10^{-12}$
rs7866939	9	q21.32	85126163	*RASEF*	C/T	0·33	1·06 (1·04-1·08)	$1{\cdot}7x10^{-8}$
rs10760580	9	q22.33	101661650	*COL15A1*	G/A	0·71	1·07 (1·05-1·10)	$1{\cdot}2x10^{-10}$
rs803923	9	q33.1	119401650	*ASTN2*	A/G	0·53	1·06 (1·04-1·08)	$2{\cdot}7x10^{-8}$
rs7068966	10	p13	12277992	*CDC123*	C/T	0·49	1·10 (1·08-1·12)	$6{\cdot}2x10^{-23}$
rs2579762	10	q22.3	78318879	*LRMDA*	C/A	0·47	1·06 (1·04-1·09)	$2{\cdot}6x10^{-10}$
rs721917	10	q22.3	81706324	*SFTPD*	G/A	0·42	1·06 (1·04-1·08)	$2{\cdot}2x10^{-8}$
rs1570221	10	q24.33	105656874	*STN1*	A/G	0·35	1·06 (1·04-1·08)	$2{\cdot}2x10^{-8}$
rs4757118	11	p15.2	13171236	*ARNTL*	T/C	0·54	1·06 (1·04-1·08)	$3{\cdot}8x10^{-9}$
rs117261012	11	q14.2	86444761	*PRSS23*	G/A	0·16	1·09 (1·06-1·12)	$6{\cdot}9x10^{-10}$
rs11049386	12	p11.22	28320536	*CCDC91*	T/A	0·71	1·06 (1·04-1·09)	$2{\cdot}7x10^{-8}$
rs7307510	12	q23.1	96237570	*SNRPF*	C/T	0·81	1·08 (1·05-1·10)	$2{\cdot}6x10^{-9}$
rs7958945	12	q24.21	115947901	*MED13L*	G/A	0·36	1·06 (1·04-1·09)	$1{\cdot}0x10^{-9}$
rs9525927	13	q14.11	44842503	*SERP2*	G/A	0·19	1·08 (1·05-1·10)	$2{\cdot}8x10^{-9}$
rs72699855	14	q32.12	93105953	*RIN3*	G/C	0·81	1·08 (1·05-1·11)	$4{\cdot}8x10^{-9}$
rs72731149	15	q21.2	49984710	*DTWD1*	G/C	0·91	1·12 (1·08-1·16)	$8{\cdot}3x10^{-9}$
rs1441358	15	q23	71612514	*THSD4*	G/T	0·34	1·13 (1·11-1·15)	$7{\cdot}4x10^{-33}$
rs55676755	15	q25.1	78898932	*CHRNA3*	G/C	0·34	1·11 (1·09-1·14)	$2{\cdot}7x10^{-26}$
rs10152300	15	q25.2	84392907	*ADAMTSL3*	G/A	0·23	1·08 (1·06-1·11)	$4{\cdot}2x10^{-12}$

[a] Chromosome; [b] Chromosomal band; [c] Positions based on the GRCh37/hg19 build of the human genome; [d] Frequency of the effect allele in the subjects included in the genome-wide association study of COPD susceptibility selected as the base dataset for the present study; [e] Effect size of the association in terms of odds ratio and 95% confidence interval for the effect alleles.

CI: Confidence Interval; COPD: chronic obstructive pulmonary disease; EA: effect allele; NEA: non-effect allele; SNP: single-nucleotide polymorphism.

Summary statistics are shown for independent SNPs associated with COPD susceptibility (p-value≤$5x10^{-8}$) reported in the original publication of the base dataset (Sakornsakolpat *et al*. *Nat Genet* 2019;51(3):494-505).

Table S2 (continuation). Summary statistics of the genome-wide significant SNPs associated with COPD susceptibility in the base dataset.

SNP	Chr. [a]	Chr. Band [b]	Position [c]	Nearest gene(s)	EA/NEA	Freq. [d]	OR (95% CI) [e]	*p*-value
rs56134392	16	p13.13	10709013	*TEKT5*	C/T	0·35	1·06 (1·04-1·08)	$4·5x10^{-8}$
rs8044657	16	q21	58022625	*TEPP*	G/A	0·90	1·11 (1·07-1·15)	$1·1x10^{-8}$
rs4888379	16	q23.1	75340231	*CFDP1*	T/A	0·58	1·10 (1·08-1·12)	$5·9x10^{-21}$
rs8080772	17	q11.2	28413129	*EFCAB5*	T/C	0·64	1·06 (1·04-1·08)	$1·4x10^{-8}$
rs34727469	17	q12	36835079	*RPL23*	T/C	0·14	1·09 (1·06-1·12)	$1·1x10^{-8}$
rs62065216	17	q21.1	38218773	*THRA*	A/G	0·42	1·06 (1·04-1·08)	$8·1x10^{-9}$
rs12373142	17	q21.31	43924200	*SPPL2C*	G/C	0·22	1·08 (1·05-1·10)	$9·9x10^{-10}$
rs11655567	17	q24.3	69216687	*SOX9*	C/T	0·49	1·06 (1·04-1·08)	$1·9x10^{-9}$
rs647097	18	p11.22	8808464	*MTCL1*	C/T	0·27	1·08 (1·05-1·10)	$1·0x10^{-11}$
rs72626215	19	q13.32	46294136	*DMWD*	G/A	0·73	1·07 (1·05-1·10)	$1·7x10^{-9}$
rs2096468	21	q22.11	35661745	*KCNE2*	A/C	0·45	1·06 (1·04-1·08)	$4·0x10^{-8}$
rs9617650	22	q11.21	18488883	*MICAL3*	G/C	0·79	1·08 (1·06-1·11)	$4·4x10^{-10}$
rs73158393	22	q12.3	33335386	*SYN3*	C/G	0·74	1·07 (1·05-1·09)	$7·7x10^{-9}$

[a] Chromosome; [b] Chromosomal band; [c] Positions based on the GRCh37/hg19 build of the human genome; [d] Frequency of the effect allele in the subjects included in the genome-wide association study of COPD susceptibility selected as the base dataset for the present study; [e] Effect size of the association in terms of odds ratio and 95% confidence interval for the effect alleles.

CI: Confidence Interval; COPD: chronic obstructive pulmonary disease; EA: effect allele; NEA: non-effect allele; SNP: single-nucleotide polymorphism.

Summary statistics are shown for independent SNPs associated with COPD susceptibility (p-value$\leq 5x10^{-8}$) reported in the original publication of the base dataset (Sakornsakolpat *et al. Nat Genet* 2019;51(3):494-505).

Table S3. Definition of age groups for the meta-analysis of associations between the PRS for airflow limitation and spirometry measurements in participants aged up to 50 years.

Age group	Age range	Cohort	Time point	Sample size [a]
Preschool age	0-6 years	INMA	4 years	559
		GINIplus/LISA South	6 years	106
School age	7-10 years	$COPSAC_{2000}$	7 years	292
		INMA [b]	7 years	925
		ALSPAC	8 years	4,871
		BAMSE-W1 [c]	8 years	335
		BAMSE-W2 [c]	8 years	1,230
		MAAS	8 years	640
		PIAMA	8 years	907
		Generation R	9 years	2,147
		$COPSAC_{2010}$	10 years	530
		GINIplus/LISA North	10 years	374
		IoWBC	10 years	754
		SEATON	10 years	382
Puberty	11-15 years	INMA [d]	11 years	792
		$COPSAC_{2000}$	12 years	293
		PIAMA	12 years	1,018
		Generation R	13 years	1,897
		ALSPAC	15 years	3,332
		ASHFORD	15 years	322
		GINIplus/LISA North	15 years	496
		GINIplus/LISA South	15 years	843
		SEATON	15 years	330
Post-puberty	16-17 years	BAMSE-W1 [c]	16 years	305
		BAMSE-W2 [c]	16 years	1,185
		MAAS	16 years	502
		PIAMA	16 years	653
Adulthood	18-30 years	$COPSAC_{2000}$	18 years	317
		INMA	18 years	87
		IoWBC [e]	18 years	669
		Lifelines	18-30 years	859
		MAAS	19 years	436
		HUNT	20-30 years	2,848
		ALSPAC	24 years	2,590
		BAMSE-W1 [c]	24 years	282
		BAMSE-W2 [c]	24 years	1,044
Adulthood	31-40 years	HUNT	31-40 years	3,107
		Lifelines	31-40 years	3,005
Adulthood	41-50 years	HUNT	41-50 years	4,142

[a] Number of individuals with available genotype data and spirometry measurements included in the association analyses; [b] INMA, 10 years (n=65) was excluded from the meta-analysis to avoid duplicated samples within the school age group, and INMA, 7 years (n=925) was kept; [c] The PRS for airflow limitation was separately estimated in each of the genotyping Waves of the BAMSE cohort (Wave 1, n=463; Wave 2, n=2,173); [d] INMA, 14 years (n=188) was discarded from the meta-analysis to avoid duplicates within the puberty age group, and INMA, 11 years (n=792) was kept; [e] IoWBC, 26 years (n=432) was discarded from the meta-analysis to avoid duplicates within the adulthood age group (18-30 years), and IoWBC, 18 years (n=669) was kept.
W1: Genotyping Wave 1; W2: Genotyping Wave 2.

Tables S4-S5: Refer to the file named *“eclinm-D-24-00532R1_tableS4_S5_clean.xlsx”*

Table S6. Comparison of PRS estimates obtained with different numbers of genetic variants in the BAMSE cohort.

	Proportion of SNPs included [a]	Nr. SNPs	Minimum	Quartile 1	Mean	Quartile 3	Maximum	R^2 Pearson [b]
PRS - Raw estimates	100%	79	-0·876	0·288	0·591	0·881	1·979	1
	90%	71	-0·985	0·393	0·654	0·929	2·029	0·916
	70%	55	-0·925	-0·057	0·197	0·450	1·430	0·851
	50%	40	-1·130	-0·300	-0·096	0·101	0·894	0·679
	30%	24	-1·318	-0·296	-0·109	0·080	0·979	0·634
PRS - Scaled estimates (z-score) [c]	100%	79	-3·359	-0·694	0	0·663	3·178	1
	90%	71	-4·069	-0·647	0	0·682	3·414	0·916
	70%	55	-3·096	-0·701	0	0·695	3·401	0·851
	50%	40	-3·463	-0·683	0	0·660	3·313	0·679
	30%	24	-4·324	-0·668	0	0·676	3·891	0·634

[a] For each proportion, the set of SNPs was randomly extracted from the whole set of variants; [b] Correlation between the PRS estimates was calculated using the whole set of SNPs and each subset with different proportions of SNPs included; [c] Raw estimates were transformed into z-scores.
PRS: polygenic risk score; SNP: single-nucleotide polymorphism. The PRS for airflow limitation was separately estimated in each of the genotyping waves of the BAMSE cohort: Genotyping Wave 1 (n=463), and Genotyping Wave 2 (n=2,173).
Results are shown for genotyping Wave 2 of the BAMSE cohort.

Table S7. Distribution of the scaled estimates of the PRS for airflow limitation (z-scores) in each of the participating CADSET cohorts.

Cohort	Type of sample	Sample size	Nr. SNPs [a]	Minimum	Quartile 1	Mean	Quartile 3	Maximum
ALSPAC	Whole cohort [b]	8,943	79	-3·799	-0·658	0	0·664	3·430
Ashford	Whole cohort [b]	348	79	-3·196	-0·691	0	0·681	3·403
BAMSE [c]	Whole cohort, W1 [b,d]	463	79	-3·221	-0·645	0	0·648	2·975
	Whole cohort, W2 [b,e]	2,173	79	-3·359	-0·694	0	0·663	3·178
$COPSAC_{2000}$	Whole cohort [b]	358	78	-3·860	-0·666	0	0·657	4·029
$COPSAC_{2010}$	Whole cohort [b]	618	79	-3·726	-0·670	0	0·690	2·894
Generation R	Whole cohort [b]	5,756	77	-3·812	-0·680	0	0·669	3·425
GINIplus/LISA North	Whole cohort [b]	792	79	-3·045	-0·685	0	0·697	3·034
GINIplus/LISA South	Whole cohort [b]	1,511	79	-3·234	-0·706	0	0·674	2·984
HUNT	Whole cohort [b]	69,717	78	-4·259	-0·674	0	0·678	4·309
INMA	Whole cohort [b]	2,034	79	-3·719	-0·671	0	0·660	3·243
IoWBC	Whole cohort [b]	956	79	-3·245	-0·724	0	0·701	2·592
Lifelines	Age-specific (18-30) [f]	859	80	-3·209	-0·707	0	0·662	3·356
	Age-specific (31-40) [f]	3,005	80	-3·286	-0·663	0	0·648	3·488
MAAS	Whole cohort [b]	852	79	-2·888	-0·672	0	0·702	2·685
PIAMA	Whole cohort [b]	1,526	69	-3·944	-0·682	0	0·677	3·533
Rotterdam Study	Whole cohort [b]	11,496	79	-4·068	-0·682	0	0·691	4·256
SEATON	Whole cohort [b]	552	79	-2·764	-0·682	0	0·708	2·640

[a] Number of SNPs selected to be included in the PRS calculation after the quality control procedures; [b] Subjects from the whole cohort with available genome-wide imputation data were included in the PRS calculation regardless of the age or availability of any clinical information; [c] The PRS for airflow limitation was separately estimated in each of the genotyping Waves of the BAMSE cohort; [d] Genotyping Wave 1 (n=463); [e] Genotyping Wave 2 (n=2,173); [f] The PRS estimation was separately conducted in two groups of participants based on age similarity.
PRS: polygenic risk score; SNP: single-nucleotide polymorphism; W1: Genotyping Wave 1; W2: Genotyping Wave 2.

Table S8. Results of the association of the PRS for airflow limitation with spirometry measurements up to 50 years of age in each cohort.

Cohort	n_{PRS} [a]	Time point [b]	$n_{Association}$ [c]	FEV$_1$ [f] β (95%CI) [d]	FEV$_1$ [f] *p*-value	FEV$_1$ [f] *q*-value [e]	FVC [f] β (95%CI) [d]	FVC [f] *p*-value	FVC [f] *q*-value [e]	FEV$_1$/FVC [f] β (95%CI) [d]	FEV$_1$/FVC [f] *p*-value	FEV$_1$/FVC [f] *q*-value [e]
ALSPAC	8,943	8 years	4,871	-0·07 (-0·10, -0·04)	3·83 x 10^{-6}	**1·05 x 10^{-5}**	0·01 (-0·02, 0·03)	0·702	1	-0·12 (-0·15, -0·09)	5·59 x 10^{-15}	**3·07 x 10^{-14}**
		15 years	3,332	-0·05 (-0·10, -0·01)	0·016	**0·044**	0·02 (-0·02, 0·06)	0·307	0·563	-0·14 (-0·18, -0·10)	3·61 x 10^{-12}	**1·99 x 10^{-11}**
		24 years	2,590	-0·10 (-0·13, -0·06)	6·11 x 10^{-7}	**1·68 x 10^{-5}**	0·00 (-0·04, 0·04)	0·988	1	-0·14 (-0·17, -0·10)	6·53 x 10^{-14}	**3·59 x 10^{-13}**
Ashford	348	15 years	322	0·04 (-0·08, 0·15)	0·531	1	0·04 (-0·08, 0·16)	0·475	1	-0·02 (-0·15, 0·10)	0·687	1
BAMSE [g]	463 [h]	8 years	335	-0·09 (-0·18, 0·01)	0·073	0·201	0·02 (-0·07, 0·11)	0·659	1	-0·15 (-0·24, -0·06)	1·81 x 10^{-3}	**9·96 x 10^{-3}**
		16 years	305	-0·07 (-0·17, 0·03)	0·162	0·446	0·02 (-0·08, 0·12)	0·670	1	-0·12 (-0·23, -0·01)	0·040	0·218
		24 years	282	-0·09 (-0·19, 0·00)	0·052	0·144	0·01 (-0·08, 0·11)	0·782	1	-0·16 (-0·26, -0·06)	2·56 x 10^{-3}	**0·014**
	2,173 [i]	8 years	1,230	-0·12 (-0·17, -0·06)	1·55 x 10^{-5}	**4·26 x 10^{-5}**	-0·03 (-0·08, 0·02)	0·214	0·392	-0·13 (-0·18, -0·08)	5·64 x 10^{-7}	**3·10 x 10^{-6}**
		16 years	1,185	-0·14 (-0·20, -0·08)	8·07 x 10^{-7}	**2·22 x 10^{-6}**	-0·02 (-0·08, 0·03)	0·459	0·842	-0·17 (-0·23, -0·12)	7·80 x 10^{-10}	**4·29 x 10^{-9}**
		24 years	1,044	-0·12 (-0·17, -0·07)	9·70 x 10^{-6}	**2·67 x 10^{-5}**	-0·02 (-0·07, 0·04)	0·558	1	-0·15 (-0·20, -0·09)	6·25 x 10^{-8}	**3·44 x 10^{-7}**
COPSAC2000 [j]	358	7 years	292	0·02 (-0·10, 0·13)	0·736	1	0·10 (-0·01, 0·22)	0·083	0·229	-0·18 (-0·29, -0·07)	1·05 x 10^{-3}	**5·78 x 10^{-3}**
		12 years	293	-0·08 (-0·19, 0·04)	0·186	0·497	0·06 (-0·05, 0·18)	0·271	0·497	-0·23 (-0·34, -0·12)	9·03 x 10^{-5}	**4·97 x 10^{-4}**
		18 years	317	-0·06 (-0·17, 0·05)	0·288	0·528	0·08 (-0·02, 0·19)	0·132	0·363	-0·19 (-0·30, -0·09)	4·12 x 10^{-4}	**2·27 x 10^{-3}**
COPSAC2010 [j]	618	10 years	530	-0·03 (-0·05, -0·01)	0·015	0·055	-0·01 (-0·04, 0·02)	0·446	0·818	-0·10 (-0·19, -0·02)	0·020	0·055
Generation R [k]	5,756	9 years	2,147	-0·08 (-0·12, -0·04)	2·91 x 10^{-5}	**8·00 x 10^{-5}**	0·01 (-0·03, 0·05)	0·436	0·799	-0·16 (-0·20, -0·12)	5·08 x 10^{-16}	**2·79 x 10^{-15}**
		13 years	1,897	-0·11 (-0·13, -0·09)	5·41 x 10^{-8}	**1·49 x 10^{-7}**	-0·01 (-0·03, 0·01)	0·470	0·862	-0·17 (-0·19, -0·15)	9·69 x 10^{-17}	**5·33 x 10^{-16}**

[a] Number of individuals with available genome-wide genotype data included in the estimation of the PRS for airflow limitation; [b] Average age of the individuals at the data collection follow-ups included in this study; [c] Number of individuals with available genotype data and spirometry measurements included in the association analyses; [d] Effect size as the change in z-score of lung function per one z-score increase in the PRS; [e] Adjusted *p*-value accounting for the false discovery rate. The Benjamini & Yekutieli method was applied across spirometry measurements per time point and cohort; [f] z-score of pre-bronchodilator spirometry measurements obtained from applying the Global Lung Function Initiative equations; [g] The PRS for airflow limitation was separately estimated in each of the genotyping Waves of the BAMSE cohort; [h] Genotyping Wave 1 (n=463); [i] Genotyping Wave 2 (n=2,173); [j] Five PCs were included as covariates; [k] Twenty PCs were included as covariates; [l] Regression models were not adjusted by any PCs due to high homogeneity in genetic ancestry among participants. A covariate related to the study group was included; [m] Ten PCs, the participation round, and the genotyping batch were included as covariates; [n] Subjects were classified into groups of similar age for this study; [o] Regression models were adjusted by three PCs to allow an appropriate control of population stratification; [p] Age-specific PRS estimates were obtained given the cross-sectional type of this cohort.

CI: confidence interval; FEV$_1$: forced expiratory volume in one second; FVC: forced vital capacity; PC: Principal Component of genetic ancestry; PRS: polygenic risk score.

Results are shown for the basic association model, including two PCs (or specified otherwise) and any cohort-specific variables as covariates.

Significant results (*q*-value ≤0·05) are highlighted in bold font.

Table S8 (continuation). Results of the association of the PRS for airflow limitation with spirometry measurements up to 50 years of age in each cohort.

				FEV$_1$ [f]			FVC [f]			FEV$_1$/FVC [f]		
**Cohort**	**n_{PRS} [a]**	**Time point [b]**	**$n_{Association}$ [c]**	**β (95%CI) [d]**	***p*-value**	***q*-value [e]**	**β (95%CI) [d]**	***p*-value**	***q*-value [e]**	**β (95%CI) [d]**	***p*-value**	***q*-value [e]**
GINIplus/LISA North	792	10 years	374	-0·02 (-0·10, 0·07)	0·694	1	0·05 (-0·03, 0·14)	0·192	0·528	-0·15 (-0·25, -0·05)	4·02 x 10^{-3}	**0·022**
		15 years	496	-0·16 (-0·24, -0·08)	1·65 x 10^{-4}	**9·08 x 10^{-4}**	-0·07 (-0·16, 0·01)	0·089	0·163	-0·14 (-0·23, -0·06)	1·15 x 10^{-3}	**3·16 x 10^{-3}**
GINIplus/LISA South [l]	1,511	6 years	106	-0·12 (-0·29, 0·04)	0·144	0·396	-0·15 (-0·31, 0·01)	0·074	0·396	0·07 (-0·07, 0·21)	0·338	0·620
		15 years	843	-0·07 (-0·13, -0·01)	0·020	0·054	-0·01 (-0·07, 0·05)	0·796	1	-0·11 (-0·17, -0·04)	1·58 x 10^{-3}	**8·69 x 10^{-3}**
HUNT [m]	69,717	20-30 years [n]	2,848	-0·15 (-0·19, -0·11)	2·03 x 10^{-14}	**5·58 x 10^{-14}**	-0·03 (-0·06, 0·01)	0·148	0·271	-0·18 (-0·21, -0·15)	5·36 x 10^{-26}	**2·95 x 10^{-25}**
		31-40 years [n]	3,107	-0·15 (-0·19, -0·11)	5·65 x 10^{-15}	**1·55 x 10^{-14}**	-0·03 (-0·07, 0·00)	0·054	0·098	-0·18 (-0·21, -0·15)	8·83 x 10^{-26}	**4·86 x 10^{-25}**
		41-50 years [n]	4,142	-0·17 (-0·2, -0·13)	1·63 x 10^{-20}	**4·48 x 10^{-20}**	-0·07 (-0·10, -0·04)	1·22 x 10^{-5}	**2·24 x 10^{-5}**	-0·16 (-0·19, -0·13)	2·38 x 10^{-25}	**1·31 x 10^{-24}**
INMA [o]	2,034	4 years	559	-0·07 (-0·17, 0·02)	0·135	0·424	-0·07 (-0·17, 0·03)	0·154	0·424	-0·02 (-0·09, 0·06)	0·630	1
		7 years	925	-0·08 (-0·14, -0·02)	5·80 x 10^{-3}	**0·016**	0·00 (-0·06, 0·06)	0·983	1	-0·14 (-0·20, -0·08)	1·59 x 10^{-5}	**8·75 x 10^{-5}**
		10 years	65	-0·08 (-0·34, 0·18)	0·568	1	0·01 (-0·26, 0·28)	0·961	1	-0·13 (-0·37, 0·12)	0·312	1
		11 years	792	-0·07 (-0·14, 0·01)	0·074	0·203	0·01 (-0·07, 0·08)	0·880	1	-0·12 (-0·19, -0·05)	9·23 x 10^{-4}	**5·08 x 10^{-3}**
		14 years	188	0·02 (-0·13, 0·16)	0·813	1	0·07 (-0·08, 0·21)	0·366	1	-0·09 (-0·23, 0·05)	0·193	1
		18 years	87	0·00 (-0·22, 0·21)	0·989	1	0·07 (-0·14, 0·29)	0·497	1	-0·12 (-0·34, 0·09)	0·267	1
IoWBC	956	10 years	754	-0·05 (-0·12, 0·02)	0·164	0·451	0·02 (-0·04, 0·09)	0·463	0·849	-0·12 (-0·19, -0·05)	1·05 x 10^{-3}	**5·78 x 10^{-3}**
		18 years	669	-0·15 (-0·22, -0·07)	1·17 x 10^{-4}	**6·44 x 10^{-4}**	-0·04 (-0·11, 0·03)	0·316	0·579	-0·15 (-0·23, -0·06)	4·59 x 10^{-4}	**1·26 x 10^{-3}**
		26 years	432	-0·04 (-0·14, 0·05)	0·355	0·976	0·02 (-0·06, 0·11)	0·589	1	-0·09 (-0·18, 0·00)	0·044	0·242
Lifelines	859 [p]	18-30 years [n]	859	-0·12 (-0·17, -0·06)	1·47 x 10^{-4}	**4·04 x 10^{-4}**	-0·01 (-0·07, 0·05)	0·753	1	-0·17 (-0·23, -0·10)	3·25 x 10^{-7}	**1·79 x 10^{-6}**
	3,005 [p]	31-40 years [n]	3,005	-0·12 (-0·15, -0·08)	2·47 x 10^{-11}	**6·79 x 10^{-11}**	-0·02 (-0·05, 0·01)	0·199	0·365	-0·15 (-0·19, -0·12)	5·53 x 10^{-19}	**3·04 x 10^{-18}**

[a] Number of individuals with available genome-wide genotype data included in the estimation of the PRS for airflow limitation; [b] Average age of the individuals at the data collection follow-ups included in this study; [c] Number of individuals with available genotype data and spirometry measurements included in the association analyses; [d] Effect size as the change in z-score of lung function per one z-score increase in the PRS; [e] Adjusted *p*-value accounting for the false discovery rate. The Benjamini & Yekutieli method was applied across spirometry measurements per time point and cohort; [f] z-score of pre-bronchodilator spirometry measurements obtained from applying the Global Lung Function Initiative equations; [g] The PRS for airflow limitation was separately estimated in each of the genotyping Waves of the BAMSE cohort; [h] Genotyping Wave 1 (n=463); [i] Genotyping Wave 2 (n=2,173); [j] Five PCs were included as covariates; [k] Twenty PCs were included as covariates; [l] Regression models were not adjusted by any PCs due to high homogeneity in genetic ancestry among participants. A covariate related to the study group was included; [m] Ten PCs, the participation round, and the genotyping batch were included as covariates; [n] Subjects were classified into groups of similar age for this study; [o] Regression models were adjusted by three PCs to allow an appropriate control of population stratification; [p] Age-specific PRS estimates were obtained given the cross-sectional type of this cohort.

CI: confidence interval; FEV$_1$: forced expiratory volume in one second; FVC: forced vital capacity; PC: Principal Component of genetic ancestry; PRS: polygenic risk score.

Results are shown for the basic association model, including two PCs (or specified otherwise) and any cohort-specific variables as covariates.

Significant results (*q*-value ≤0·05) are highlighted in bold font.

Table S8 (continuation). Results of the association of the PRS for airflow limitation with spirometry measurements up to 50 years of age in each cohort.

				FEV_1 [f]			FVC [f]			FEV_1/FVC [f]		
**Cohort**	**n_{PRS} [a]**	**Time point [b]**	**$n_{Association}$ [c]**	**β (95%CI) [d]**	***p*-value**	***q*-value [e]**	**β (95%CI) [d]**	***p*-value**	***q*-value [e]**	**β (95%CI) [d]**	***p*-value**	***q*-value [e]**
MAAS	852	8 years	640	-0·12 (-0·20, -0·04)	3·85 x 10-3	**0·011**	-0·04 (-0·12, 0·03)	0·245	0·449	-0·12 (-0·20, -0·05)	1·16 x 10-3	**6·38 x 10^{-3}**
		16 years	502	-0·09 (-0·18, 0·00)	0·045	0·123	0·00 (-0·09, 0·09)	0·996	1	-0·17 (-0·27, -0·08)	2·32 x 10-4	**1·28 x 10^{-3}**
		19 years	436	-0·11 (-0·20, -0·02)	0·022	0·061	-0·02 (-0·10, 0·07)	0·664	1	-0·15 (-0·25, -0·05)	3·46 x 10-3	**0·019**
PIAMA	1,526	8 years	907	-0·09 (-0·15, -0·03)	1·85 x 10-3	**7·62 x 10^{-3}**	-0·03 (-0·09, 0·03)	0·331	0·607	-0·11 (-0·18, -0·04)	2·77 x 10-3	**7·62 x 10^{-3}**
		12 years	1,018	-0·06 (-0·11, -0·01)	0·028	0·077	0·01 (-0·05, 0·06)	0·782	1	-0·12 (-0·18, -0·07)	1·83 x 10-5	**1·01 x 10^{-4}**
		16 years	653	-0·10 (-0·17, -0·03)	3·39 x 10-3	**9·32 x 10^{-3}**	-0·02 (-0·09, 0·04)	0·442	0·810	-0·12 (-0·19, -0·04)	1·68 x 10-3	**9·24 x 10^{-3}**
SEATON	552	10 years	382	-0·16 (-0·27, -0·06)	2·15 x 10-3	**0·012**	-0·10 (-0·19, 0·00)	0·055	0·102	-0·11 (-0·20, -0·01)	0·024	0·067
		15 years	330	-0·01 (-0·12, 0·09)	0·821	1	0·07 (-0·04, 0·18)	0·188	0·517	-0·18 (-0·29, -0·06)	2·74 x 10-3	**0·015**

[a] Number of individuals with available genome-wide genotype data included in the estimation of the PRS for airflow limitation; [b] Average age of the individuals at the data collection follow-ups included in this study; [c] Number of individuals with available genotype data and spirometry measurements included in the association analyses; [d] Effect size as the change in z-score of lung function per one z-score increase in the PRS; [e] Adjusted *p*-value accounting for the false discovery rate. The Benjamini & Yekutieli method was applied across spirometry measurements per time point and cohort; [f] z-score of pre-bronchodilator spirometry measurements obtained from applying the Global Lung Function Initiative equations; [g] The PRS for airflow limitation was separately estimated in each of the genotyping Waves of the BAMSE cohort; [h] Genotyping Wave 1 (n=463); [i] Genotyping Wave 2 (n=2,173); [j] Five PCs were included as covariates; [k] Twenty PCs were included as covariates; [l] Regression models were not adjusted by any PCs due to high homogeneity in genetic ancestry among participants. A covariate related to the study group was included; [m] Ten PCs, the participation round, and the genotyping batch were included as covariates; [n] Subjects were classified into groups of similar age for this study; [o] Regression models were adjusted by three PCs to allow an appropriate control of population stratification; [p] Age-specific PRS estimates were obtained given the cross-sectional type of this cohort.

CI: confidence interval; FEV_1: forced expiratory volume in one second; FVC: forced vital capacity; PC: Principal Component of genetic ancestry; PRS: polygenic risk score.

Results are shown for the basic association model, including two PCs (or specified otherwise) and any cohort-specific variables as covariates.

Significant results (*q*-value ≤0·05) are highlighted in bold font.

Table S9. Comparison of the results from the age-stratified main and alternative meta-analyses differing in the age groups with more than one time point per cohort.

		Main meta-analysis					***Alternative meta-analysis***				
**Age group**	**Spirometry measurement [a]**	**Cohorts included**	**Sample size [c]**	**β (95%CI) [d]**	***p*-value [e]**	***q*-value [f]**	**Cohorts included**	**Sample size [c]**	**β (95%CI) [d]**	***p*-value [e]**	***q*-value [f]**
School age (7-10 years)	FEV_1	COPSAC2000, 7<br>**INMA, 7**<br>ALSPAC, 8<br>BAMSE-W1, 8 [b]<br>BAMSE-W2, 8 [b]<br>MAAS, 8<br>PIAMA, 8<br>Generation R, 9<br>COPSAC2010, 10<br>GINIplus/LISA North, 10<br>IoWBC, 10<br>SEATON, 10	13,387	-0·07<br>(-0·09, -0·05)	5·99 x 10^{-10}	**1·65 x 10^{-9}**	COPSAC2000, 7<br>ALSPAC, 8<br>BAMSE-W1, 8 [b]<br>BAMSE-W2, 8 [b]<br>MAAS, 8<br>PIAMA, 8<br>Generation R, 9<br>COPSAC2010, 10<br>GINIplus/LISA North, 10<br>**INMA, 10**<br>IoWBC, 10<br>SEATON, 10	12,527	-0·07<br>(-0·09, -0·05)	1·22 x 10^{-8}	**3·36 x 10^{-8}**
	FVC			0·00<br>(-0·02, 0·01)	0·752	1			0·00<br>(-0·02, 0·01)	0·741	1
	FEV_1/FVC			-0·13<br>(-0·15, -0·11)	1·28 x 10^{-53}	**7·04 x 10^{-53}**			-0·13<br>(-0·15, -0·11)	9·42 x 10^{-50}	**5·18 x 10^{-49}**
Puberty (11-15 years)	FEV_1	**INMA, 11**<br>COPSAC2000, 12<br>PIAMA, 12<br>Generation R, 13<br>ALSPAC, 15<br>Ashford, 15<br>GINIplus/LISA North, 15<br>GINIplus/LISA South, 15<br>SEATON, 15	9,323	-0·07<br>(-0·10, -0·05)	2·58 x 10^{-7}	**7·10 x 10^{-7}**	COPSAC2000, 12<br>PIAMA, 12<br>Generation R, 13<br>**INMA, 14**<br>ALSPAC, 15<br>Ashford, 15<br>GINIplus/LISA North, 15<br>GINIplus/LISA South, 15<br>SEATON, 15	8,719	-0·07<br>(-0·10, -0·04)	2·04 x 10^{-5}	**5·61 x 10^{-5}**
	FVC			0·00<br>(-0·02, 0·02)	0·728	1			0·00<br>(-0·02, 0·03)	0·655	1
	FEV_1/FVC			-0·14<br>(-0·16, -0·12)	1·18 x 10^{-40}	**6·49 x 10^{-40}**			-0·14<br>(-0·16, -0·12)	1·54 x 10^{-36}	**8·47 x 10^{-36}**
Adulthood (18-30 years)	FEV_1	COPSAC2000, 18<br>INMA, 18<br>**IoWBC, 18**<br>Lifelines, 18-30<br>MAAS, 19<br>HUNT, 20-30<br>ALSPAC, 24<br>BAMSE-W1, 24 [b]<br>BAMSE-W2, 24 [b]	9,132	-0·12<br>(-0·14, -0·09)	6·94 x 10^{-25}	**1·91 x 10^{-24}**	COPSAC2000, 18<br>INMA, 18<br>Lifelines, 18-30<br>MAAS, 19<br>HUNT, 20-30<br>ALSPAC, 24<br>BAMSE-W1, 24 [b]<br>BAMSE-W2, 24 [b]<br>**IoWBC, 26**	8,895	-0·11<br>(-0·13, -0·09)	1·02 x 10^{-18}	**2·81 x 10^{-18}**
	FVC			-0·01<br>(-0·03, 0·01)	0·280	0·513			-0·01<br>(-0·03, 0·01)	0·481	0·882
	FEV_1/FVC			-0·16<br>(-0·18, -0·14)	1·06 x 10^{-57}	**5·83 x 10^{-57}**			-0·16<br>(-0·18, -0·13)	7·61 x 10^{-49}	**4·19 x 10^{-48}**

[a] Z-score of pre-bronchodilator spirometry measurements obtained from applying the Global Lung Function Initiative equations; [b] The PRS for airflow limitation was separately estimated in each of the genotyping Waves of the BAMSE cohort (Wave 1, n=463; Wave 2, n=2,173); [c] Number of individuals with available genotype data and spirometry measurements included in the association analyses; [d] Effect size of the association as the change in z-score of lung function per one z-score increase in the PRS; [e] A random-effects model was applied to account for the heterogeneity across studies regardless of the significance of the Cochran Q-test and I^2 estimate; [f] Adjusted *p*-value accounting for the false discovery rate. The Benjamini & Yekutieli method was applied across spirometry measurements per age group.

CI: confidence interval; FEV_1: forced expiratory volume in one second; FVC: forced vital capacity; W1: Genotyping Wave 1; W2: Genotyping Wave 2. Results are shown for the basic association model, including Principal Components of genetic ancestry and any cohort-specific variables as covariates. Main meta-analysis: original composition of the groups for the meta-analysis based on age similarity. Alternative meta-analysis: alternative composition of the groups for the meta-analysis based on age similarity replacing the time points discarded. The time points replaced are highlighted with bold font among the studies included. Significant results (*q*-value ≤0·05) are highlighted in bold font.

Table S10. Results of the validation of the association with spirometry measurements in participants from the Rotterdam Study.

Association model	Time point	Spirometry measurement [a]	Sample size [b]	β (95%CI) [c]	*p*-value	*q*-value [d]
Basic [e]	PFT 1 (50-98 years) [h]	FEV_1	5,722	-0·15 (-0·18, -0·12)	2·14 x 10^{-20}	**5·89 x 10^{-20}**
		FVC		-0·07 (-0·10, -0·04)	3·10 x 10^{-6}	**5·68 x 10^{-6}**
		FEV_1/FVC		-0·14 (-0·17, -0·11)	4·32 x 10^{-24}	**2·38 x 10^{-23}**
	PFT 2 (51-96 years) [h]	FEV_1	3,317	-0·14 (-0·17, -0·10)	3·34 x 10^{-12}	**9·19 x 10^{-12}**
		FVC		-0·05 (-0·08, -0·01)	7·05 x 10^{-3}	**0·013**
		FEV_1/FVC		-0·15 (-0·19, -0·12)	9·66 x 10^{-22}	**5·31 x 10^{-21}**
	PFT 3 (70-100 years) [h]	FEV_1	741	-0·17 (-0·25, -0·09)	3·95 x 10^{-5}	**1·09 x 10^{-4}**
		FVC		-0·05 (-0·12, 0·02)	0·144	0·264
		FEV_1/FVC		-0·19 (-0·26, -0·13)	1·71 x 10^{-8}	**9·41 x 10^{-8}**
Sensitivity – Smoking status [f]	PFT 1 (50-98 years) [h]	FEV_1	5,672	-0·15 (-0·18, -0·12)	5·47 x 10^{-21}	**1·50 x 10^{-20}**
		FVC		-0·07 (-0·10, -0·04)	3·01 x 10^{-6}	**5·52 x 10^{-6}**
		FEV_1/FVC		-0·14 (-0·17, -0·11)	5·86 x 10^{-25}	**3·22 x 10^{-24}**
	PFT 2 (51-96 years) [h]	FEV_1	3,317	-0·13 (-0·17, -0·09)	2·03 x 10^{-11}	**5·58 x 10^{-11}**
		FVC		-0·04 (-0·07, -0·01)	0·012	**0·021**
		FEV_1/FVC		-0·15 (-0·18, -0·12)	9·10 x 10^{-21}	**5·01 x 10^{-20}**
	PFT 3 (70-100 years) [h]	FEV_1	739	-0·16 (-0·24, -0·08)	6·90 x 10^{-5}	**1·90 x 10^{-4}**
		FVC		-0·05 (-0·12, 0·02)	0·162	0·297
		FEV_1/FVC		-0·18 (-0·25, -0·12)	3·51 x 10^{-8}	**1·93 x 10^{-7}**
Sensitivity – Smoking pack-years [g]	PFT 1 (50-98 years) [h]	FEV_1	263	-0·18 (-0·31, -0·06)	3·94 x 10^{-3}	**0·022**
		FVC		-0·08 (-0·18, 0·02)	0·109	0·200
		FEV_1/FVC		-0·17 (-0·30, -0·04)	9·12 x 10^{-3}	**0·025**
	PFT 2 (51-96 years) [h]	FEV_1	342	-0·16 (-0·29, -0·04)	0·011	**0·029**
		FVC		-0·05 (-0·16, 0·06)	0·400	0·733
		FEV_1/FVC		-0·22 (-0·34, -0·11)	1·85 x 10^{-4}	**1·02 x 10^{-3}**
	PFT 3 (70-100 years) [h]	FEV_1	45	-0·64 (-0·97, -0·31)	4·78 x 10^{-4}	**1·31 x 10^{-3}**
		FVC		-0·37 (-0·70, -0·05)	0·030	0·055
		FEV_1/FVC		-0·58 (-0·85, -0·31)	1·25 x 10^{-4}	**6·88 x 10^{-4}**

[a] z-score of pre-bronchodilator spirometry measurements obtained from applying the Global Lung Function Initiative equations; [c] Number of individuals with available genotype data and spirometry measurements included in the association analyses; [c] Effect size of the association as the change in z-score of lung function per one z-score increase in the PRS; [d] Adjusted p-value accounting for the false discovery rate. The Benjamini & Yekutieli method was applied across spirometry measurements per time point and association model; [e] Basic association model, including two PCs as covariates; [f] Sensitivity analyses accounting for active smoking. Two PCs and smoking status were included as covariates; [g] Sensitivity analyses accounting for active smoking restricted to subjects with reported smoking habits. Two PCs and tobacco pack-years were included as covariates; [h] Participants were grouped based on the time point when the pulmonary function test was conducted.

CI: confidence interval; FEV_1: forced expiratory volume in one second; FVC: forced vital capacity; PC: Principal Component of genetic ancestry. The results shown correspond to the evaluation of the association of the PRS for airflow limitation with spirometry measurements in each of the three time points when the pulmonary function test was conducted in the Rotterdam Study: PFT 1 (50-98 years), PFT 2 (51-96 years), and PFT 3 (70-100 years). The basic and active smoking-sensitivity association models were applied to the whole set of subjects.

Significant results (*q*-value ≤0·05) are highlighted in bold font.

Table S11. Variance in spirometry measurements explained by the PRS for airflow limitation in each cohort across age groups.

				FEV_1 [a]		FVC [a]		FEV_1/FVC [a]	
**Age group**	**Cohort**	**Time point** [c]	**Sample size** [d]	**R2** [e]	**RMSE** [f]	**R2** [e]	**RMSE** [f]	**R2** [e]	**RMSE** [f]
Preschool age (0-6 years)	INMA	4 years	559	0·030	1·213	0·022	1·270	0·020	0·964
School age (7-10 years)	ALSPAC	8 years	4,871	0·005	1·007	0·002	1·015	0·015	1·059
	BAMSE-W1 [b]	8 years	335	0·063	0·870	0·023	0·861	0·065	0·869
	BAMSE-W2 [b]	8 years	1,230	0·023	0·936	0·005	0·905	0·032	0·876
	PIAMA	8 years	907	0·014	0·904	0·022	0·885	0·052	1·066
Puberty (11-15 years)	PIAMA	12 years	1,018	0·014	0·862	0·010	0·862	0·022	0·882
	ALSPAC	15 years	3,332	0·004	1·264	0·003	1·240	0·019	1·170
Post-puberty (16-17 years)	BAMSE-W1 [b]	16 years	305	0·025	0·888	0·024	0·884	0·032	1·012
	BAMSE-W2 [b]	16 years	1,185	0·028	0·945	0·008	0·937	0·036	0·933
	PIAMA	16 years	653	0·023	0·878	0·025	0·819	0·037	0·931
Adulthood (18-30 years)	HUNT	20-30 years [g]	2,848	0·021	1·030	0·006	0·921	0·037	0·884
	ALSPAC	24 years	2,590	0·011	0·971	0·004	0·946	0·024	0·930
	BAMSE-W1 [b]	24 years	282	0·030	0·803	0·013	0·787	0·051	0·873
	BAMSE-W2 [b]	24 years	1,044	0·025	0·869	0·010	0·872	0·030	0·877
Adulthood (31-40 years)	HUNT	31-40 years [g]	3,107	0·017	1·102	0·004	0·975	0·038	0·953
Adulthood (41-50 years)	HUNT	41-50 years [g]	4,142	0·024	1·189	0·014	1·019	0·031	1·023
Adulthood (>50 years)	HUNT	>50 years [g]	9,027	0·027	1·328	0·011	1·073	0·030	1·216

[a] Z-score of pre-bronchodilator spirometry measurements obtained from applying the Global Lung Function Initiative equations; [b] The PRS for airflow limitation was separately estimated in each of the genotyping Waves of the BAMSE cohort (Wave 1, n=463; Wave 2, n=2,173); [c] Average age of the individuals at the data collection follow-ups included in this study; [d] Number of individuals with available genotype data and spirometry measurements included in the association analyses; [e] Proportion of variance in the spirometry measurement explained by the PRS for airflow limitation in linear regression models (mean); [f] Distance between the predicted and actual values of lung function as an indication of the absolute fit of the model (mean); [g] Subjects were classified into groups of similar age for this study.

FEV_1: forced expiratory volume in one second; FVC: forced vital capacity; PRS: polygenic risk score; RMSE: root mean square error; W1: Genotyping Wave 1; W2: Genotyping Wave 2. Results are shown from the prediction of the performance of each regression model through cross-validation. This was independently carried out in a selected number of studies with the largest sample size per age group as a representation of the participating cohorts. Results are shown for the variance in spirometry measurements by the PRS for airflow limitation applying the basic association model (two Principal Components of genetic ancestry and any cohort-specific variables were included as covariates). Mean values of both R2 and RMSE are reported.

Table S12. Results of sensitivity analyses of the association between the PRS for airflow limitation and spirometry measurements accounting for active smoking status in adults aged 18 to 50 years per cohort.

				FEV$_1$ [f]			FVC [f]			FEV$_1$/FVC [f]		
**Cohort**	**n_{PRS} [a]**	**Time point [b]**	**$n_{Association}$ [c]**	**β (95%CI) [d]**	***p*-value**	***q*-value [e]**	**β (95%CI) [d]**	***p*-value**	***q*-value [e]**	**β (95%CI) [d]**	***p*-value**	***q*-value [e]**
ALSPAC	8,943	24 years	1,862	-0·08 (-0·12, -0·03)	6·04 x 10^{-4}	**1·66 x 10^{-3}**	0·00 (-0·04, 0·05)	0·887	1	-0·12 (-0·16, -0·08)	3·39 x 10^{-8}	**1·86 x 10^{-7}**
BAMSE [g]	463 [h]	24 years	280	-0·09 (-0·18, 0·01)	0·071	0·196	0·02 (-0·07, 0·12)	0·598	1	-0·17 (-0·27, -0·06)	1·69 x 10^{-3}	**9·30 x 10^{-3}**
	2,173 [i]	24 years	1,031	-0·12 (-0·18, -0·07)	5·99 x 10^{-6}	**1·65 x 10^{-5}**	-0·02 (-0·07, 0·04)	0·554	1	-0·15 (-0·21, -0·10)	2·71 x 10^{-8}	**1·49 x 10^{-7}**
COPSAC2000 [j]	358	18 years	317	-0·06 (-0·17, 0·05)	0·288	0·528	0·08 (-0·02, 0·19)	0·132	0·363	-0·19 (-0·30, -0·09)	4·14 x 10^{-4}	**2·28 x 10^{-3}**
		20-30 years [l]	2,800	-0·14 (-0·18, -0·10)	4·65 x 10^{-13}	**1·28 x 10^{-12}**	-0·02 (-0·05, 0·01)	0·263	0·482	-0·18 (-0·21, -0·14)	4·11 x 10^{-25}	**2·26 x 10^{-4}**
HUNT [k]	69,717	31-40 years [l]	3,068	-0·15 (-0·19, -0·11)	3·72 x 10^{-14}	**1·02 x 10^{-13}**	-0·03 (-0·06, 0·00)	0·077	0·141	-0·18 (-0·21, -0·14)	5·51 x 10^{-25}	**3·03 x 10^{-4}**
		41-50 years [l]	4,103	-0·16 (-0·20, -0·13)	9·99 x 10^{-20}	**2·75 x 10^{-19}**	-0·06 (-0·09, -0·03)	3·36 x 10^{-5}	**6·16 x 10^{-5}**	-0·16 (-0·19, -0·13)	3·65 x 10^{-25}	**2·01 x 10^{-4}**
INMA [m]	2,034	18 years	29	0·04 (-0·36, 0·44)	0·853	1	0·05 (-0·38, 0·48)	0·828	1	-0·05 (-0·51, 0·41)	0·845	1
IoWBC	956	18 years	669	-0·15 (-0·22, -0·07)	1·06 x 10^{-4}	**5·83 x 10^{-4}**	-0·04 (-0·11, 0·04)	0·327	0·600	-0·15 (-0·23, -0·07)	3·65 x 10^{-4}	**1·00 x 10^{-3}**
Lifelines	859 [n]	18-30 years [l]	840	-0·12 (-0·18, -0·06)	1·02 x 10^{-4}	**2·81 x 10^{-4}**	-0·01 (-0·07, 0·05)	0·822	1	-0·18 (-0·24, -0·11)	8·09 x 10^{-8}	**4·45 x 10^{-7}**
	3,005 [n]	31-40 years [l]	2,947	-0·11 (-0·15, -0·08)	5·57 x 10^{-11}	**1·53 x 10^{-10}**	-0·02 (-0·05, 0·01)	0·229	0·420	-0·15 (-0·19, -0·12)	6·46 x 10^{-19}	**3·55 x 10^{-8}**
MAAS	852	19 years	436	-0·11 (-0·20, -0·01)	0·023	0·063	-0·02 (-0·10, 0·07)	0·663	1	-0·15 (-0·25, -0·05)	3·65 x 10^{-3}	**0·020**

[a] Number of individuals with available genome-wide genotype data included in the estimation of the PRS for airflow limitation; [b] Average age of the individuals at the data collection follow-ups included in this study; [c] Number of individuals with available genotype data, spirometry measurements, and smoking status information included in the association analyses; [d] Effect size of the association as the change in z-score of lung function per one z-score increase in the PRS; [e] Adjusted p-value accounting for the false discovery rate. The Benjamini & Yekutieli method was applied across spirometry measurements per time point and cohort; [f] Z-score of pre-bronchodilator spirometry measurements obtained from applying the Global Lung Function Initiative equations; [g] The PRS for airflow limitation was separately estimated in each of the genotyping Waves of the BAMSE cohort; [h] Genotyping Wave 1; [i] Genotyping Wave 2; [j] Five PCs were included as covariates; [k] Ten PCs, the participation round and the genotyping batch were included as covariates; [l] Subjects were classified into groups of similar age for this study; [m] Regression models were adjusted by three PCs to allow appropriate control of population stratification; [n] Age-specific PRS estimates were obtained given the cross-sectional type of this cohort.

CI: confidence interval; FEV$_1$: forced expiratory volume in one second; FVC: forced vital capacity; PC: Principal Component of genetic ancestry; PRS: polygenic risk score.

Results are shown for the association analyses of PRS for airflow limitation with spirometry measurements adjusted by two PCs (or specified otherwise), any cohort-specific variables, and smoking status.

Significant results (*q*-value ≤0·05) are highlighted in bold font.

Table S13. Results of association analyses of the PRS for airflow limitation with spirometry measurements in subjects aged 18 to 50 years with reported smoking habits, including a covariate of tobacco pack-years in each cohort.

				FEV_1 [f]			FVC [f]			FEV_1/FVC [f]		
**Cohort**	**n_{PRS} [a]**	**Time point [b]**	**$n_{Association}$ [c]**	**β (95%CI) [d]**	***p*-value**	***q*-value [e]**	**β (95%CI) [d]**	***p*-value**	***q*-value [e]**	**β (95%CI) [d]**	***p*-value**	***q*-value [e]**
BAMSE [g]	463 [h]	24 years	45	0·01 (-0·31, 0·34)	0·929	1	0·11 (-0·17, 0·38)	0·440	1	-0·12 (-0·47, 0·23)	0·51	1
	2,173 [i]	24 years	173	-0·15 (-0·26, -0·03)	**0·012**	0·033	-0·02 (-0·14, 0·09)	0·689	1	-0·17 (-0·28, -0·06)	**$2·56 \times 10^{-3}$**	**0·014**
COPSAC2000 [j]	358	18 years	55	0·02 (-0·18, 0·21)	0·876	1	0·07 (-0·12, 0·26)	0·490	1	-0·07 (-0·31, 0·18)	0·585	1
HUNT [k]	69,717	20-30 years [l]	867	-0·14 (-0·21, -0·07)	**$7·32 \times 10^{-5}$**	**$2·01 \times 10^{-4}$**	-0·03 (-0·09, 0·04)	0·406	0·744	-0·16 (-0·22, -0·10)	**$2·08 \times 10^{-7}$**	**$1·14 \times 10^{-6}$**
		31-40 years [l]	1,010	-0·17 (-0·24, -0·10)	**$2·75 \times 10^{-6}$**	**$7·56 \times 10^{-6}$**	-0·04 (-0·10, 0·02)	0·213	0·391	-0·19 (-0·25, -0·13)	**$1·94 \times 10^{-9}$**	**$1·07 \times 10^{-8}$**
		41-50 years [l]	1,392	-0·12 (-0·18, -0·06)	**$1·49 \times 10^{-4}$**	**$4·10 \times 10^{-4}$**	0·00 (-0·05, 0·05)	0·956	1	-0·19 (-0·25, -0·14)	**$1·99 \times 10^{-11}$**	**$1·09 \times 10^{-10}$**
Lifelines	859 [m]	18-30 years [l]	277	-0·16 (-0·26, -0·06)	**$2·71 \times 10^{-3}$**	**$7·45 \times 10^{-3}$**	-0·05 (-0·15, 0·05)	0·366	0·671	-0·19 (-0·30, -0·08)	**$8·01 \times 10^{-4}$**	**$4·41 \times 10^{-3}$**
	3,005 [m]	31-40 years [l]	724	-0·12 (-0·19, -0·05)	**$4·09 \times 10^{-4}$**	**$1·12 \times 10^{-3}$**	-0·03 (-0·09, 0·04)	0·426	0·781	-0·15 (-0·22, -0·09)	**$1·00 \times 10^{-5}$**	**$5·50 \times 10^{-5}$**

[a] Number of individuals with available genome-wide genotype data included in the estimation of the PRS for airflow limitation; [b] Average age of the individuals at the data collection follow-ups included in this study; [c] Number of individuals with available genotype data, spirometry measurements, and smoking pack-years information included in the association analyses. Only subjects with reported active smoking habits were included; [d] Effect size of the association as the change in z-score of lung function per one z-score increase in the PRS; [e] Adjusted *p*-value accounting for the false discovery rate. The Benjamini & Yekutieli method was applied across spirometry measurements per time point and cohort; [f] Z-score of pre-bronchodilator spirometry measurements obtained from applying the Global Lung Function Initiative equations; [g] The PRS for airflow limitation was separately estimated in each of the genotyping Waves of BAMSE; [h] Genotyping Wave 1; [i] Genotyping Wave 2; [j] Five PCs were included as covariates; [k] Ten PCs, the participation round and the genotyping batch were included as covariates; [l] Subjects were classified into groups of similar age for this study; [m] Age-specific PRS estimates were obtained given the cross-sectional type of this cohort.

CI: confidence interval; FEV_1: forced expiratory volume in one second; FVC: forced vital capacity; PC: Principal Component of genetic ancestry; PRS: polygenic risk score.

Results are shown for association analyses of PRS for airflow limitation with spirometry measurements adjusted by two PCs (or specified otherwise), any cohort-specific variables, and tobacco pack-years. Tobacco pack-years were calculated by multiplying the number of smoking years by the number of daily cigarettes and dividing by 20 cigarettes often contained in a package.

Significant results (*q*-value ≤0·05) are highlighted in bold font.

Table S14. Results of the meta-analysis of association results of the PRS for airflow limitation with spirometry measurements by age groups up to 50 years stratified by sex.

		Males				***Females***			
**Age group**	**Spirometry measurement** [a]	**Sample size** [b]	**β (95%CI)** [c]	***p*-value** [d]	***q*-value** [e]	**Sample size** [b]	**β (95%CI)** [c]	***p*-value** [d]	***q*-value** [e]
	FEV$_1$		-0·14 (-0·25, -0·02)	0·021	0·058		-0·03 (-0·14, 0·09)	0·630	1
Preschool age (0-6 years)	FVC	340	-0·14 (-0·26, -0·02)	0·019	0·058	325	-0·04 (-0·16, 0·08)	0·495	1
	FEV$_1$/FVC		0·00 (-0·09, 0·08)	0·961	1		0·00 (-0·10, 0·10)	0·980	1
	FEV$_1$		-0·08 (-0·11, -0·06)	1·64 x 10^{-10}	**4·51 x 10^{-10}**		-0·06 (-0·10, -0·02)	1·87 x 10^{-3}	**5·14 x 10^{-3}**
School age (7-10 years)	FVC	6,714	-0·01 (-0·03, 0·02)	0·543	0·996	6,673	0·00 (-0·04, 0·03)	0·916	1
	FEV$_1$/FVC		-0·15 (-0·17, -0·13)	5·70 x 10^{-35}	**3·14 x 10^{-34}**		-0·11 (-0·14, -0·08)	1·26 x 10^{-14}	**6·93 x 10^{-14}**
	FEV$_1$		-0·08 (-0·13, -0·03)	1·67 x 10^{-3}	**4·59 x 10^{-3}**		-0·08 (-0·11, -0·05)	7·63 x 10^{-8}	**2·10 x 10^{-7}**
Puberty (11-15 years)	FVC	4,516	0·01 (-0·03, 0·06)	0·633	1	4,807	-0·01 (-0·03, 0·02)	0·647	1
	FEV$_1$/FVC		-0·15 (-0·18, -0·12)	3·35 x 10^{-24}	**1·84 x 10^{-23}**		-0·13 (-0·16, -0·10)	1·47 x 10^{-19}	**8·09 x 10^{-19}**
	FEV$_1$		-0·13 (-0·18, -0·08)	1·34 x 10^{-6}	**3·69 x 10^{-6}**		-0·08 (-0·16, 0·01)	0·067	0·184
Post-puberty (16-17 years)	FVC	1,259	-0·01 (-0·06, 0·04)	0·649	1	1,386	-0·01 (-0·06, 0·03)	0·610	1
	FEV$_1$/FVC		-0·17 (-0·23, -0·12)	7·21 x 10^{-10}	**3·97 x 10^{-9}**		-0·14 (-0·19, -0·09)	6·86 x 10^{-8}	**3·77 x 10^{-7}**
	FEV$_1$		-0·13 (-0·17, -0·10)	2·58 x 10^{-14}	**7·10 x 10^{-14}**		-0·10 (-0·13, -0·06)	1·20 x 10^{-6}	**3·30 x 10^{-6}**
Adulthood (18-30 years)	FVC	3,936	-0·01 (-0·04, 0·02)	0·559	1	5,196	-0·01 (-0·04, 0·01)	0·352	0·645
	FEV$_1$/FVC		-0·18 (-0·21, -0·15)	1·12 x 10^{-32}	**6·16 x 10^{-32}**		-0·14 (-0·17, -0·12)	9·10 x 10^{-28}	**5·01 x 10^{-27}**
	FEV$_1$		-0·12 (-0·16, -0·08)	2·95 x 10^{-10}	**8·11 x 10^{-10}**		-0·14 (-0·19, -0·09)	1·50 x 10^{-9}	**8·25 x 10^{-9}**
Adulthood (31-40 years)	FVC	2,631	-0·02 (-0·05, 0·02)	0·315	0·578	3,481	-0·03 (-0·07, 0·00)	0·033	0·060
	FEV$_1$/FVC		-0·17 (-0·21, -0·13)	4·24 x 10^{-19}	**2·33 x 10^{-18}**		-0·16 (-0·22, -0·11)	1·65 x 10^{-8}	**4·54 x 10^{-8}**
	FEV$_1$		-0·16 (-0·21, -0·11)	3·82 x 10^{-9}	**1·05 x 10^{-8}**		-0·18 (-0·22, -0·13)	4·21 x 10^{-13}	**1·16 x 10^{-12}**
Adulthood (41-50 years) [f]	FVC	1,904	-0·06 (-0·11, -0·02)	5·29 x 10^{-3}	**9·70 x 10^{-3}**	2,238	-0·07 (-0·11, -0·03)	6·83 x 10^{-4}	**1·25 x 10^{-3}**
	FEV$_1$/FVC		-0·16 (-0·21, -0·11)	3·75 x 10^{-11}	**2·06 x 10^{-10}**		-0·17 (-0·21, -0·13)	4·06 x 10^{-16}	**2·23 x 10^{-15}**

[a] Z-score of pre-bronchodilator spirometry measurements obtained from applying the Global Lung Function Initiative equations; [b] Number of individuals with available genotype data and lung function information included in the association analyses stratified by sex; [c] Effect size of the association as the change in z-score of lung function per one z-score increase in the PRS; [d] A random-effects model was applied to account for the heterogeneity across studies regardless of the significance of the Cochran Q-test and I^2 estimate; [e] Adjusted *p*-value accounting for the false discovery rate. The Benjamini & Yekutieli method was applied across spirometry measurements per age group; [f] The results shown correspond only to the association testing with spirometry measurements in the HUNT cohort given the absence of more cohorts with available spirometry measurements within that age range.

CI: confidence interval; FEV$_1$: forced expiratory volume in one second; FVC: forced vital capacity; PRS: polygenic risk score.

Results are shown for the age-stratified meta-analysis of the participating cohorts with subjects up to 50 years of age, separately conducted in males and females. The basic association model was applied, including Principal Components of genetic ancestry, and any cohort-specific variables as covariates.

Significant results (*q*-value ≤0·05) are highlighted in bold font.

Table S15. Results of the association testing of the PRS for airflow limitation with spirometry measurements stratified by sex in the HUNT cohort (>50 years of age).

		Males				***Females***			
**Association model**	**Spirometry measurement** [a]	**Sample size** [b]	**β (95%CI)** [c]	***p*-value**	***q*-value** [d]	**Sample size** [b]	**β (95%CI)** [c]	***p*-value**	***q*-value** [d]
Basic [e]	FEV_1	4,275	-0·21 (-0·25, -0·17)	1·00 x 10^{-23}	**2·75 x 10^{-23}**	4,752	-0·12 (-0·16, -0·09)	6·23 x 10^{-11}	**1·71 x 10^{-10}**
	FVC	4,275	-0·08 (-0·12, -0·05)	4·60 x 10^{-7}	**8·43 x 10^{-7}**	4,752	-0·06 (-0·09, -0·03)	1·41 x 10^{-4}	**2·59 x 10^{-4}**
	FEV_1/FVC	4,275	-0·22 (-0·26, -0·18)	1·61 x 10^{-30}	**8·86 x 10^{-30}**	4,752	-0·12 (-0·15, -0·09)	4·35 x 10^{-13}	**2·39 x 10^{-12}**
Sensitivity - Smoking status [f]	FEV_1	4,166 [h]	-0·20 (-0·24, -0·16)	2·44 x 10^{-23}	**6·71 x 10^{-23}**	4,618 [h]	-0·12 (-0·16, -0·09)	4·69 x 10^{-11}	**1·29 x 10^{-10}**
	FVC	4,166 [h]	-0·09 (-0·12, -0·05)	2·98 x 10^{-7}	**5·46 x 10^{-7}**	4,618 [h]	-0·05 (-0·08, -0·02)	3·21 x 10^{-4}	**5·89 x 10^{-4}**
	FEV_1/FVC	4,166 [h]	-0·21 (-0·25, -0·18)	1·49 x 10^{-29}	**8·20 x 10-^{29}**	4,618 [h]	-0·12 (-0·16, -0·09)	3·95 x 10^{-14}	**2·17 x 10^{-13}**
Sensitivity - Smoking pack-years [g]	FEV_1	1,035 [i]	-0·23 (-0·30, -0·15)	4·28 x 10^{-9}	**1·18 x 10^{-8}**	1,170 [i]	-0·16 (-0·24, -0·09)	1·76 x 10^{-5}	**4·84 x 10^{-5}**
	FVC	1,035 [i]	-0·09 (-0·16, -0·03)	5·61 x 10^{-3}	**0·010**	1,170 [i]	-0·07 (-0·13, -0·01)	0·020	**0·036**
	FEV_1/FVC	1,035 [i]	-0·25 (-0·32, -0·17)	1·12 x 10^{-10}	**6·16 x 10^{-10}**	1,170 [i]	-0·18 (-0·25, -0·11)	2·03 x 10^{-7}	**1·12 x 10^{-6}**

[a] Z-score of pre-bronchodilator spirometry measurements obtained from applying the Global Lung Function Initiative equations; [b] Number of individuals with available genotype data and spirometry measurements included in the association analyses; [c] Effect size of the association as the change in z-score of lung function per one z-score increase in the PRS; [d] Adjusted *p*-value accounting for the false discovery rate. The Benjamini & Yekutieli method was applied across spirometry measurements per association model; [e] Basic association model, including ten PCs, the participation round, and the genotyping batch as covariates; [f] Sensitivity analyses accounting for first-hand smoking. Ten PCs, the participation round, the genotyping batch, and smoking status were included as covariates; [g] Sensitivity analyses accounting for active smoking were restricted to subjects with reported smoking habits. Ten PCs, the participation round, the genotyping batch, and smoking pack-years were included as covariates. Tobacco pack-years were calculated by multiplying the number of smoking years by the number of daily cigarettes and dividing by 20 cigarettes often contained in a package; [h] Number of individuals with available genotype data, spirometry measurements, and smoking status information included in the association analyses; [i] Number of individuals with reported active smoking habits and available genotype data, spirometry measurements, smoking status information included in the association analyses.

CI: confidence interval; FEV_1: forced expiratory volume in one second; FVC: forced vital capacity; PC: Principal Component of genetic ancestry.

Significant results (*q*-value ≤0·05) are highlighted in bold font.

Table S16. Results of the validation of the association with spirometry measurements in males and females from the Rotterdam Study.

Association model	Time point	Spirometry measurement [a]	***Males*** Sample size [b]	β (95%CI) [c]	*p*-value	*q*-value [d]	***Females*** Sample size [b]	β (95%CI) [c]	*p*-value	*q*-value [d]
Basic [e]	PFT 1 (50-98 years) [h]	FEV_1	2,524	-0·14 (-0·19, -0·09)	6·79 x 10^{-9}	**1·87 x 10^{-8}**	3,198	-0·16 (-0·20, -0·12)	7·47 x 10^{-13}	**2·05 x 10^{-12}**
		FVC		-0·06 (-0·10, -0·02)	7·59 x 10^{-3}	**0·014**		-0·07 (-0·11, -0·04)	1·29 x 10^{-4}	**2·37 x 10^{-4}**
		FEV_1/FVC		-0·14 (-0·19, -0·10)	1·18 x 10^{-11}	**6·49 x 10^{-11}**		-0·14 (-0·17, -0·10)	5·76 x 10^{-14}	**3·17 x 10^{-13}**
	PFT 2 (51-96 years) [h]	FEV_1	1,468	-0·15 (-0·21, -0·09)	2·98 x 10^{-7}	**8·20 x 10^{-7}**	1,849	-0·12 (-0·18, -0·07)	1·72 x 10^{-6}	**4·73 x 10^{-6}**
		FVC		-0·05 (-0·10, 0·00)	0·058	0·107		-0·04 (-0·08, 0·00)	0·055	0·100
		FEV_1/FVC		-0·18 (-0·23, -0·13)	2·43 x 10^{-12}	**1·34 x 10^{-11}**		-0·14 (-0·18, -0·10)	5·50 x 10^{-11}	**3·03 x 10^{-10}**
	PFT 3 (70-100 years) [h]	FEV_1	333	-0·26 (-0·38, -0·14)	3·19 x 10^{-5}	**8·77 x 10^{-5}**	408	-0·09 (-0·20, 0·01)	0·091	0·249
		FVC		-0·11 (-0·23, 0·00)	0·051	0·093		0·00 (-0·09, 0·09)	0·974	1
		FEV_1/FVC		-0·27 (-0·37, -0·16)	5·87 x 10^{-7}	**3·23 x 10^{-6}**		-0·13 (-0·22, -0·05)	2·97 x 10^{-3}	**0·016**
Sensitivity - Smoking status [f]	PFT 1 (50-98 years) [h]	FEV_1	2,501	-0·14 (-0·19, -0·09)	2·39 x 10^{-9}	**6·57 x 10^{-9}**	3,171	-0·16 (-0·20, -0·12)	5·30 x 10^{-13}	**1·46 x 10^{-12}**
		FVC		-0·06 (-0·10, -0·02)	6·72 x 10^{-3}	**0·012**		-0·07 (-0·11, -0·03)	1·44 x 10^{-4}	**2·64 x 10^{-4}**
		FEV_1/FVC		-0·15 (-0·19, -0·11)	2·42 x 10^{-12}	**1·33 x 10^{-11}**		-0·14 (-0·17, -0·10)	3·39 x 10^{-14}	**1·86 x 10^{-13}**
	PFT 2 (51-96 years) [h]	FEV_1	1,468	-0·15 (-0·21, -0·09)	2·41 x 10^{-7}	**6·63 x 10^{-7}**	1,849	-0·11 (-0·16, -0·06)	1·28 x 10^{-5}	**3·52 x 10^{-5}**
		FVC		-0·05 (-0·10, 0·00)	0·058	0·107		-0·04 (-0·08, 0·01)	0·092	0·168
		FEV_1/FVC		-0·18 (-0·22, -0·13)	1·50 x 10^{-12}	**8·25 x 10^{-12}**		-0·12 (-0·16, -0·09)	9·86 x 10^{-10}	**5·42 x 10^{-9}**
	PFT 3 (70-100 years) [h]	FEV_1	331	-0·25 (-0·37, -0·13)	5·71 x 10^{-5}	**1·57 x 10^{-4}**	408	-0·09 (-0·19, 0·02)	0·108	0·297
		FVC		-0·11 (-0·22, 0·00)	0·062	0·113		0·00 (-0·09, 0·09)	0·984	1
		FEV_1/FVC		-0·26 (-0·36, -0·15)	1·26 x 10^{-6}	**6·93 x 10^{-6}**		-0·12 (-0·21, -0·04)	3·99 x 10^{-3}	**0·022**
Sensitivity - Smoking pack-years [g]	PFT 1 (50-98 years) [h]	FEV_1	119	-0·08 (-0·27, 0·10)	0·393	1	144	-0·27 (-0·45, -0·10)	2·20 x 10^{-3}	**0·012**
		FVC		-0·01 (-0·17, 0·14)	0·879	1		-0·14 (-0·28, -0·01)	0·038	0·070
		FEV_1/FVC		-0·11 (-0·32, 0·10)	0·295	1		-0·23 (-0·39, -0·06)	8·08 x 10^{-3}	**0·022**
	PFT 2 (51-96 years) [h]	FEV_1	137	-0·26 (-0·46, -0·06)	0·010	**0·029**	205	-0·09 (-0·26, 0·08)	0·284	0·781
		FVC		-0·12 (-0·30, 0·07)	0·215	0·394		0·01 (-0·12, 0·15)	0·844	1
		FEV_1/FVC		-0·3 (-0·48, -0·12)	1·20 x 10^{-3}	**6·60 x 10^{-3}**		-0·17 (-0·32, -0·02)	0·029	0·157
	PFT 3 (70-100 years) [h]	FEV_1	18	-0·91 (-1·53, -0·29)	0·013	0·068	27	-0·54 (-0·87, -0·21)	4·20 x 10^{-3}	**0·012**
		FVC		-0·77 (-1·41, -0·12)	0·037	0·068		-0·21 (-0·51, 0·08)	0·173	0·317
		FEV_1/FVC		-0·68 (-1·24, -0·12)	0·033	0·068		-0·55 (-0·85, -0·24)	2·12 x 10^{-3}	**0·012**

[a] Z-score of pre-bronchodilator spirometry measurements obtained from applying the Global Lung Function Initiative equations; [b] Number of individuals with available genotype data and spirometry measurements included in the association analyses; [c] Effect size of the association as the change in z-score of lung function per one z-score increase in the PRS; [d] Adjusted *p*-value accounting for the false discovery rate. The Benjamini & Yekutieli method was applied across spirometry measurements per time point and association model; [e] Basic association model, including two PCs as covariates; [f] Sensitivity analyses accounting for active smoking. Two PCs and smoking status were included as covariates; [g] Sensitivity analyses accounting for active smoking restricted to subjects with reported smoking habits. Two PCs and tobacco pack-years were included as covariates; [h] Participants were grouped based on the time point when the pulmonary function test was conducted.

CI: confidence interval; FEV_1: forced expiratory volume in one second; FVC: forced vital capacity; PC: Principal Component of genetic ancestry. The results shown correspond to the evaluation of the association of the PRS for airflow limitation with spirometry measurements in each of the three time points when the pulmonary function test was conducted in the Rotterdam Study: PFT 1 (50-98 years), PFT 2 (51-96 years), and PFT 3 (70-100 years). The basic and active smoking-sensitivity association models were separately applied to males and females. Significant results (q-value ≤0·05) are highlighted in bold font.

Table S17. Association results of the PRS for airflow limitation with spirometry measurements excluding individuals with asthma patients in the BAMSE cohort.

Cohort	Spirometry measurement [b]	Time point [c]	Individuals included	Sample size	β (95%CI) [f]	*p*-value	*q*-value [g]
BAMSE-W1 [a]	FEV_1	8 years	Original [d]	335	-0·09 (-0·18, 0·01)	0·073	0·201
		8 years	Non-asthma [e]	229	-0·07 (-0·18, 0·04)	0·232	0·637
		16 years	Original [d]	305	-0·07 (-0·17, 0·03)	0·162	0·446
		16 years	Non-asthma [e]	214	-0·04 (-0·16, 0·08)	0·519	0·952
		24 years	Original [d]	282	-0·09 (-0·19, 0·00)	0·052	0·144
		24 years	Non-asthma [e]	219	-0·11 (-0·22, 0·00)	0·046	0·127
	FVC	8 years	Original [d]	335	0·02 (-0·07, 0·11)	0·659	1·000
		8 years	Non-asthma [e]	229	0·03 (-0·08, 0·14)	0·639	1·000
		16 years	Original [d]	305	0·02 (-0·08, 0·12)	0·67	1·000
		16 years	Non-asthma [e]	214	0·07 (-0·05, 0·19)	0·282	0·775
		24 years	Original [d]	282	0·01 (-0·08, 0·11)	0·782	1·000
		24 years	Non-asthma [e]	219	0·02 (-0·08, 0·13)	0·692	1·000
	FEV_1/FVC	8 years	Original [d]	335	-0·15 (-0·24, -0·06)	1·81 x 10^{-3}	**0·010**
		8 years	Non-asthma [e]	229	-0·14 (-0·25, -0·03)	0·011	0·060
		16 years	Original [d]	305	-0·12 (-0·23, -0·01)	0·040	0·218
		16 years	Non-asthma [e]	214	-0·15 (-0·28, -0·02)	0·025	0·140
		24 years	Original [d]	282	-0·16 (-0·26, -0·06)	2·56 x 10^{-3}	**0·014**
		24 years	Non-asthma [e]	219	-0·21 (-0·32, -0·09)	6·71 x 10^{-4}	**3·69 x 10^{-3}**
BAMSE-W2 [a]	FEV_1	8 years	Original [d]	1,230	-0·12 (-0·17, -0·06)	1·55 x 10^{-5}	**4·26 x 10^{-5}**
		8 years	Non-asthma [e]	1,144	-0·12 (-0·18, -0·07)	1·08 x 10^{-5}	**2·97 x 10^{-5}**
		16 years	Original [d]	1,185	-0·14 (-0·20, -0·08)	8·07 x 10^{-7}	**2·22 x 10^{-6}**
		16 years	Non-asthma [e]	1,035	-0·11 (-0·17, -0·05)	2·70 x 10^{-4}	**7·44 x 10^{-4}**
		24 years	Original [d]	1,044	-0·12 (-0·17, -0·07)	9·70 x 10^{-6}	**2·67 x 10^{-5}**
		24 years	Non-asthma [e]	940	-0·11 (-0·17, -0·06)	9·37 x 10^{-5}	**2·58 x 10^{-4}**
	FVC	8 years	Original [d]	1,230	-0·03 (-0·08, 0·02)	0·214	0·393
		8 years	Non-asthma [e]	1,144	-0·04 (-0·10, 0·01)	0·101	0·184
		16 years	Original [d]	1,185	-0·02 (-0·08, 0·03)	0·459	0·842
		16 years	Non-asthma [e]	1,035	0·00 (-0·06, 0·05)	0·908	1·000
		24 years	Original [d]	1,044	-0·02 (-0·07, 0·04)	0·558	1·000
		24 years	Non-asthma [e]	940	-0·01 (-0·06, 0·05)	0·743	1·000
	FEV_1/FVC	8 years	Original [d]	1,230	-0·13 (-0·18, -0·08)	5·64 x 10^{-7}	**3·10 x 10^{-6}**
		8 years	Non-asthma [e]	1,144	-0·12 (-0·17, -0·07)	4·69 x 10^{-6}	**2·58 x 10^{-5}**
		16 years	Original [d]	1,185	-0·17 (-0·23, -0·12)	7·80 x 10^{-10}	**4·29 x 10^{-9}**
		16 years	Non-asthma [e]	1,035	-0·15 (-0·21, -0·09)	2·88 x 10^{-7}	**1·58 x 10^{-6}**
		24 years	Original [d]	1,044	-0·15 (-0·20, -0·09)	6·25 x 10^{-8}	**3·44 x 10^{-7}**
		24 years	Non-asthma [e]	940	-0·15 (-0·20, -0·09)	1·99 x 10^{-7}	**1·09 x 10^{-6}**

[a] The PRS for airflow limitation was separately estimated in each of the genotyping Waves of the BAMSE cohort (Wave 1, n=463; Wave 2, n=2,173); [b] Z-score of pre-bronchodilator spirometry measurements obtained from applying the Global Lung Function Initiative equations; [c] Average age of the individuals at the data collection follow-ups included in this study; [d] Subjects with available genotype data and spirometry measurements included in the original basic association model; [e] Subjects with available genotype data and spirometry measurements included in the original basic association model (after excluding asthma patients). Asthma was defined as a doctor's diagnosis and any symptoms with breathing difficulties or occasional or regular use of asthma medications in the last 12 months; [f] Effect size of the association as the change in z-score of lung function per one z-score increase in the PRS; [g] Adjusted *p*-value accounting for the false discovery rate. The Benjamini & Yekutieli method was applied across spirometry measurements per time point, set of individuals included, and cohort.

CI: confidence interval; FEV_1: forced expiratory volume in one second; FVC: forced vital capacity; PRS: polygenic risk score; W1: Genotyping Wave 1; W2: Genotyping Wave 2.

Results are shown for the basic association model (adjusting by two Principal Components of genetic ancestry) in the original set of subjects and excluding individuals with asthma.

Significant results (*q*-value ≤0·05) are highlighted in bold font.

Table S18. Results of the association of the PRS for airflow limitation with asthma status in the BAMSE cohort.

Cohort	Time point [b]	Sample size	β (95%CI) [c]	*p*-value
BAMSE-W1 [a]	8 years	334	0·19 (-0·04, 0·43)	0·103
	16 years	299	0·22 (-0·03, 0·47)	0·084
	24 years	281	0·18 (-0·10, 0·46)	0·214
BAMSE-W2 [a]	8 years	1,223	0·08 (-0·14, 0·30)	0·476
	16 years	1,176	-0·01 (-0·19, 0·16)	0·882
	24 years	1,042	0·03 (-0·18, 0·23)	0·807

[a] The PRS for airflow limitation was separately estimated in each of the genotyping Waves of the BAMSE cohort (Wave 1, n=463; Wave 2, n=2,173); [b] Average age of the individuals at the data collection follow-ups included in this study; [c] Effect size of the association.
CI: confidence interval; FEV_1: forced expiratory volume in one second; FVC: forced vital capacity; PRS: polygenic risk score; W1: Genotyping Wave 1; W2: Genotyping Wave 2.
Results are shown for the evaluation of the association of the PRS for airflow limitation with a binary variable of asthma status including two Principal Components of genetic ancestry as covariates. Asthma patients were considered in case of a doctor's diagnosis and any symptoms with breathing difficulties or occasional or regular use of asthma medications in the last 12 months.
